# TRIB1 confers therapeutic resistance in GBM cells by activating the ERK and Akt pathways

**DOI:** 10.1038/s41598-023-32983-w

**Published:** 2023-08-01

**Authors:** Karnika Singh, Chunhua Han, Jessica L. Fleming, Aline P. Becker, Joseph McElroy, Tiantian Cui, Benjamin Johnson, Ashok Kumar, Ebin Sebastian, Christian A. Showalter, Morgan S. Schrock, Matthew K. Summers, Valesio Becker, Zhen-yue Tong, Xiaomei Meng, Heather R. Manring, Monica Venere, Erica H. Bell, Pierre A. Robe, A. L. Grosu, S. Jaharul Haque, Arnab Chakravarti

**Affiliations:** 1 Department of Radiation Oncology, The Ohio State University Comprehensive Cancer Center, Columbus, OH 43210 USA; 2 Department of Biomedical Informatics, Center for Biostatistics, The Ohio State University, Columbus, OH 43210 USA; 3 Department of Radiation Oncology, City of Hope, Duarte, CA 91010 USA; 4 Corewell Health William Beaumont University Hospital, Royal Oak, MI 48073 USA; 5 Neroscience Research Institute/Department of Neurology, The Ohio State University Comprehensive Cancer Center, Columbus, OH 43210 USA; 6 Department of Neurology and Neurosurgery, Brain Center Rudolf Magnus, University Medical Center Utrecht, 3584 CG Utrecht, The Netherlands; 7 Freiburg University, 79098 Freiburg, Germany

**Keywords:** CNS cancer, Prognostic markers

## Abstract

GBM (Glioblastoma) is the most lethal CNS (Central nervous system) tumor in adults, which inevitably develops resistance to standard treatments leading to recurrence and mortality. TRIB1 is a serine/threonine pseudokinase which functions as a scaffold platform that initiates degradation of its substrates like C/EBPα through the ubiquitin proteasome system and also activates MEK and Akt signaling. We found that increased *TRIB1* gene expression associated with worse overall survival of GBM patients across multiple cohorts. Importantly, overexpression of TRIB1 decreased RT/TMZ (radiation therapy/temozolomide)-induced apoptosis in patient derived GBM cell lines in vitro. TRIB1 directly bound to MEK and Akt and increased ERK and Akt phosphorylation/activation. We also found that TRIB1 protein expression was maximal during G2/M transition of cell cycle in GBM cells. Furthermore, TRIB1 bound directly to HDAC1 and p53. Importantly, mice bearing TRIB1 overexpressing tumors had worse overall survival. Collectively, these data suggest that TRIB1 induces resistance of GBM cells to RT/TMZ treatments by activating the cell proliferation and survival pathways thus providing an opportunity for developing new targeted therapeutics.

## Introduction

Glioblastoma (GBM; WHO grade 4) is the most common primary malignancy of the brain in adults. It is the most aggressive, undifferentiated and invasive tumor of the highest mortality with median survival of 12–15 months after diagnosis^1,2^. The current standard of care treatment for GBM is maximal safe surgical resection plus concomitant radiotherapy (RT) and temozolomide (TMZ) chemotherapy followed by adjuvant TMZ treatment in accordance with the Stupp protocol^3,4^. Despite this aggressive, multi-modal treatment, development of resistance and subsequent recurrence of tumor remain inevitable due to a variety of factors including intra-tumoral heterogeneity and clonal evolution, prevalence of glioma stem cells, activation of oncogenic pathways, suppression of anti-tumor-immunity, and modulation of tumor cell metabolism among others^5–7^. In 2016, WHO revised the classification of malignant gliomas based on *IDH* status (mutant and wild type) with concomitant presence of additional genetic alterations^8^. More than 90% of GBM patients harbor wild-type *IDH1/2* who have worse survival outcome compared to patients with mutant *IDH1/2*^9^. In 2021, WHO re-classified grade 4 tumors to be GBM only if they contained wild-type *IDH1/2* independent of the histological grade; otherwise, they are to be considered as *IDH* mutant astrocytomas (grades 2–4)^10^. To identify novel oncogenic pathways that drive treatment resistance and subsequent disease recurrence in GBM, we undertook multi-omics molecular profiling approaches and found a number of differentially expressed genes, one of which was *TRIB1*.

TRIB1, a protein pseudokinase is one of the three members of its family named after resemblance to the *Drosophila* gene tribbles (*Trbl*)^11^. They function as signaling scaffolds for protein–protein interactions^12^. TRIBs are composed of an N-terminal domain, a pseudokinase domain and a C-terminal domain^13^. As opposed to a conventional protein kinase, they lack the canonical ATP binding DFG motif and instead contain the SLE sequence in their pseudokinase domain^14^. TRIB1 exists in two confirmations; a closed ‘SLE-out’’ confirmation where the C-terminal domain is folded back on its pseudokinase domain and an open ‘SLE-in’ confirmation in which the binding of substrate to the pseudokinase domain releases the folded C-terminal domain opening it for further substrate binding^15^. C/EBPα is the most characterized substrate of TRIB1. It belongs to the C/EBP family of transcription factors and plays a role in differentiation and cell cycle arrest^16,17^. It has been shown that C/EBPα binds to the pseudokinase domain on TRIB1 where it is targeted for degradation by the COP1 E3 ubiquitin ligase recruited to the conserved motif (DQIVPE) on the C-terminal domain of TRIB1^18^. Interestingly, another C-terminal motif, ILLHPW has been shown to bind MEK1 and activate ERK signaling^19^.

TRIB1 has been implicated in a number of human malignancies including acute myeloid leukemia (AML), non-small cell lung carcinoma (NSCLC), prostate cancer, hepatocellular carcinoma (HCC) and colorectal cancer (CRC)^20–24^. For example, in AML, elevated TRIB1 levels lead to decreased C/EBPα levels in myeloid cells leading to an accumulation of undifferentiated myeloid progenitors in the bloodstream. Furthermore, TRIB1 increases MAPK signaling in these cells to stimulate tumor growth^20^. It has been shown that TRIB1 associates with HDAC1 and inactivates p53 in NSCLC^21^. TRIB1 also supports spheroid cell growth in prostate cancer cells^22^. TRIB1 promotes cell invasion and migration in HCC^23^ and CRC^24^. In vivo studies in the above cancer models have reported that downregulation of TRIB1 causes reduced tumor growth suggesting that TRIB1 plays a role in tumor initiation and maintenance though multiple mechanisms in a cell specific manner.

In this study, we have identified and validated TRIB1 as a potential novel therapeutic target in GBM. We observed that increased TRIB1 gene expression correlated with worse overall survival (OS) in TCGA GBM, TCGA low grade glioma (LGG) and institutional patient cohort 2. Our in vitro studies revealed that overexpression of TRIB1 decreased apoptosis in response to RT/TMZ treatment. Mechanistically, we demonstrate that TRIB1 upregulated the ERK- and Akt signaling modules promoting survival of RT/TMZ -treated GBM cells in vitro. We also determined that TRIB1 protein expression peaked during G2/M phase of the cell cycle and TRIB1 was also involved in HDAC1 and COP1 mediated regulation of p53 in GBM cells. Finally, TRIB1 overexpression increased tumor growth in an orthotopic xenograft mouse model of GBM suggesting that TRIB1 plays oncogenic role(s) in malignant gliomas.

## Materials and methods

### Cell lines and cell culture

Human primary GBM patient derived cell lines, T08-387 and GBM3359 (3359) were obtained from Dr. Jeremy Rich (The University of Pittsburg). The GBM30-luc cells were provided by Dr. Balveen Kaur (The University of Texas MD Anderson Cancer Center). These cell lines were authenticated by DNA profiling and passaged in nude mice. These cell lines were maintained as a suspension culture in neurobasal medium (Gibco) supplemented with EGF (20 ng/ml, R&D systems), FGF (20 ng/ml, R&D systems), sodium pyruvate (1×, Gibco), glutamate (1×, Gibco), 100 U/ml penicillin–streptomycin (Sigma) and B27 (1×) supplement (Gibco). Human GBM cell lines, U87 MG (HTB-14) and LN18, and HEK293 and HEK293T were obtained from ATCC. U87 MG and HEK293 cells were cultured in EMEM and 10% (v/v) FBS (Invitrogen). LN18 and HEK293T cells were cultured in DMEM, 100 U/ml penicillin–streptomycin (Sigma) and 10% (v/v) FBS (Invitrogen). All cell lines were maintained in a humidified chamber with 5% CO_2_. 3359-*TP*53 R248W cells were created using CRISPR protocol described previously^25^.

### Patient cohorts and ethics statement

Tumor biopsy or resection was performed on newly diagnosed glioblastoma patients at Utrecht Medical Center (Utrecht, Netherlands) during 2005 to 2014 (Institutional cohort 1). Paraffin blocks were obtained from Freiburg, Germany corresponding to 33 patients in the cohort (Institutional cohort 2). After neuropathology review, representative areas (> 70% tumor) were selected for DNA isolation. Clinical data including pre-treatment patient characteristics, treatment information, and survival outcomes were collected and stored in a centralized database. The patients were > 18 years of age and histologically diagnosed grade II–IV. Patients in Utrecht cohort were treated mainly with Stupp protocol. Patients in Freiburg cohort were treated either with RT or chemotherapy or both. Patient demographics are indicated in Supplementary Table 1. Informed consent was obtained from all subjects. This study was approved and performed in accordance with regulations of the institutional review boards of University of Freiburg, Utrecht University Medical Center and The Ohio State University. Data for the two institutional cohorts are available in supplementary data file. Other data is publicly available through TCGA (The Cancer Genome Atlas). All non-public data generated or analyzed during this study are included in this published article [and its supplementary information files].

### DNA isolation

Total DNA was isolated from the tumor specimens (1 mm core punches) as described previously^26^. FFPE (fresh frozen paraffin embedded) tumor specimens from 203 newly diagnosed GBM patients were used. In brief, core punches were deparaffinized using xylene and ethanol washes. Tissue was digested for 48 h using digestion buffer from the RecoverAll™ Total Nucleic Acid Isolation Kit for FFPE (AM1975, Invitrogen). DNA was isolated using the MasterPure™ Complete DNA and RNA Purification Kit (MC8520, Illumina). DNA was quantified using the Qubit™ dsDNA HS Assay Kit (Q32854, Invitrogen).

### mRNA profiling

Clariom™ D Assay, human (catalog number 902922, Applied Biosystems) was utilized for mRNA profiling of patients from Freiburg LGG cohort. RNA was isolated as previously described^26^. RNA input of 25–100 ng was used for the cDNA reaction. The SST-RMA algorithm was used for data normalization and producing gene level expression (http://media.affymetrix.com/support/technical/whitepapers/sst_gccn_whitepaper.pdf).

### Methylation analysis

DNA extracted from 203 newly diagnosed GBM patients were analyzed by Illumina 450 K or EPIC methylation array at the University of Southern California Molecular Genomics Core (Los Angeles, CA). Methylation data were processed and normalized (NOOB) using the minfi package^27^ in R (https://www.R-project.org/). Data corresponding to probe cg00207280 (targets 1500nt upstream of transcription start site on TRIB1 promoter, TSS1500) is shown.

### Irradiation

Cells were exposed to indicated doses of radiation per day at room temperature using the X-Rad cabinet system. Total dose was administered to the cells in about 2.5–5 min.

### Cell viability assay

500–1000 cells were seeded in 96-well plates and treated with various doses of IR or concentrations of TMZ. Cell viability was determined using the Cell-Titer-Glo Luminescent Cell Viability Assay (Promega, G7571) after respective time points according to the manufacturer’s instructions. Luminescence was recorded using a Luminescence plate reader. The experiments were performed in replicates of three or more.

### Western blotting

Cells were lysed in RIPA (radioimmunoprecipitation assay) lysis buffer (Thermo Scientific, 89900) containing Halt™ Protease and Phosphatase Inhibitor Cocktail (Thermo Scientific, 78442). Protein concentration was determined using the Pierce™ BCA protein assay kit (Thermo Scientific, 23225). Equal amounts of protein were loaded and separated using a 4–15% SDS-PAGE gel (Bio-Rad) and transferred to nitrocellulose membranes (Amersham). Membranes were blocked with 5% nonfat dry milk in TBST, incubated with respective primary antibodies overnight at 4 °C, followed by respective secondary antibody incubation for 1–2 h at room temperature. Proteins were detected using Immobilon Western Chemiluminescent HRP Substrate (Millipore) and imaged using the Amersham Imager. Blots were washed three times between primary and secondary antibody incubation and prior to detection with chemiluminescent substrate. List of primary and secondary antibodies used is provided in Supplementary Tables 2 and 3, respectively.

### Plasmid constructs

The *TRIB1* expression plasmid was obtained from GenScript (Clone ID: OHu22886) and system biosciences (SBI) by providing custom sequence (Supplementary Methods 1). The doxycycline inducible shRNA was purchased from Dharmacon (SMARTvector Inducible Human TRIB1 mCMV-TurboRFP shRNA). The *TRIB1* deletion mutant constructs were a generous gift from Dr. Takuro Nakamura (Division of Carcinogenesis, The Cancer Institute, Tokyo, Japan). *TRIB1*-W337A-FLAG and *TRIB1*-W337A-MYC plasmids were generated by mutating the MEK binding site in respective *TRIB1* expression plasmids using QuikChange Lightning Site-Directed Mutagenesis Kit (Agilent technologies) according to manufacturer’s instructions. The primer sequence is indicated in supplementary methods 2. Control shRNA plasmid-A (sc-108060) and COP1 shRNA Plasmid (h) (sc-45541-SH) were purchased from Santa Cruz biotechnologies.

### Reagents

Trametinib (GSK1120212) (Catalog No.S2673), AZD6244 (Selumetinib) (Catalog No.S1008), perifosine (KRX-0401) (Catalog No.S1037), wortmannin (KY12420) (Catalog No.S2758), and Ro-3306 (Catalog No. S7747) were purchased from Selleckchem. Thymidine powder (T1895) was purchased from Sigma.

### Viral transduction

Cells were infected with TRIB1 doxycycline inducible shRNA at MOI of 5 for 24 h in the presence of 6 µg/ml polybrene. After 24 h the culture medium was changed. Cells were selected 24 h later by Puromycin treatment (2 µg/ml for T08-387 and 10 µg/ml for GBM30). Single clones were selected using the limiting dilution assay. The successfully transfected cells were selected using 2 µg/ml puromycin and validated by western blot. For inducing knockdown, cells were treated with 2 µg/ml doxycycline treatment for 48 h unless otherwise mentioned.

### Cell line preparation

1–6 µg of respective plasmids were transfected using Lipofectamine 2000 transfection reagent (Invitrogen). Stable cell lines were selected and cultured in complete Neurobasal medium with Puromycin or G418. The overexpression or knockdown cell lines used in this study are stable cell lines unless otherwise mentioned.

### Co-immunoprecipitation

Total cell lysates were prepared using the IP Lysis buffer supplied in the Pierce Co-Immunoprecipitation (Co-IP) Kit (26149). The immunoprecipitation was performed according to manufacturer’s instructions using FLAG antibody. In addition, Pierce Anti-DYKDDDDK Magnetic Agarose Beads (A36797) and Pierce Anti-c-Myc Magnetic Beads (88842) were used for immunoprecipitation following the manufacturer’s protocol. The elution was also performed according to manufacturer’s protocol. The obtained eluate was then probed with respective primary antibodies following the western blotting protocol described previously. Lysates from cells not expressing tagged TRIB1 were used as negative control and are shown in Supplementary Fig. 7.

### Cell cycle analysis

One million cells were suspended in 1 ml of PBS. The suspended cells were fixed on ice in 9 ml of 70% ethanol. The cells were then incubated at 4 °C for 2 h followed by centrifugation for 5 min at 500*g*. The cells were resuspended in 5 ml PBS and again centrifuged. The obtained cell pellet was stained with 300 µl propidium iodide (PI) solution (0.1% Triton X-100, 10 µg/ml PI, 100 µg/ml DNase-free RNase A), for 30 min in dark at room temperature. The cells were analyzed by flow cytometry using BD LSR II equipped with BD FACSDiva 9.0 software for acquisition and analysis. FlowJo software was used for further analysis of obtained data. The gating strategy has been outlined in Supplementary Fig. 6g.

### Intracranial mouse model and survival analysis

Five- to six-week-old female athymic nude mice, obtained from Jackson Laboratory (J:NU (007850, JACKSON) HOMOZYGOUS), were used for the intracranial xenograft mouse experiments. Female mice were used in these studies for logistical reasons. We do not believe sex would have an effect on the outcome of these studies, but they can be repeated in male mice at a later time if we have reason to believe the outcome would be different in male mice. Since only female mice were used, no sex-based analyses were performed. The study did not involve animals collected from the field or from wild. The mice were anaesthetized and fixed on a stereotactic apparatus. Along the frontal suture line, a 4 mm incision was made on the scalp and a burr hole was drilled 2 mm lateral and 1 mm anterior to the bregma. T08-387 cells expressing FLAG-empty vector, *TRIB1*-FLAG and *TRIB1*-W337A-FLAG were injected (500,000 cells in 2 µl PBS) into the hole with a 7 × Hamilton 80,300 syringe at a depth of 3 mm. Mouse survival was considered until removal criteria (greater than twenty percent weight loss, breathing difficulties or development of neurological deficits affecting the ability of the mouse to eat, drink, or move freely) was met. All procedures were approved by the subcommittee on Research Animal Care at The Ohio State University. All methods were carried out in accordance with relevant guidelines and regulations. All methods are reported in accordance with ARRIVE guidelines.

### MRI imaging and analysis

Tumor volumes were monitored by MRI imaging at the Ohio State University (OSU) small animal imaging core. Animals were anesthetized with 2.5% isoflurane mixed with 1 l/min carbogen (95% O_2_ with 5% CO_2_) then maintained with 1% isoflurane. Physiologic parameters including respiration and temperature were monitored using a small animal monitoring system (Model 1025, Small Animals Instruments, Inc. Stony Brook, NY). A pneumatic pillow was used to monitor respiration. Core temperature was maintained using a heated mouse holder. Imaging was performed using a Bruker BioSpin 94/30USR magnet (Bruker Biospin, Karlsruhe, Germany) and a phased array mouse brain and a 72 mm diameter volume as receiver and transceiver coils, respectively. Mice were injected with the Gadolinium-based contrast agent (100 μl/20 g body weight, in 0.1 M MultiHance, i.p.) 10–15 min prior to T1-w acquisition. Rapid imaging with refocused echoes (RARE) sequence was performed with the following parameters: TR = 1500 ms, TE = 9 ms, rare factor = 4, NA = 6, FOV = 20 mm × 15.3 mm, slice thickness = 0.5 mm, matrix size = 256 × 1966, 30 number of slices. For data analysis, a region of interest (ROI) that included the tumor (hyperintense regions) was manually outlined and tumor volumes were calculated using the ITK SNAP software. Data analysis and MRI imaging was performed in a blinded manner.

### Statistical analysis

For ‘omics survival analyses, Kaplan–Meier curves with log-rank tests were employed for univariable analyses using the R programming language (https://www.r-project.org/). For the Kaplan–Meier plots, TRIB1 expression was median dichotomized for visualization purposes. Cox regression was used to perform multivariable analysis. Co-variables included in the analysis were gender (categorical), age (continuous), KPS-70 KPS (categorical; < 70 vs 70 and above), treatment (categorical; “none”, “monotherapy TMZ or radiation only”, or “TMZ + radiation (Stupp protocol)”) and *TRIB1* gene expression/mRNA levels. RT and TMZ were transformed as time dependent variables. All experiments were performed at least twice or unless otherwise mentioned. GraphPad prism was used to perform statistical analysis by one way ANOVA with Tukey’s multiple comparison test. The error bars represent SD. Western blots were quantified by ImageJ and expressed as (average ± SEM) in the text.

## Results

### Elevated *TRIB1* gene expression is associated with worse OS in malignant glioma patients

**Figure 1:**
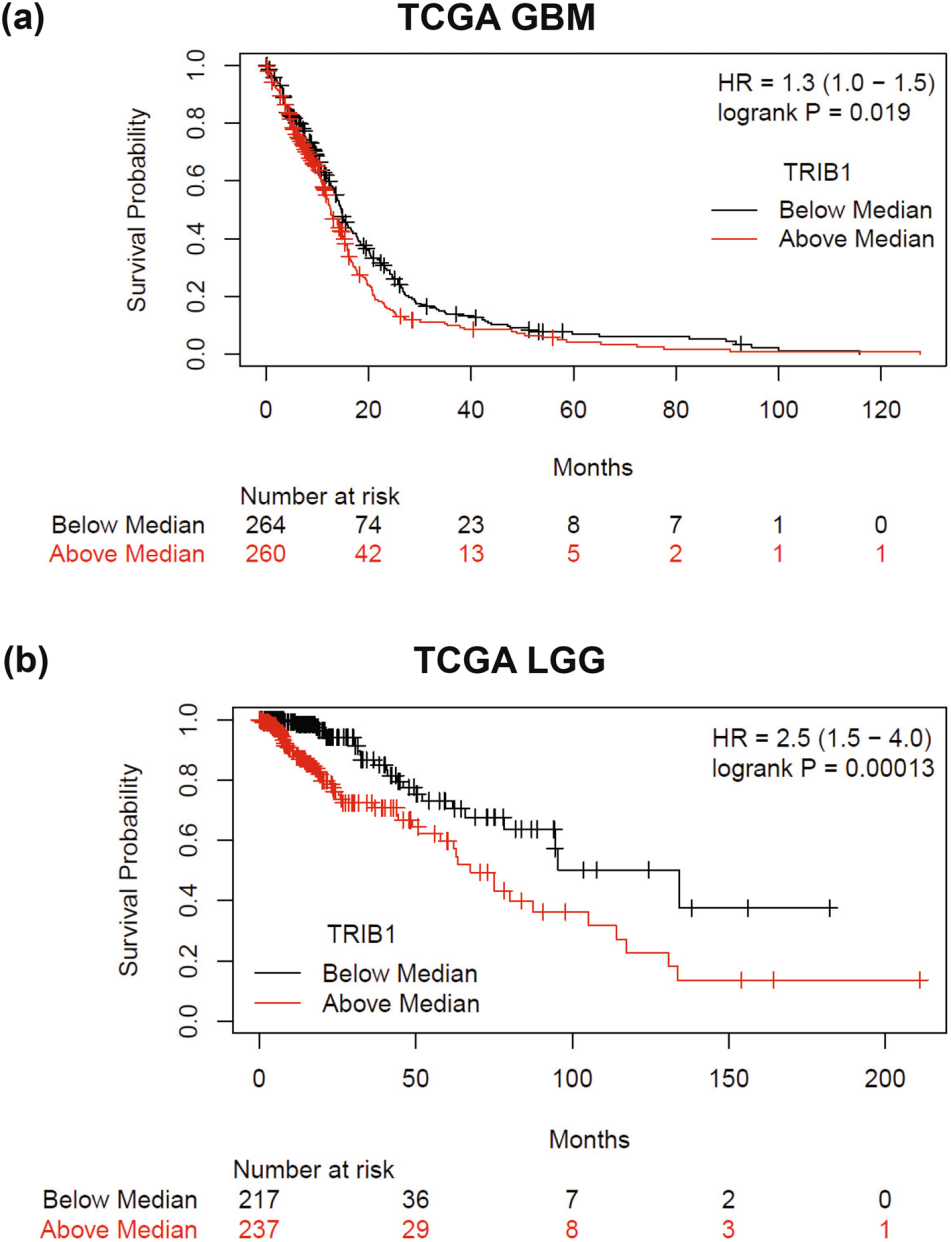
Increased TRIB1 gene expression correlate with worse OS of patients. Kaplan Meier curves show the correlation of TRIB1 gene expression with survival of patients in (**a**) TCGA GBM, HR 1.3 (1.0–1.5) and (**b**) TCGA LGG, HR 2.5 (1.5–4.0) cohorts.

To identify new therapeutically vulnerable targets in GBM, we utilized a patient-centered reverse translational approach in which we performed multi-omics analyses on diffuse glioma samples^28^. In our institutional cohort 1, univariate analysis revealed that methylation of a single CpG site (chr8:126441471–126443552) within the *TRIB1* promoter significantly correlated with better patient OS (Supplementary Fig. 1a). To further evaluate TRIB1 as a biomarker in glioma, we looked at the association of *TRIB1* gene expression with clinical outcomes using respective GBM and LGG patient cohorts. Increased *TRIB1* gene expression correlated with significantly worse OS in both GBM (HR 1.3 (1.0–1.5); P = 0.019) (Fig. 1a) and LGG patients (HR 2.5 (1.5–4); P = 0.00013) (Fig. 1b and Supplementary Fig. 1b). Multivariable analysis in the above cohorts showed that *TRIB1* gene expression correlated with worse overall survival independent of clinical variables (TCGA GBM: HR 1.181 (1.37–1.017), p = 0.029 ad TCGA LGG: HR 1.786 (2.617–1.219, p = 0.003) Tables 1 and 2 respectively). Additionally, *TRIB1* expression levels were found to be elevated in *IDH1/2*-wild type patients compared to *IDH1/2*-mutant LGG patients (Supplementary Fig. 1c,d). Taken together, these correlative studies suggest that TRIB1 may serve as a potential prognostic marker for malignant gliomas. Further studies are needed to validate these findings.

**Table 1:** Multivariable analyses of clinicopathologic parameters of overall survival in patient with TCGA GBM.

	Coef	Hazard Ratio	CI (lower)	CI (upper)	SE (coef)	z	Pr(>|z|)
Sex: male	0.1701	1.185	0.9305	1.51	0.1235	1.377	0.168
Age_Years	0.02731	1.028	1.018	1.038	0.005026	5.433	< 0.001***
RT.rc*dat2$Time.Split==1TRUE	− 2.192	0.1117	0.06607	0.1888	0.2678	− 8.186	< 0.001***
RT.rc*dat2$Time.Split==2TRUE	− 0.668	0.5128	0.2306	1.14	0.4077	− 1.638	0.101
RT.rc*dat2$Time.Split==3TRUE	0.2219	1.248	0.4529	3.441	0.5173	0.429	0.668
TMT.rc*dat2$Time.Split==1TRUE	− 0.2663	0.7662	0.3035	1.934	0.4725	− 0.5636	0.573
TMT.rc*dat2$Time.Split==2TRUE	− 1.618	0.1983	0.04815	0.8166	0.7221	− 2.241	0.025*
TMT.rc*dat2$Time.Split==3TRUE	− 0.2005	0.8183	0.4447	1.506	0.3111	− 0.6443	0.519
KPS.CODE: >= 70	− 0.5015	0.6056	0.4527	0.8102	0.1485	− 3.377	0.001***
TRIB1	0.166	1.181	1.017	1.37	0.07604	2.183	0.029*

**Table 2:** Multivariable analyses of clinicopathologic parameters of overall survival in patient with TCGA LGG.

	Coef	Hazard ratio	CI (lower)	CI (upper)	SE (coef)	z	Pr(>|z|)
Sex: male	− 0.2443	0.7833	0.4527	1.355	0.2797	− 0.8733	0.383
Age_Years	0.05282	1.054	1.029	1.08	0.01231	4.291	< 0.001***
RT: Radiation	0.1811	1.199	0.5625	2.554	0.386	0.4692	0.639
TMT: TMT	0.5189	1.68	0.9157	3.083	0.3097	1.676	0.094
KPS.CODE: >= 70	− 2.444	0.0868	0.03068	0.2456	0.5307	− 4.605	< 0.001***
TRIB1	0.5802	1.786	1.219	2.617	0.1949	2.977	0.003**

### Effects of RT and TMZ on TRIB1 expression levels in GBM cells

**Figure 2:**
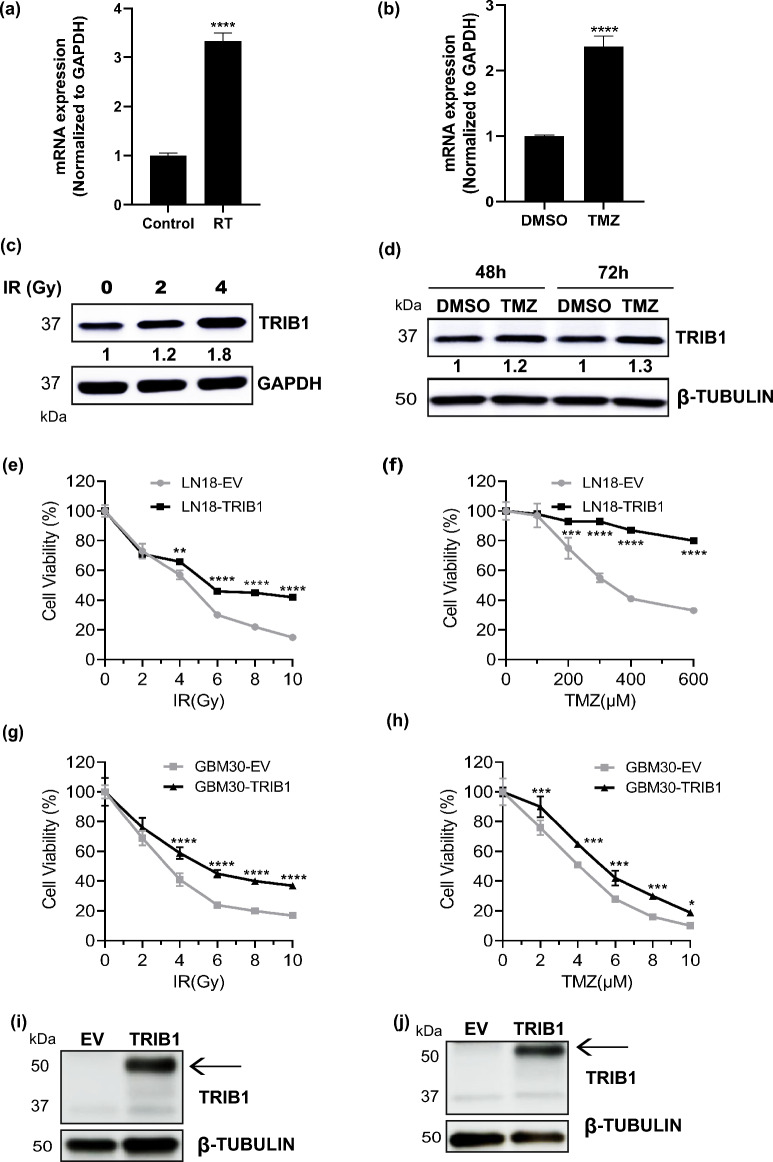
RT and TMZ induce *TRIB1* mRNA and protein levels and TRIB1 overexpression increases cell viability. Real time PCR results showed increased *TRIB1* mRNA levels with (**a**) RT (2 Gy, 4 days) (n = 2) and (**b**) TMZ treatment (300 μM, 72 h) in LN18 cells (n = 2). The mRNA levels are shown relative to unirradiated (control) or DMSO treated cells respectively. Representative western blots showing TRIB1 protein levels after indicated (**c**) RT, 4 days (n = 2) and (**d**) TMZ treatment (100 μM, n = 3) in LN18 cells. Two separate western blots were run for (**c**) and one blot was cut into two for (**d**). Line graphs depicting the cell viability of LN18 empty vector cells (LN18-EV) and TRIB1 overexpression cells (LN18-TRIB1) after (**e**) RT and (**f**) TMZ treatment (n = 3). Line graphs depicting the cell viability of GBM30 empty vector cells (GBM30-EV) and TRIB1 overexpression cells (GBM30-TRIB1) after (**g**) RT and (**h**) TMZ treatment (n = 3). Western blots confirming TRIB1 overexpression in LN18 (**i**) and GBM30 (**j**) cells used for above experiments. Arrow indicates TRIB1 overexpression. Tukey's multiple comparison test was performed to determine significance between respective concentrations of EV and TRIB1. *p < 0.05, **p < 0.01, ***p < 0.001, ****p < 0.0001.

Because all GBM patients receive standard treatment, it is important to examine if RT and/or TMZ regulate the expression of TRIB1 in GBM cells. To address this, LN18 cells were irradiated once daily with 2 Gy for 4 days or treated with 300 µM TMZ for 72 h which showed that *TRIB1* mRNA levels were increased significantly (threefold) after sequential 2 Gy irradiation for 4 days (Fig. 2a). Similarly, an increase (up to 2.5-fold) in *TRIB1* mRNA was observed after 300 µM TMZ treatment at 72 h (Fig. 2b). A similar effect was observed at the protein level after irradiation of LN18 cells with 2 and 4 Gy once daily for 4 days where, TRIB1 protein increased 1.2- (± 0.08) and 1.8-fold (± 0.15) respectively (Fig. 2c). Treatment with 100 µM TMZ also led to an increase in TRIB1 protein by 1.2- or 1.3-fold after 24 and 48 h respectively (Fig. 2d).

Next, we sought to determine what role TRIB1 plays in GBM pathobiology employing RNA interference (RNAi) in preclinical xenograft models. To this end we made multiple attempts to generate constitutive TRIB1-knockdown (KD) cells but consistently failed, which suggested that TRIB1 expression was required for the survival of GBM cells in vitro. Using the reciprocal approach, we stably overexpressed TRIB1 in LN18, GBM30 and T08-387 cells and used these cells to address the role of TRIB1 in GBM pathobiology. Cells were treated with indicated doses of radiation and indicated concentrations of TMZ, and cell viability was measured four days later. The results showed that TRIB1 overexpressing cells had significantly greater viability compared to empty vector transfected cells after most doses of radiation (Fig. 2e,g and Supplementary Fig. 2). TMZ treatment also had a similar effect where LN18 cells overexpressing TRIB1 had threefold greater viability compared to empty vector transfected cells (Fig. 2f). However, in the GBM30 cell line the difference in cell viability was less apparent but still significantly enhanced with TRIB1 overexpression (Fig. 2h).

We observed that the overexpressed TRIB1 protein had an increased molecular weight (50 kDa) compared to the endogenous protein (37 kDa) (shown in full blot section). There could be multiple reasons for this difference like alternate translation start site on *TRIB1* mRNA, unknown splice variants, and proteolytic cleavage of TRIB1 among others. The endogenous TRIB1 has been shown to have at least two isoforms. The longer isoform (NP_079471.1) is 367 amino acids long and has a molecular weight of 41 kDa, and the shorter isoform (NP_001269914.1) is 206 amino acids long (lacking the first 166 amino acids) and 23.48 kDa in molecular weight. It has also been observed that transcription of the endogenous *TRIB1* can be initiated from multiple sites in a cell type specific manner^29^. Commercially available antibodies also detect multiple TRIB1 protein bands of different sizes, which is consistent with other reports suggesting that the transcriptional processing of exogenous *TRIB1* mRNA is different from the endogenous mRNA^30,31^. Additional studies are needed to fully understand the transcriptional processing of *TRIB1* gene and how it may be regulated in different cancer types. To substantiate our observations that TRIB1 plays oncogenic roles in GBM, we performed conditional knockdown experiments using doxycycline-inducible *TRIB1*-shRNA as constitutive knockdown of *TRIB1* was found to be lethal in GBM cells. We found that *TRIB1* knockdown decreased the viability of T08-387 cells by 30% after 72 h of doxycycline induction (Supplementary Fig. 3a,b).

### Overexpression of TRIB1 compromises RT/TMZ-induced apoptosis

**Figure 3:**
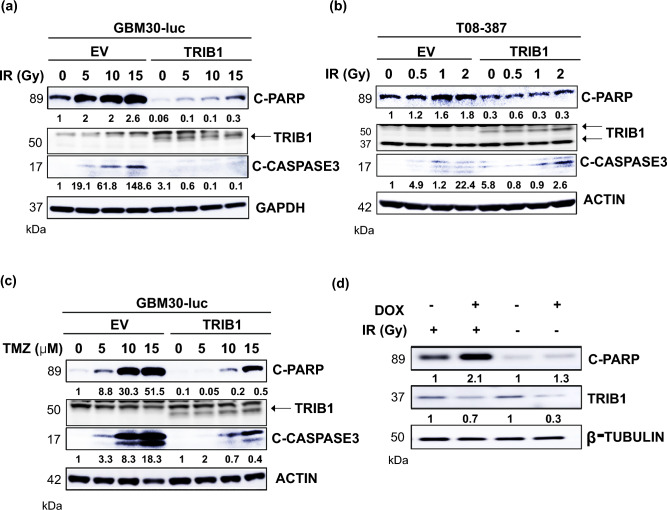
TRIB1 overexpression decreased RT induced apoptosis in GBM cells. Representative western blot shows cleaved PARP and cleaved caspase 3 after RT treatment in (**a**) GBM30-luc and (**b**) T08-387 cells and TMZ treatment in (**c**) GBM30-luc cells. (**d**) Representative western blot showing cleaved PARP after doxycycline induction (knockdown) of TRIB1 in T08-387 cells (clone 12). The comparison of band intensities was made between EV and TRIB1 with respect to different treatments (0-EV vs 0-TRIB1 etc.) using western blots from two different experiments. TRIB1 and PARP were probed on the same gel after cutting into half. C-Caspase 3 and loading control were probed on the same gel after cutting into half. For (**d**) all three proteins were probed on the same gel.

We further explored the mechanisms of RT/TMZ resistance in TRIB1 overexpressing GBM cells. The primary GBM cells were treated with the indicated doses of radiation and cultured for 48 h or with indicated concentrations of TMZ for 72 h and western blot was performed. We found that PARP cleavage in TRIB1-overexpressing GBM30-luc cells was reduced by an average of 2.5-fold after 15 Gy treatment compared with 15 Gy EV control. It was also accompanied by a decrease in caspase 3 cleavage (0.4 ± 0.05) (Fig. 3a). TMZ treatment also caused similar effects by decreasing PARP cleavage up to threefold (± 0.08) and caspase 3 cleavage up to threefold (± 0.05) in *TRIB1* transgene overexpressing cells (Fig. 3c). LN18 cells showed similar results with radiation treatment (Supplementary Fig. 4); however, T08-387-TRIB1 cells had decreased PARP cleavage (0.4 ± 0.07) after irradiation exposure but caspase 3 cleavage increased compared to the untreated control (Fig. 3b). Next, two conditional *TRIB1* knock down clones of T08-387 cells (clone 7 and 12) were chosen by antibiotic selection. *TRIB1* was knocked down in T08-387 clone 12 cells by doxycycline for 48 h followed by radiation treatment (2 Gy). The cells were collected 48 h after radiation treatment and an increase in RT-induced PARP cleavage (2.3 ± 0.2) was observed with a mean TRIB1 knockdown of threefold (0.67 ± 0.06) (Fig. 3d).

### TRIB1 regulates ERK- and Akt downstream signaling in GBM cells

**Figure 4:**
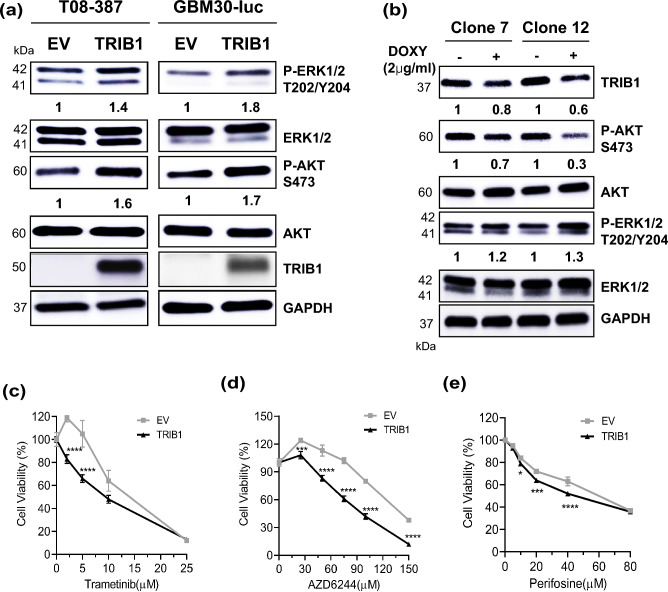
TRIB1 activates ERK and Akt signaling in GBM cells. (**a**) Representative western blot shows the change in phosphorylation of ERK and Akt after TRIB1 overexpression in T08-387 (left) and GBM30-luc (right) cells. (**b**) Representative western blots showing the change in phosphorylation of ERK and Akt after conditional knockdown of TRIB1 in T08-387 cells. Two different knockdown clones (number 7 and 12) were used. Line graphs depicting cell viability of T08-387 empty vector (EV) and TRIB1 overexpressing cells (TRIB1) after treatment with indicated concentrations of (**c**) Trametinib, (**d**) AZD6244 and (**e**) Perifosine for 72 h. Tukey's multiple comparison test was used to compare respective concentrations between EV and TRIB1 cells, n = 3; *p < 0.05; ***p < 0.001, ****p < 0.0001. n = 2 for all western blots. Akt and ERK proteins were probed on same blot after cutting. P-Akt and p-ERK were probed on the same blot after cutting. GAPDH was probed later on ERK and p-ERK blots. TRIB1 was probed on a separate blot.

To determine the contributions of TRIB1-activated ERK- and Akt signaling, the *TRIB1* transgene was overexpressed in GBM patient derived primary cell lines, which showed that phosphorylation/activation of ERK and Akt was increased by 1.4-fold (2.9 ± 0.2) and 1.6-fold (1.5 ± 0.2) respectively in T08-387 cells (Fig. 4a, left panel) and 1.8-fold (2 ± 0.03) and 1.7-fold (2.8 ± 0.08) in GBM30 cells (Fig. 4a, right panel) compared to empty vector control. The total ERK (1.1 ± 0.06) and Akt (1.2 ± 0.02) in empty vector and *TRIB1* transgene overexpressing cells remained largely unchanged (Fig. 4a). This was determined by comparing the ratio of phosphorylated and total protein between empty vector and TRIB1-overexpressing cells. We also compared the phosphorylation of Akt between immortalized normal human astrocytes (NHA-hTERT) and GBM patient derived primary cell lines. The patient derived cell lines had a higher Akt phosphorylation level compared to NHA-hTERT (9.3-fold in T08-387 and 7.6-fold in 3359 cells) (Supplementary Fig. 5a). After doxycycline induction, Akt phosphorylation was reduced threefold (± 0.006) in clone 7 cells and sevenfold (± 0.1) in clone 12 cells. The ERK phosphorylation remained largely similar in both clones (clone 7: 1.1 ± 0.08 and clone 12: 1.3 ± 0.15) (Fig. 4b). Total ERK and Akt levels were not significantly affected with doxycycline induction (clone 7 ERK: 1.1 ± 0.01, Akt: 0.8 ± 0.03; clone 12 ERK: 0.9 ± 0.02, Akt: 0.8 ± 0.15). MEK and Akt inhibitors were used to evaluate the contributions of ERK and Akt in the enhanced viability observed in TRIB1 overexpressing T08-387 cells. Cells were treated with trametinib (MEK1/2 inhibitor) and AZD6244 (MEK1 inhibitor) at indicated concentrations for 72 h and their viability was measured. A significant decrease in the viability of TRIB1 overexpressing cells was observed compared to empty vector, between 2.5 and 5 µM treatment of Trametinib (Fig. 4c) and between 30 and 150 µM concentrations of AZD6244 (Fig. 4d). Similarly, TRIB1-overexpressing cells treated with the Akt inhibitor perifosine also displayed significantly decreased cell viability starting at 10–40 µM concentrations (Fig. 4e). Collectively these data demonstrate that TRIB1 activates both MEK-ERK-, and Akt signaling in GBM cells in vitro.

### Mechanisms of ERK and Akt activation by TRIB1 pseudokinase

**Figure 5:**
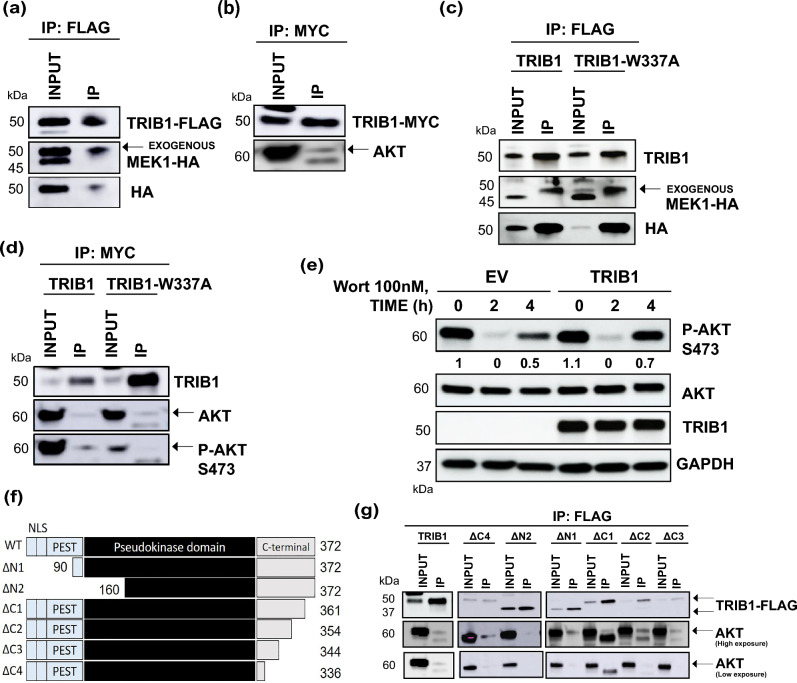
Mechanism of ERK and Akt activation by TRIB1. (**a**) Western blot showing MEK co-immunoprecipitation with TRIB1 in T08-387 cells. (**b**) Western blot showing Akt co-immunoprecipitation with TRIB1 in T08-387 cells. (**c**) Western blot showing MEK1 coimmunoprecipitation with TRIB1 and TRIB1-W337A in T08-387 cells. (**d**) Western blot showing Akt and p-Akt S473 coimmunoprecipitation with TRIB1 and TRIB1-W337A in T08-387 cells. (**e**) Western blot showing the change in Akt phosphorylation at S473 after wortmannin treatment in T08-387 empty vector and TRIB1 overexpression cells. The bands were quantitated with respect to 0 h in EV cells. (n = 3) (**f**) TRIB1 deletion mutant constructs. NLS- nuclear localization signal. (**g**) Western blot showing Akt coimmunoprecipitation with indicated deletion mutants of TRIB1 in HEK293 cells. n = 2 for all western blots except (**e**). For immunoprecipitation, each protein was probed on separate blots. For (**e**), Akt and p-Akt were probed on separate blots and GAPDH was similarly probed on respective blots after cutting. TRIB1 was probed on the same blot as Akt.

To investigate a possible interaction between TRIB1 and MEK in GBM cells, we stably overexpressed an HA-tagged MEK1 in T08-387 cells stably overexpressing FLAG-tagged TRIB1. We then co-immunoprecipitated (Co-IP) FLAG-tagged TRIB1 and probed with MEK and HA antibodies by western blot. The results revealed a TRIB1 and MEK1 interaction in T08-387 cells (Fig. 5a). We next performed Co-IP of Myc-tagged TRIB1 stably overexpressed in T08-387 cells and probed for Akt. The results showed TRIB1 also interacted with Akt in these cells (Fig. 5b). We then introduced a point mutation (Tryptophan (W) to Alanine (A) at amino acid 337) in the MEK binding motif (ILLHPW^20^) of TRIB1, and the subsequent Co-IP experiment showed that there was no disruption in TRIB1-MEK1 interaction with the mutant TRIB1 (Fig. 5c). We observed that Akt activation is decreased in these cells after mutating the MEK binding site on TRIB1 (Supplementary Fig. 5b). The co-immunoprecipitation results showed that *TRIB1*-W337A could bind to Akt but it had decreased phosphorylation (Fig. 5d). We also determined the contribution of PI3K in TRIB1-mediated Akt activation by treating TRIB1-overexpressing and empty vector control T08-387 cells with the PI3K inhibitor wortmannin at 100 nM concentration for indicated time points. The results revealed that Akt phosphorylation was decreased in both cell lines after 2 h of wortmannin treatment. However, after 4 h, Akt phosphorylation recovered to a higher level (3.6-fold ± 1.1) in TRIB1-overexpressing cells compared to empty vector cells (0.08-fold ± 0.03) (Fig. 5e). The total Akt protein levels were largely similar after wortmannin treatment with a mean of 1.3 ± 0.18 in empty vector cells and 0.9 ± 0.03 in TRIB1-overexpressing cells. These results suggest that heightened TRIB1 expression may counteract the effects of PI3K inhibition by promoting Akt phosphorylation. To determine the binding site of Akt on TRIB1, we utilized TRIB1 deletion mutants (Fig. 5f) lacking indicated residues at the N- and C terminus and within the pseudokinase domain. These expression plasmids were transiently transfected into HEK293 cells and co-immunoprecipitation (Co-IP) was performed. The results showed that Akt co-immunoprecipitated with all the deletion mutants except ΔN2, which narrows down the binding site to amino acids 90–160 (Fig. 5g).

### TRIB1 protein level oscillates during cell cycle and TRIB1 may cause p53 degradation through COP1

**Figure 6:**
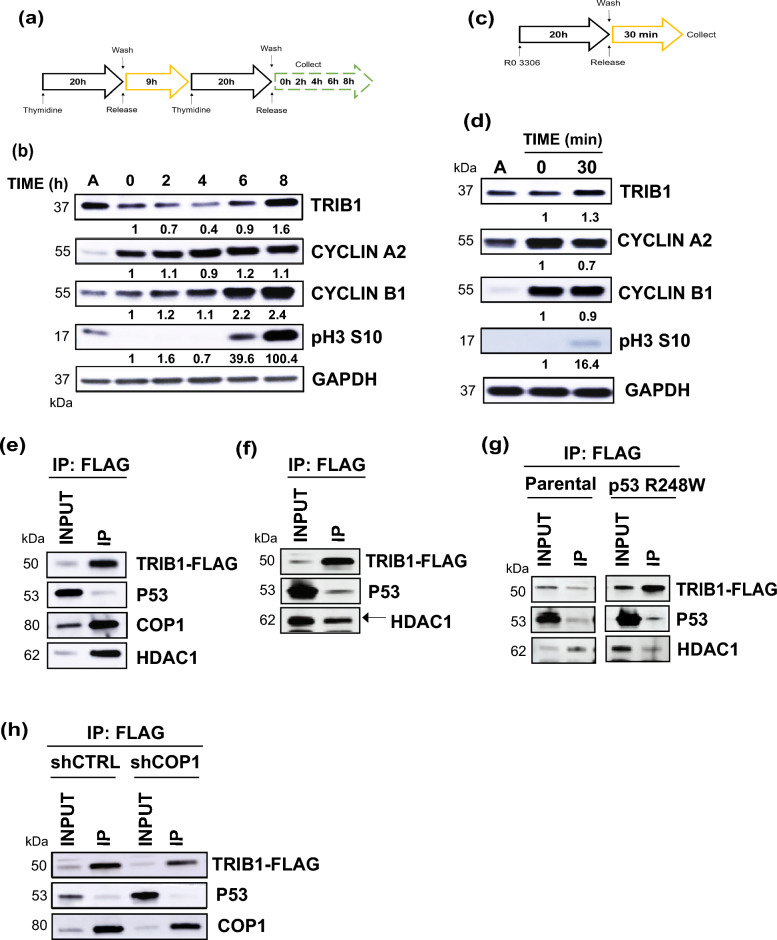
TRIB1 protein level oscillates during cell cycle and TRIB1 may cause p53 degradation through COP1. (**a**) Protocol schematic for double thymidine block in U87 MG cells. (**b**) Western blot depicting protein levels of TRIB1 and respective cell cycle proteins at indicated time points following thymidine release. (**c**) Protocol schematic for G2 arrest by Ro-3306 in U87 MG cells. (**d**) Western blot for protein levels of TRIB1 and respective cell cycle proteins 30 min after release from Ro-3306 treatment. Western blot showing the co-immunoprecipitation of TRIB1 with p53, COP1 and HDAC1 in (**e**) T08-387-TRIB1-FLAG cells and (**f**) GBM30-TRIB1-FLAG cells (n = 3). (**g**) Western blot showing the coimmunoprecipitation of TRIB1 with p53, COP1 and HDAC1 in 3359 parental and 3359-TP53 R248W cells after TRIB1 overexpression (n = 3). (**h**) Western blot showing co-immunoprecipitation of p53 and COP1 with TRIB1 in T08-387 cells after COP1 knockdown by shRNA. n = 2 for all western blots unless otherwise mentioned. For cell cycle experiment (**b**,**d**), depending on the molecular weight, one or more proteins were probed on same blot after cutting. For immunoprecipitation, each protein was probed on separate blots.

We observed that asynchronous cells exhibited variable TRIB1 protein levels across experiments, therefore we examined the dynamics of TRIB1 protein expression across different phases of the cell cycle. We performed double thymidine block in U87 MG GBM cells as shown in Fig. 6a and observed that TRIB1 levels peaked at 8 h after release (1.5 ± 0.1) at which point the cells were shown to be at G2/M phase in the cell cycle as revealed by flow cytometry (Supplementary Fig. 6c). The cell cycle proteins corresponding to G2- (cyclin B1 1.7 ± 0.7) and M phase (pH3 S10) also peaked during that time suggesting a G2/M transition (Fig. 6b). Cyclin A2 did not show much difference over the indicated time points. We also blocked U87 MG cells specifically at G2 phase using Ro-3306 (5 µM), a CDK1 inhibitor and collected cells after releasing for 30 min (Fig. 6c). The results showed that TRIB1 levels were maintained as cells exited the G2 phase (1.2 ± 0.1). The cell cycle phases were confirmed by the presence of cyclin A2, cyclin B1 and histone H3 phosphorylation at Ser10 by western blotting (Fig. 6d). The same cell cycle proteins were also checked in the asynchronous cell population. The phases of cell cycle were monitored by flow cytometry (Supplementary Fig. 6c,d) and cell cycle proteins corresponding to other phases of the cell cycle were also checked (Supplementary Fig. 6a,b). Since p53 is shown to be inactivated by TRIB1 in NSCLC and HCC, we next explored the interaction between TRIB1, p53 and COP1 in T08-387 cells that contain wild-type *TP53*. A co-immunoprecipitation of TRIB1 was performed, in T08-387-*TRIB1*-FLAG stable cells. The results showed that COP1 and p53 co-immunoprecipitated with TRIB1 (Fig. 6e). P53 similarly immunoprecipitated with TRIB1 in LN18-*TRIB1*-FLAG (Supplementary Fig. 6f), GBM30-*TRIB1*-FLAG (Fig. 6f) and, in isogenic 3359-Parental and 3359-*TP53* R248W cells (Fig. 6g). We also observed that HDAC1, a repressor of p53 mediated transcription also co-immunoprecipitated with TRIB1 (Fig. 6e–g and Supplementary Fig. 6f). Furthermore, upon COP1 knockdown (Supplementary Fig. 6e), p53 binding to TRIB1 decreased by about 45 percent (± 0.09) (Fig. 6h). Taken together our data showed that the TRIB1 protein level peaks during G2-phase of the cell cycle and may inhibit p53 function by associating with HDAC1 and facilitating degradation through COP1.

### TRIB1 overexpression decreased OS of tumor-bearing mice

**Figure 7:**
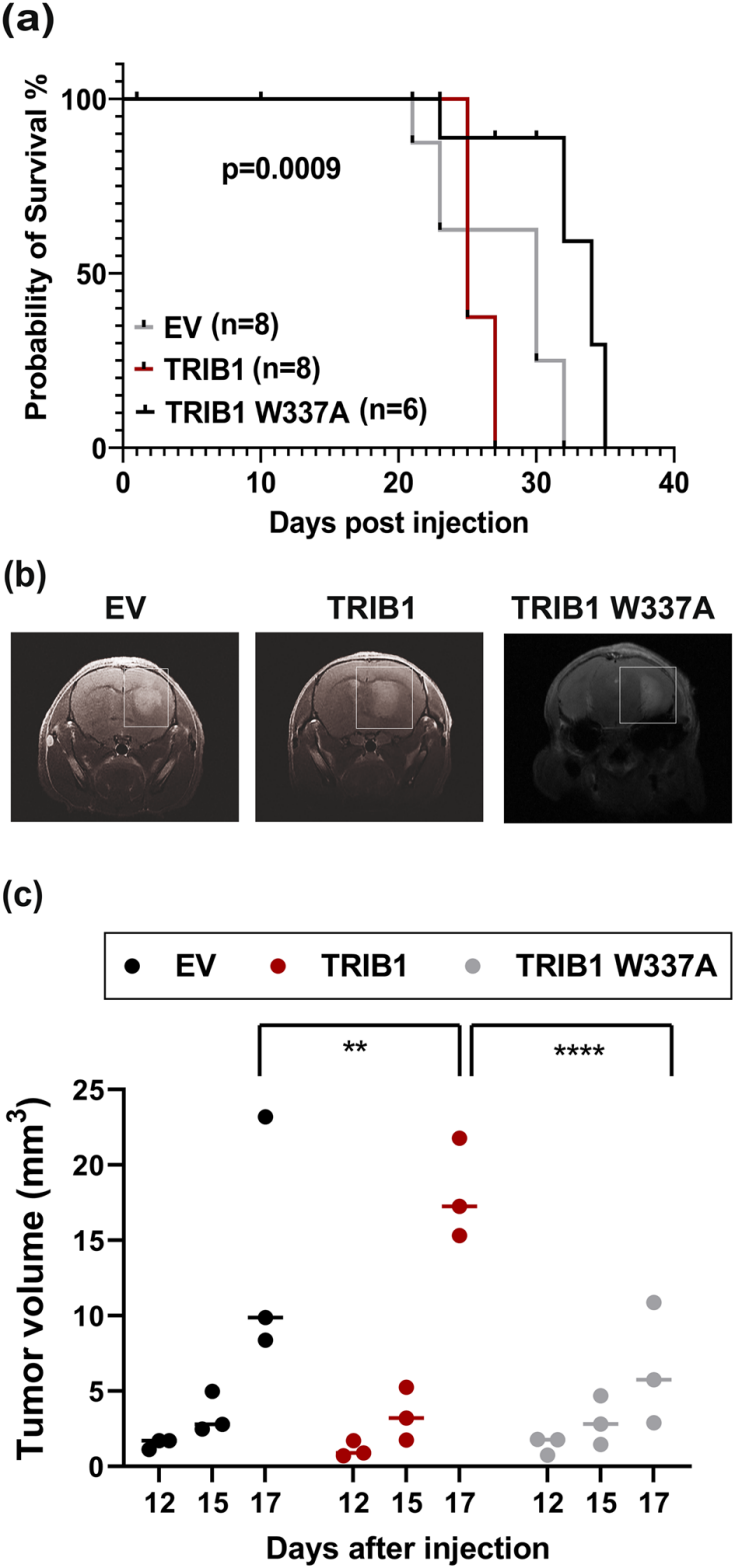
TRIB1 decreases OS in nude mice. (**a**) Kaplan–Meier survival curve shows OS of mice from EV (n = 8), TRIB1 (n = 8) and TRIB1-W337A groups (n = 6). (**b**) Representative MRI images of the tumor from the indicated groups taken at day 17 post injection. Tumors are outlined by white boxes. (**c**) Tumor volumes from indicated groups (n = 3 per group). Tumors were imaged by MRI on indicated days post injection and tumor volume was calculated by ITK SNAP software. Tukey's multiple comparison test was performed to compare the mean of day 17 tumor volumes between EV, TRIB1 and TRIB1 W337A **p < 0.01, ****p < 0.0001.

To evaluate the tumorigenic potential of TRIB1 in vivo, we used an orthotopic mouse model of human GBM xenografts. We performed intracranial injection of mice with T08-387 empty vector cells, T08-387-*TRIB1*-FLAG cells or T08-387-*TRIB1*-W337A-FLAG cells to generate tumors. We observed that mice with TRIB1 overexpressing tumors had lower OS than the empty vector and TRIB1-W337A groups (Fig. 7a). Mice bearing TRIB1-W337A tumors had the highest OS. Three mice from each group were subjected to MRI imaging at indicated days after injection. Tumors with wild type TRIB1 overexpression had higher tumor volume at day 17 post injection compared to EV or TRIB1-W337A tumors (Fig. 7b,c). We also observed that one of the empty vector tumors had increased tumor volume comparable to TRIB1 tumors therefore it was excluded from significance testing. Thus, this in vivo experiment showed that TRIB1 overexpression enhanced tumor growth, which involved TRIB1-MEK1 interaction, suggesting that TRIB1 may serve as a druggable target in GBM. To this end, discovery and development of small molecule inhibitors targeting TRIB1 are in progress in the laboratory.

## Discussion

Since the adoption of the Stupp protocol for treatment of GBM in 2005, a multitude of studies have been performed for the improvement of GBM therapy^32^. So far Tumor Treating Fields (TTF) remains the only additional therapy that has been approved by FDA for the treatment of recurrent as well as newly diagnosed GBM^33^. All these therapies follow the premise of increasing cell death by broad mechanisms^34,35^ which are eventually counteracted by resistance mechanisms thereby significantly compromising OS. Taking newer insights into consideration that GBM is a clinically diverse disease and that each GBM patient might have a different disease etiology, there is an urgent need to identify new therapeutic targets and subsequently develop targeted therapies that would improve or complement the current treatments thus overcoming resistance mechanisms. To this end, our correlative studies identified a number of therapeutically vulnerable protein targets in GBM, which include TRIB1 among others. Herein, we report the in vitro and in vivo validation of TRIB1 as a potential therapeutic target in preclinical GBM models.

Previous reports have indicated that TRIB1 is overexpressed in several cancer types including prostate cancer, lymphoma and, CNS cancers^22^. Our analysis of institutional and TCGA GBM and LGG cohorts showed that increased *TRIB1* gene expression was associated with lower OS of GBM patients and was also an independent predictor of overall survival. *TRIB1* gene expression was also found to be upregulated particularly in *IDH1/2* wild-type gliomas, who generally have worse prognosis than their *IDH1/2* mutant counterparts^36^. Our in vivo studies showed that mice bearing TRIB1 overexpressing tumors generated from patient derived primary GBM cell lines had increased tumor volume and shorter OS compared to mice with empty vector control tumors. Moreover, we found that TRIB1 protein levels were lower in immortalized normal human astrocytes compared to patient derived primary GBM cell lines. These observations prompted us to validate the role of TRIB1 in GBM tumor progression and therapy resistance. We observed that RT/TMZ treatments increased TRIB1 mRNA and protein levels in a GBM cell line that harbors a *TP53* loss of function mutation. We then stably overexpressed TRIB1 in this as well as two patient derived primary cell lines. We observed increased viability of these cell lines in response to RT/TMZ treatments accompanied by reduced PARP and caspase 3 cleavage. We also observed that TRIB1 knockdown led to increased PARP cleavage after RT treatment suggesting that TRIB1 increased cell viability in these cell lines by reducing apoptosis induced by RT/TMZ treatments. It is noteworthy that TRIB1 expression was required for the survival of GBM cells as RNAi-mediated constitutive knockdown of TRIB1 expression was lethal. Furthermore, CRISPR knockout did not provide viable stable clones of GBM cell lines suggesting that TRIB1 is required for their survival.

There are numerous pathways known in cancer cells to induce anti-apoptotic effects by various mechanisms^37^. TRIBs have been previously reported to activate the ERK signaling pathway by MEK binding which is known to promote cell proliferation in various cancer types^38^. Previous reports have also shown that TRIB2 and TRIB3 activate Akt in various cancer cells by direct binding resulting in therapy resistance ^39^ and cancer cell stemness^40^ respectively. In addition to Akt phosphorylation, TRIB3 can also cause Akt dephosphorylation in liver under fasting conditions^41^. Akt is a downstream mediator of the PI3K pathway that is centrally involved in a myriad of cellular processes like survival, growth, metabolism and proliferation^42^. It is often found to be aberrantly activated in cancers and is also implicated in therapy resistance^43^. Akt is activated in ~ 90% of GBM cases linked to activation of PI3K pathway^44^. Immortalized normal human astrocytes had low Akt phosphorylation compared to patient derived primary cell lines. Therefore, we sought to determine the impact of TRIB1 overexpression on ERK and Akt pathways in GBM cell lines and found that TRIB1 overexpression increased the phosphorylation of ERK and Akt suggesting the activation of survival pathways by TRIB1. A similar effect was previously observed in melanoma tumor tissues where high TRIB2 expression correlated with increased Akt phosphorylation^39^. However, upon TRIB1 knockdown Akt phosphorylation but not ERK1/2 phosphorylation was reduced. A similar observation was made in breast cancer cells where knockdown of TRIB1 led to the inhibition of Akt phosphorylation^45^. Therefore, it can be concluded that Akt activation in GBM cells may be highly dependent upon TRIB1 whereas ERK activation could be compensated for by other signaling components. This observation prompted us to get a deeper insight of how the MEK-ERK pathway contributes to TRIB1-mediated oncogenic signaling in GBM. To address this, we performed co-immunoprecipitation to evaluate TRIB1 interaction with MEK as well as Akt. Our results showed that both MEK and Akt were co-immunoprecipitated with TRIB1.

A previous study in AML showed that the tryptophan (W) residue in the ILLHPW (residues 332-338) motif of TRIB1 is required for MEK binding^20^. Therefore, we utilized a W to A mutation to evaluate MEK binding to TRIB1. This mutation did not abolish the TRIB1-MEK interaction in GBM cells possibly indicating a cell-type specific effect. In addition, Akt activation was decreased in these cell lines after introducing this mutation. This suggests that mutating the MEK binding site on TRIB1 has indirect effects on its downstream signaling. Furthermore, our in vivo results showed that mice bearing TRIB1-W337A tumors had lower tumor volumes and longer OS compared to those with wild type TRIB1 overexpressing tumors and empty vector control tumors. We also observed that TRIB1-W337A protein expression was lost in stable cell lines after a few passages. These observations suggest that TRIB1-MEK signaling might play an important role in GBM tumor growth. To explore this further, we utilized MEK inhibitors to check the effect of MEK inhibition on GBM cells. The viability of cells overexpressing TRIB1 was affected to a greater extent in terms of cell viability than empty vector control cells suggesting the dependence of TRIB1 overexpression cells on the MEK-ERK signaling pathway for survival.

This is the first report, to our knowledge, showing that Akt binds to TRIB1. We also determined that residues 90–160 on TRIB1 are important for Akt binding, which is different from the Akt binding site on TRIB2^39^ and TRIB3 proteins^40^. Interestingly, mutation in the MEK binding site at TRIB1 did not disrupt the TRIB1-MEK interaction but reduced Akt phosphorylation in these cells, revealing a new role associated with this binding site in GBM cells. In addition, we inhibited PI3K in GBM cells by wortmannin and observed that Akt phosphorylation was restored to a greater level in TRIB1 overexpression cells suggesting that TRIB1 may confer resistance to P13K inhibitory therapies. Previous reports have shown a similar effect where TRIB2 overexpression was able to counteract the effects of PI3K inhibition in vivo by displaying increased Akt phosphorylation after treatment^39^. The viability of TRIB1 overexpressing cells was reduced by Akt inhibition, indicating the dependence of GBM cells on the Akt pathway for survival. The above results support that TRIB1 increases survival of GBM cells by upregulating ERK and Akt signaling in these cells.

Sustained proliferation is the most fundamental trait of a cancer cell^37^. Several mechanisms have been reported across different tumors which deregulate the cell cycle in these cells. In GBM, this is achieved by a myriad of mutations that affect multiple cellular pathways resulting in tumor formation^46^. In this study, we showed that TRIB1 increased GBM cell survival through ERK and Akt, which are central regulators of pathways involved in tumor maintenance and growth of GBM cells. TRIB1 was identified as a novel regulator of G1/S transition in breast cancer cells using cell cycle arrest models made by MEK1/2 inhibition in different cell lines^45^. Therefore, we wanted to determine the expression profile of TRIB1 protein across the phases of cell cycle in GBM cells. We observed that TRIB1 protein levels were highest in the G2/M phase of the cell cycle. It is known that G2/M checkpoint is activated in response to radiation induced DNA damage^47^. One of the events that happens during this complex process is the degradation of cdc25c phosphatases, which leads to G2/M arrest^48^. It has been shown that TRIB2 selectively targets the mitotic cdc25c for degradation possibly through ERK^49^. Therefore, based on our results it can be concluded that TRIB1 might play a role in inducing G2/M arrest in GBM cells in response to radiation treatment thus leading to increased cell survival. Additional studies are needed to further validate the role of TRIB1 in GBM cell cycle.

*TP53* is a well-known tumor suppressor gene in almost all human malignancies. It is known as the guardian of the genome and is mutated in approximately 42 percent of all cancer types^50^. In GBM, the p53 pathway is deregulated in about 85 percent of patients and is involved in invasion, migration, proliferation and apoptosis resistance^51^. It has been shown that p53 is targeted for degradation by the COP1 E3 ubiquitin ligase in GBM cells^52^. Since COP1 is also a binding partner of TRIB1^53^, we hypothesized that TRIB1 is required for p53 and COP1 interaction. Our results showed that both p53 and COP1 were co-immunoprecipitated with TRIB1. However, p53 pull down was decreased after COP1 knockdown even though the total p53 protein levels were increased. These results indicate that TRIB1 might act as a scaffold for COP1 mediated p53 degradation. Another mechanism by which TRIB1 regulates p53 is through HDAC1. TRIB1 associates with HDAC1 which represses transcriptional activity of p53 by deacetylation and also decreases its DNA binding^54,55^. We observed that HDAC1 co-immunoprecipitated with TRIB1 along with p53 in different patient derived primary GBM cell lines independent of their p53 mutation status (Supplementary Table 4). All the above results indicate that TRIB1 may modulate p53 function in GBM cells by multiple mechanisms.

In summary, our study indicates that TRIB1 increased survival of GBM cells by conferring resistance to RT/TMZ therapies through the upregulation of ERK and Akt oncogenic signaling pathways. TRIB1 could also manipulate the cell cycle through these pathways and render p53 ineffective by guiding its degradation and functional disruption. Altogether these observations suggest that TRIB1 is a potential candidate for designing targeted therapies in GBM. Some limitations of our study include the use of only three cohorts for the correlation studies of TRIB1 mRNA levels with patient OS. Validation in additional cohorts is necessary to better capture the intra-tumoral heterogeneity associated with GBM. Second, a previous report showed that the W residue in TRIB1 is required for MEK binding to TRIB1, however this interaction was not disrupted in GBM cells after mutating W to A. It was observed to cause other effects instead. Finally, additional studies are required to further validate the role of TRIB1 in p53 modulation, and cell cycle. Our future studies aim at addressing some of the limitations highlighted above and designing small molecule inhibitors targeting and disrupting, TRIB1 interactions with MEK1/2, Akt, COP1 and C/EBPα and then testing their efficacy using in vivo preclinical mouse models.

## Supplementary Information

Supplementary Information.

## Data Availability

Data from our institutional cohorts has been provided in the supplementary file. Other data is already publicly available (https://www.cancer.gov/tcga).
