# Supplementary material for: TRIB1 confers therapeutic resistance in GBM cells by activating the ERK and Akt pathways
Source: Sci Rep. 2023 Aug 1;13:12424. doi: 10.1038/s41598-023-32983-w (PMC10394028; doi:10.1038/s41598-023-32983-w)
Supplement: Supplementary file 1 — Supplementary Information. [file 41598_2023_32983_MOESM1_ESM.pdf]

TRIB1 confers therapeutic resistance in GBM cells by activating the ERK and Akt pathways

Karnika Singh[1], Chunhua Han[1], Jessica L. Fleming[1], Aline P. Becker[1], Joseph McElroy[2], Tiantian Cui[3], Benjamin Johnson[1], Ashok Kumar[1], Ebin Sebastian[4], Christian A. Showalter[1], Morgan S. Schrock[1], Matthew K. Summers[1], Valesio Becker[1], Zhen-yue Tong[1], Xiaomei Meng[1], Heather R. Manring[1], Monica Venere[1], Erica H. Bell[5], Pierre A. Robe[6], A. L. Grosu[7], S. Jaharul Haque[1], Arnab Chakravarti[1]*

[1]Department of Radiation Oncology, The Ohio State University Comprehensive Cancer Center, Columbus, Ohio 43210, USA

[2]The Ohio State University Center for Biostatistics, Department of Biomedical Informatics, Columbus, Ohio 43210, USA

[3]Department of Radiation Oncology, City of Hope, Duarte, California 91010, USA

[4]Corwell Health William Beaumont University Hospital, Royal Oak- Radiation Oncology, Michigan 48073, USA

[5]Neroscience Research Institute/ Department of Neurology, The Ohio State University Comprehensive Cancer Center, Columbus, OH, 43210, USA

[6]Department of Neurology and Neurosurgery, Brain Center Rudolf Magnus, University Medical Center Utrecht, Utrecht 3584 CG, Netherlands

[7]Freiburg University, Freiburg 79098, Germany

*Corresponding author

SUPPLEMENTARY METHODS

1. TRIB1 custom sequence

AAAC **ATG** CGG GTC GGT CCG GTG CGC TCT GCC ATG AGC GGC GCC TCG CAG CCC CGC GGC
CCG GCC CTG CTC TTC CCA GCC ACC CGA GGC GTC CCG GCC AAA CGC CTG CTG GAC GCC
GAC GAC GCG GCG GCT GTG GCG GCC AAG TGC CCG CGC CTC TCC GAG TGC TCC AGC CCC
CCG GAC TAC CTC AGC CCC CCC GGC TCG CCC TGC AGC CCG CAG CCC CCG CCT GCC GCT
CCG GGG GCC GGC GGA GGC TCC GGG AGC GCG CCG GGG CCC AGC CGC ATC GCC GAC TAC
CTG CTG CTG CCC CTA GCC GAG CGC GAG CAT GTG TCC CGG GCG CTG TGC ATC CAC ACT
GGA CGC GAG CTG CGC TGC AAG GTG TTT CCC ATT AAA CAC TAC CAG GAC AAA ATC AGG
CCT TAC ATC CAG CTG CCA TCG CAC AGC AAC ATT ACT GGC ATT GTG GAA GTG ATC CTT
GGG GAA ACC AAG GCC TAT GTC TTC TTT GAG AAG GAC TTT GGG GAC ATG CAC TCC TAT
GTG CGA AGC CGG AAG AGG CTG CGG GAA GAG GAA GCC GCC CGG CTC TTC AAG CAG ATT
GTC TCC GCC GTC GCC CAC TGC CAC CAG TCA GCC ATC GTG CTG GGG GAC CTG AAG CTT
AGG AAG TTC GTC TTC TCC ACG GAG GAG AGA ACC CAG CTT AGA CTA GAA AGT CTA GAA
GAC ACA CAC ATA ATG AAG GGG GAA GAT GAT GCT TTG TCA GAC AAA CAT GGC TGC CCA
GCC TAC GTG AGC CCT GAG ATC CTC AAC ACC ACT GGG ACC TAC TCC GGA AAG GCT GCG
GAC GTT TGG AGC CTG GGG GTG ATG CTC TAC ACC CTT CTG GTT GGA CGA TAC CCC TTC
CAT GAC TCA GAC CCC AGT GCC CTT TTC TCC AAA ATT CGG CGT GGA CAG TTC TGC ATT
CCT GAG CAC ATT TCC CCC AAA GCC AGG TGC CTC ATT CGC AGC CTC TTG AGA CGG GAG
CCC TCC GAG AGA CTC ACT GCC CCC GAG ATC CTA CTG CAC CCC TGG TTT GAG TCC GTC
TTG GAA CCC GGG TAC ATC GAC TCA GAA ATA GGA ACT TCA GAC CAG ATT GTT CCA GAG
TAC CAG GAG GAC AGT GAC ATT AGT TCC TTC TTC TGC **GGT GGC** **GAA CAA AAA CTC ATC**
TCA GAA GAG GAT CTG **TAA**

Kozak Sequence

Start codon

TRIB1 ORF

Glycine link

Myc-Tag

Stop codon

2. Sequence of custom primers for generating TRIB1-W337A:

Forward: 5'-gatcctactgcaccccgcgtttgagtccgtcttg-3’

Reverse: 3'-ctaggatgacgtggggcgcaaactcaggcagaac-5’

Supplementary Table 1. Patient demographics split by three cohorts

	level	Freiburg_LGG	TCGA_GBM	TCGA_LGG
n		35	529	513
Age_Years (mean (SD))		45.26 (13.93)	57.68 (14.57)	43.09 (13.47)
Sex (%)	female	11 (31.4)	205 (38.8)	200 (39.0)
	male	24 (68.6)	320 (60.5)	254 (49.5)
	NA	0 (0.0)	4 (0.8)	59 (11.5)
KPS.CODE (%)	<70	3 (8.6)	95 (18.0)	16 (3.1)
	>=70	32 (91.4)	297 (56.1)	243 (47.4)
	NA	0 (0.0)	137 (25.9)	254 (49.5)
IDH_Status.CODE (%)	mutant	29 (82.9)	34 (6.4)	417 (81.3)
	wild-type	6 (17.1)	380 (71.8)	93 (18.1)
	NA	0 (0.0)	115 (21.7)	3 (0.6)

Supplementary Table 2. List of primary antibodies

Name	Company	Catalog #	Dilution	Application
TRIB1	Abclonal	A10134	1:1000	Western Blotting
Cleaved-PARP	Cell Signaling Technology	5625	1:1000	Western Blotting
Cleaved-caspase 3	Cell Signaling Technology	9661	1:500	Western Blotting
ERK1/2	Cell Signaling Technology	9102	1:2000	Western Blotting
P-ERK1/2	Cell Signaling Technology	9101	1:2000	Western Blotting
MEK1/2	Cell Signaling Technology	9122	1:1000	Western Blotting
Akt	Cell Signaling Technology	9272	1:2000	Western Blotting
p-Akt S473	Cell Signaling Technology	4060	1:2000	Western Blotting
HA tag	Cell Signaling Technology	3724	1:1000	Western Blotting
Cell Cycle Phase Determination Antibody Sampler Kit	Cell Signaling Technology	17498	1:1000	Western Blotting
P53 (DO-1)	Santa Cruz	sc-126	1:1000	Western Blotting
COP1	Abcam	ab56400	1:1000	Western Blotting
HDAC1	Cell Signaling Technology	5356	1:1000	Western Blotting
P21	Cell Signaling Technology	2947	1:1000	Western Blotting
MDM2	Cell Signaling Technology	86934	1:1000	Western Blotting
GAPDH	Cell Signaling Technology	5174	1:2000	Western Blotting
β-Tubulin	DSHB	AB_2315513	1:2000	Western Blotting
β-Actin	Cell Signaling Technology	4967	1:1000	Western Blotting

Supplementary Table 3. List of secondary antibodies

Name	Company	Catalog #	Dilution	Application
Anti-mouse IgG, HRP-linked Antibody	Cell Signaling Technology	7076	1:2000	Western Blotting
Anti-rabbit IgG, HRP-linked Antibody	Cell Signaling Technology	7074	1:2000	Western Blotting

Supplementary Table 4. The p53 status of indicated cell lines

	Cell line	P53 status
1	LN18	Mutated
2	U87 MG	Wild Type
3	T08-387	Wild Type
4	GBM30	Mutated
5	GBM3359	Wild Type

SUPPLEMENTARY FIGURE 1

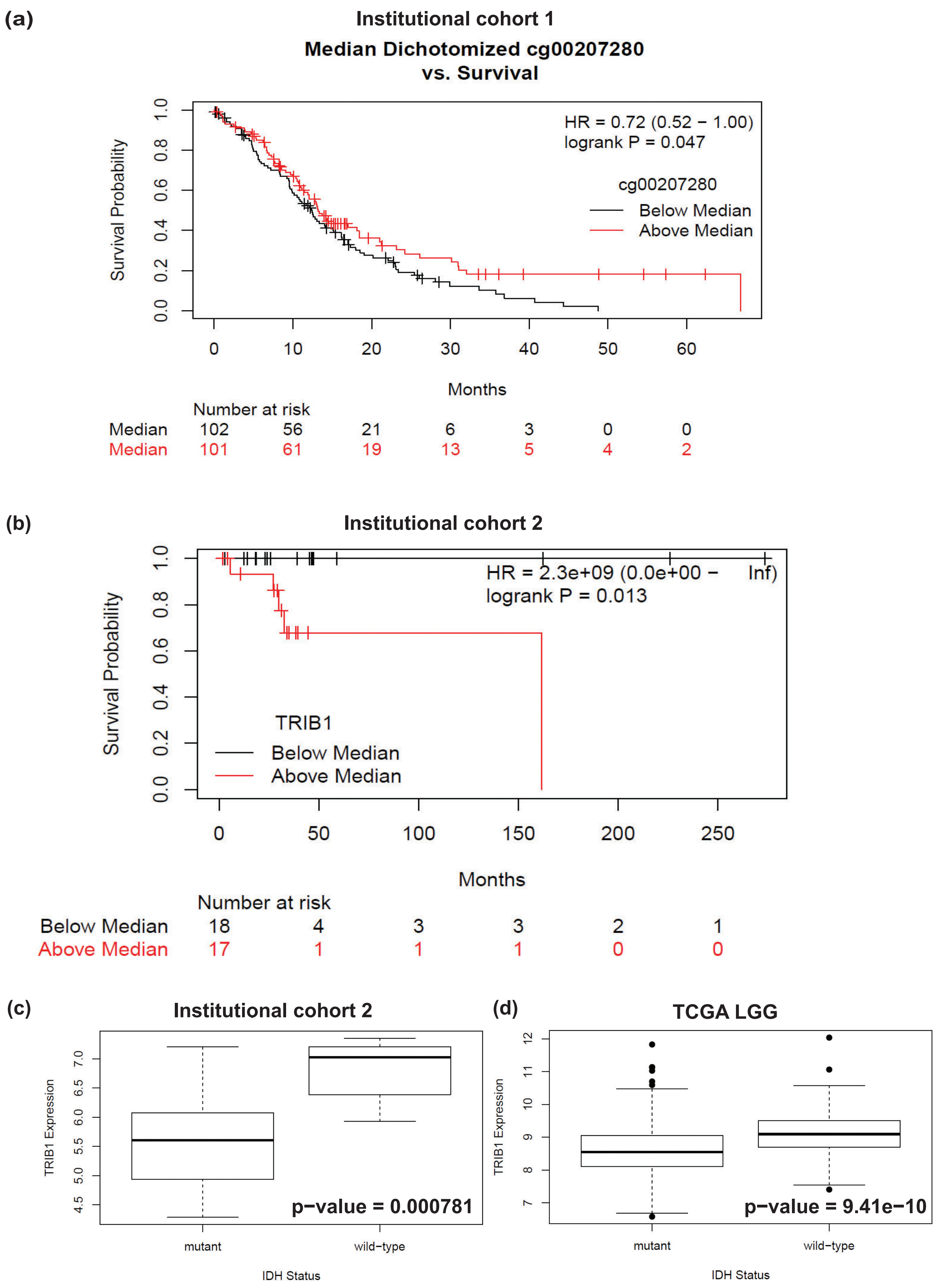

Supplementary Figure 1. Correlation of TRIB1 promoter methylation and mRNA overexpression with OS of patients. (a) Kaplan-Meier curve shows the correlation between TRIB1 promoter methylation corresponding to cg00207280 probe with OS in institutional cohort 1. (b) Kaplan Meier curve shows the correlation of *TRIB1* expression with survival of patients in institutional cohort 2. Box plot depicting *TRIB1* expression levels in IDH mutant and IDH wild-type patients in (c) institutional cohort 2 and (d) TCGA LGG cohort.

SUPPLEMENTARY FIGURE 2

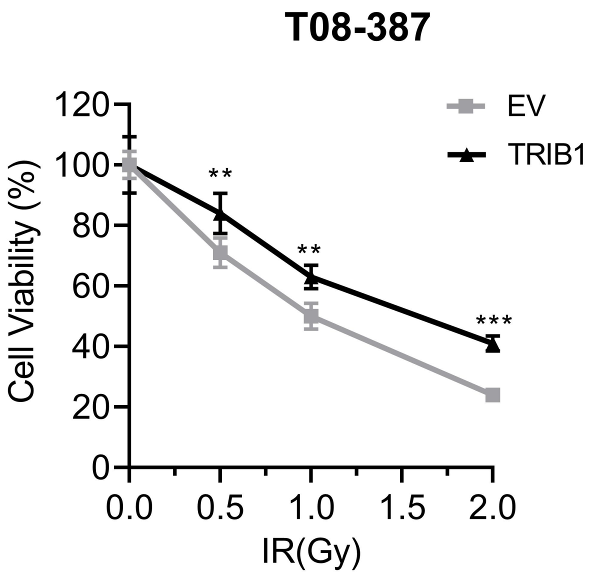

Supplementary Figure 2. TRIB1 protein overexpression increases cell viability. Line graph shows the cell viability of T08-387 empty vector (EV) and TRIB1 overexpressing cells (TRIB1) after RT. n=3

SUPPLEMENTARY FIGURE 3

(a)

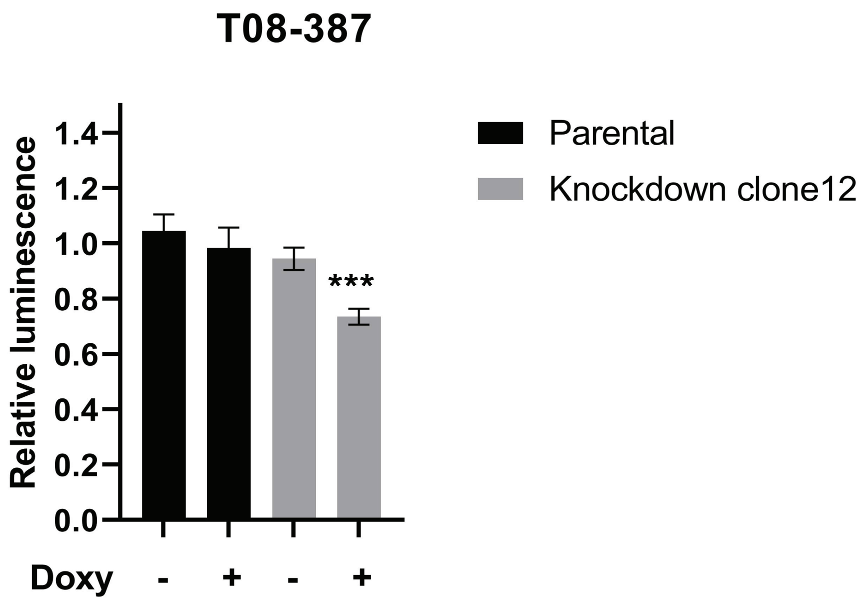

(b)

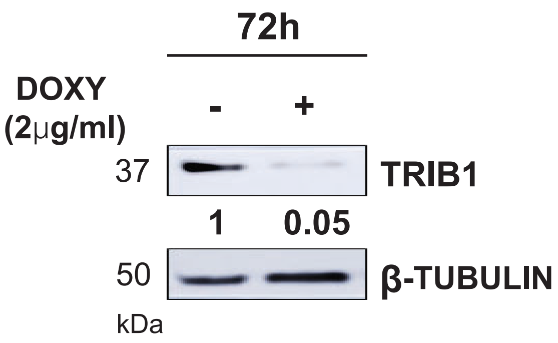

Supplementary Figure 3. TRIB1 knockdown decreases cell viability of T08-387 cells. (a) Bar graph shows the cell viability of T08-387 cells before and after TRIB1 knockdown induced by doxycycline treatment in knockdown clone 12. (b) Representative western blot shows TRIB1 knockdown in clone 12 after 72 hours of doxycycline treatment. n=3 Both proteins were probed on the same blot after cutting.

SUPPLEMENTARY FIGURE 4

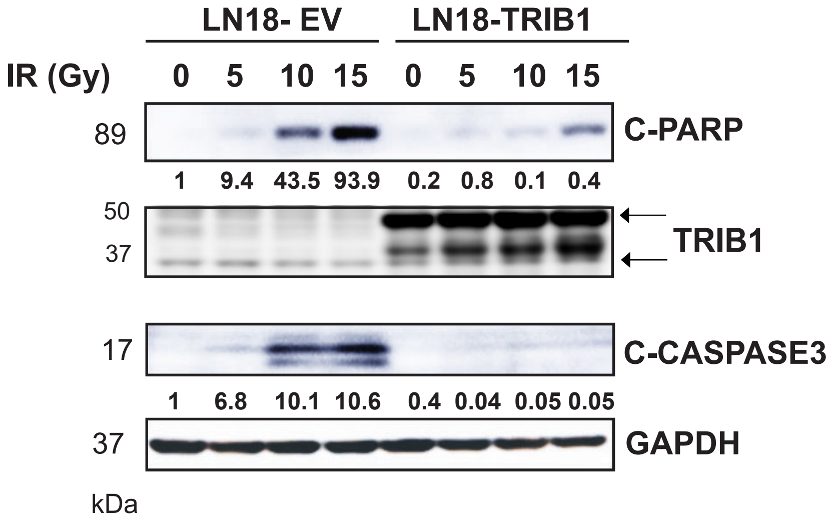

Supplementary Figure 4. TRIB1 protein overexpression decreases radiation induced apoptosis in LN18 cells. Western blot shows the protein levels of cleaved PARP, TRIB1 and cleaved caspase 3 after RT treatment in LN18 empty vector (LN18-EV) and TRIB1 overexpression (LN18-TRIB1) cells. n=1 The proteins were probed similar to Figure 3.

SUPPLEMENTARY FIGURE 5

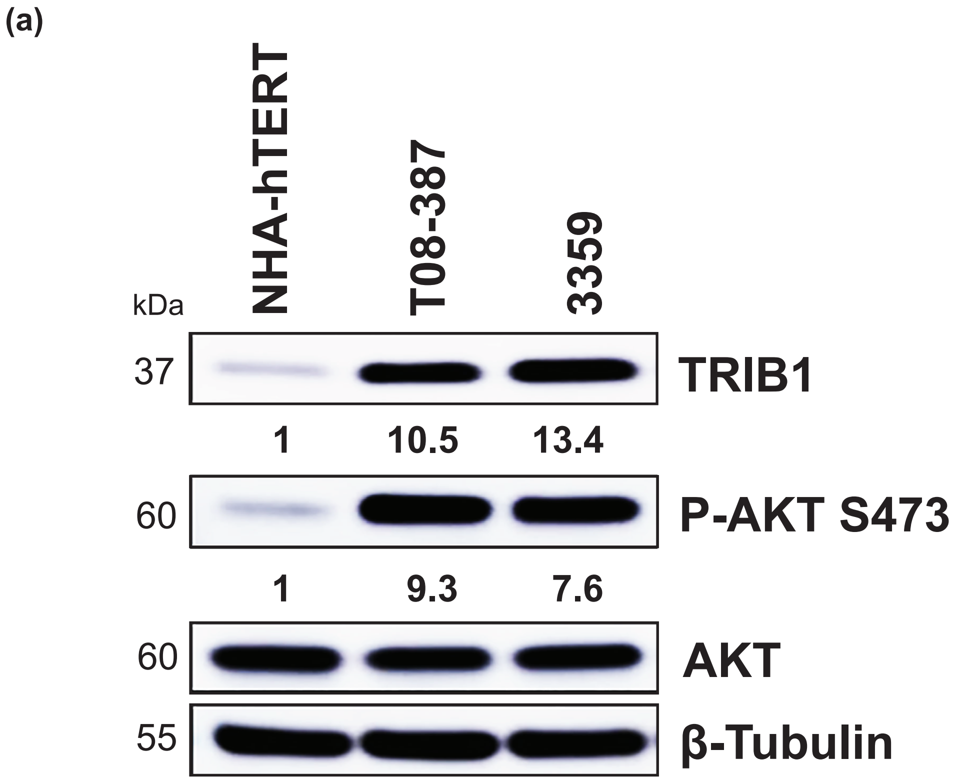

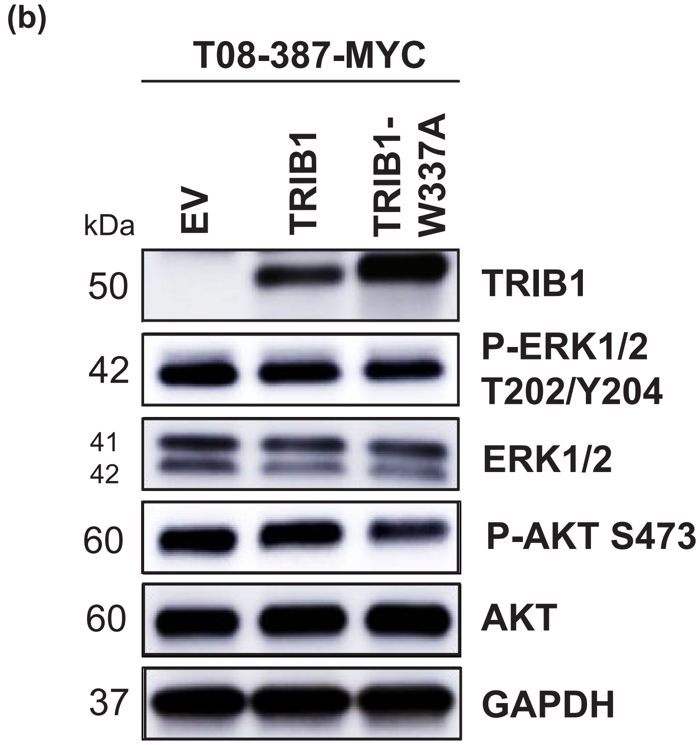

Supplementary Figure 5. Potential mechanism of Akt activation by TRIB1. (a) Representative western blot shows TRIB1, Akt and p-Akt S473 levels in immortalized normal human astrocytes (NHA-hTERT) and the indicated patient derived cell lines (T08-387 and 3359). Akt and p-akt were probed on separate blots. TRIB1 and beta-tubulin were probed on same blot after cutting. (b) Representative western blot shows the change in phosphorylation of ERK and Akt after TRIB1 and TRIB1-W337A overexpression in T08-387 cells. n=2 Proteins were probed similarly as figure 4a.

SUPPLEMENTARY FIGURE 6

(a)

Marker	Cell cycle phase
pH3 S10	Mitosis
Cdt1	G1 and S
Cyclin A2	G2
Cyclin B1	Late G2/M
Cyclin E1	Late G1/early S

(b)

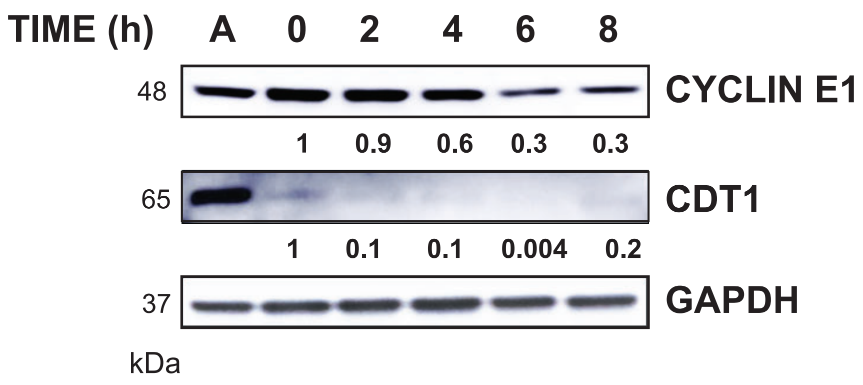

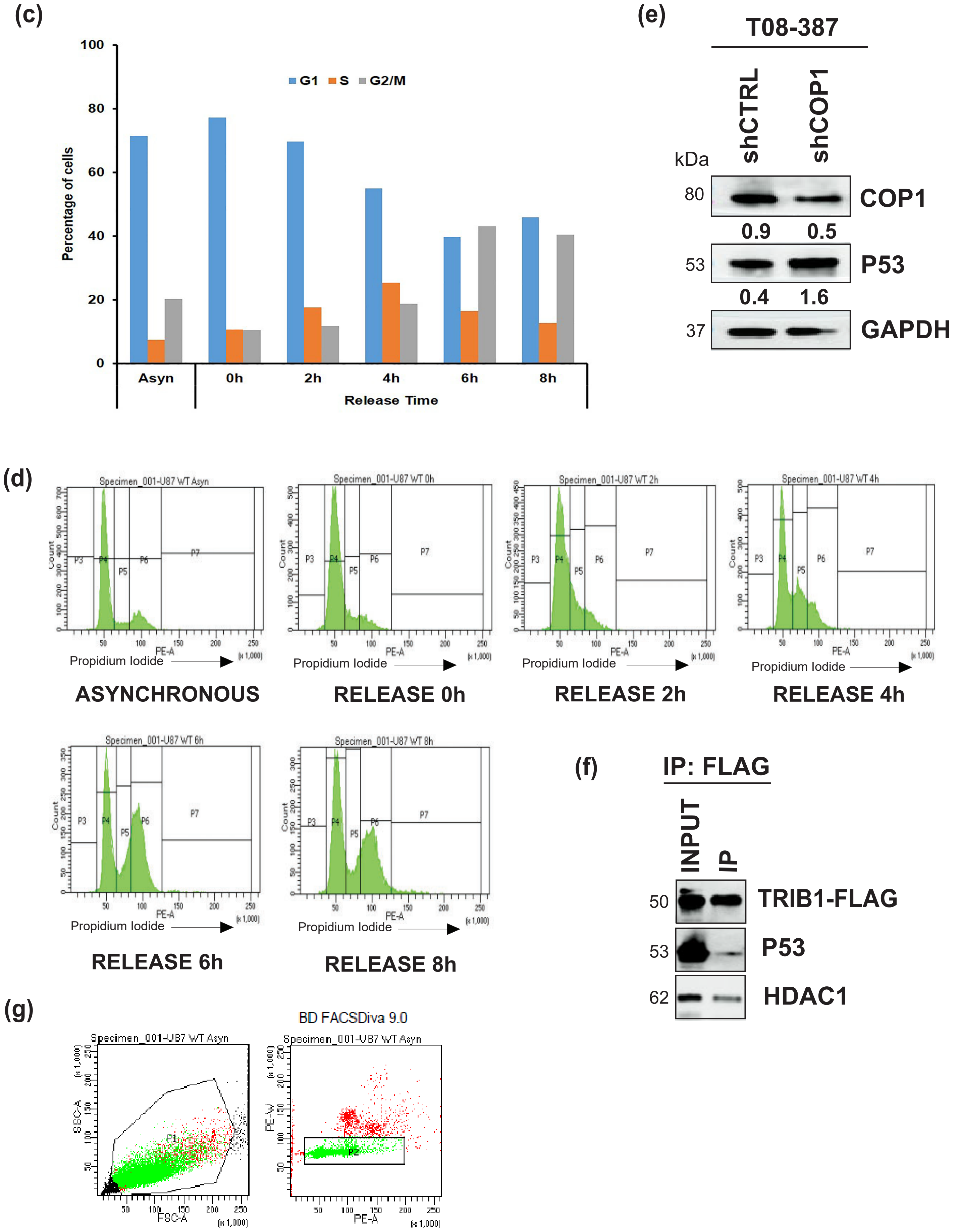

Supplementary Figure 6. Levels of different cell cycle proteins during various stages of cell cycle. (a) Table shows cell cycle proteins that act as markers of respective cell cycle phases. (b) Representative western blot shows protein levels of indicated cell cycle proteins detected after release from double thymidine block in U87 MG cells. 'A' represents asynchronous cells. n=2 Western blots were probed similar to figure 6(c). (c) Bar graph depicts the quantitation of flow cytometry analysis measured as percentage of cells in each phase of the cell cycle after release from double thymidine block. Similar results were obtained in one additional experiment. (d) Flow cytometry histograms depicts cell cycle distribution at each time point after release from double thymidine block. (e) Representative western blot shows COP1 knockdown and p53 levels in T08-387 cells. n=2 Proteins were probed on the same blot after cutting. (f) Representative western blot shows that p53 and HDAC1 co-immunoprecipitate with TRIB1 in LN18-TRIB1-FLAG cells. n=3 Proteins were probed on separate gels. (g) Gating strategy used to collect propidium iodide positive cells.

SUPPLEMENTARY FIGURE 7

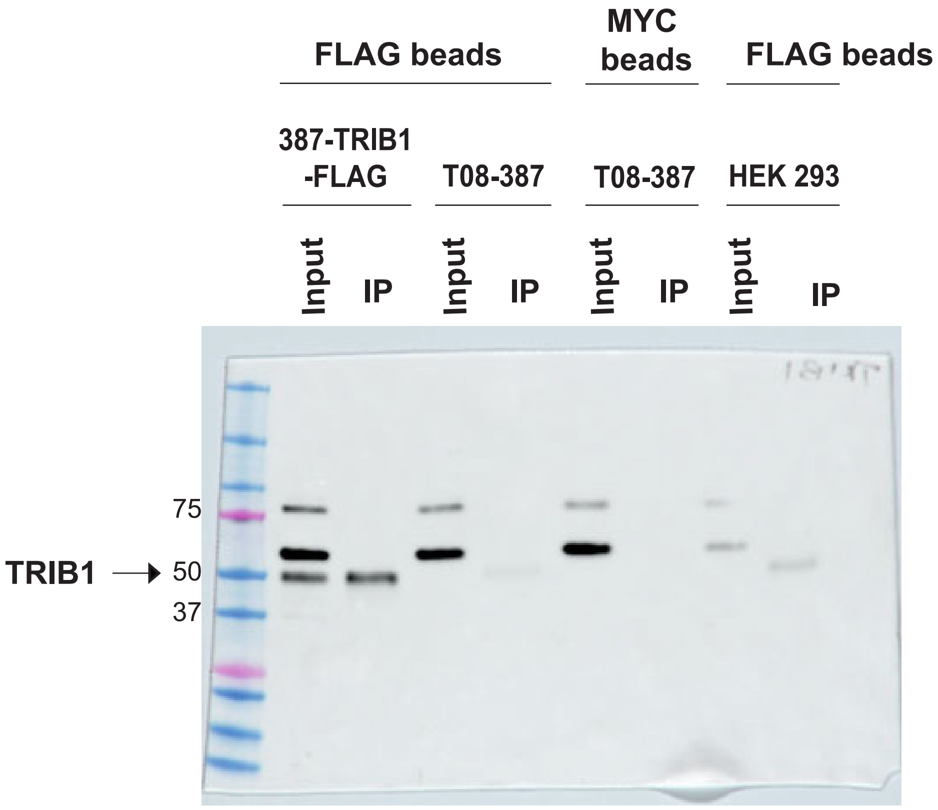

Figure 2c LN18 TRIB1 RT

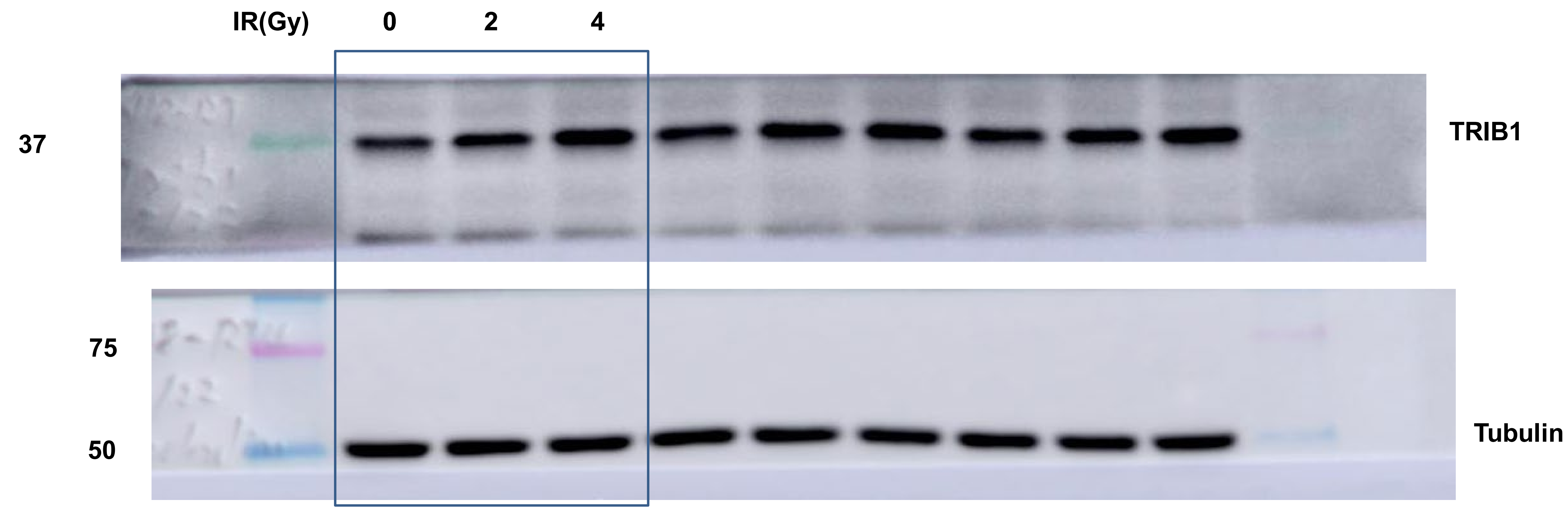

Replicate 1

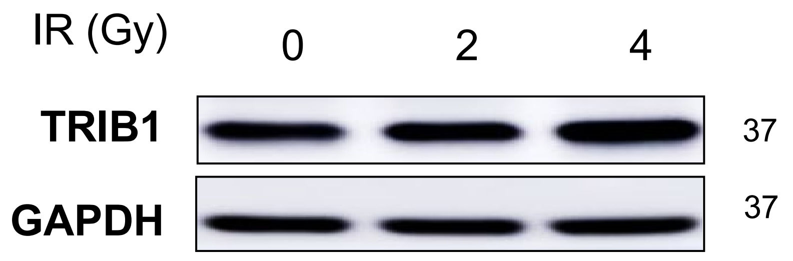

Figure 2d LN18 TRIB1 TMZ

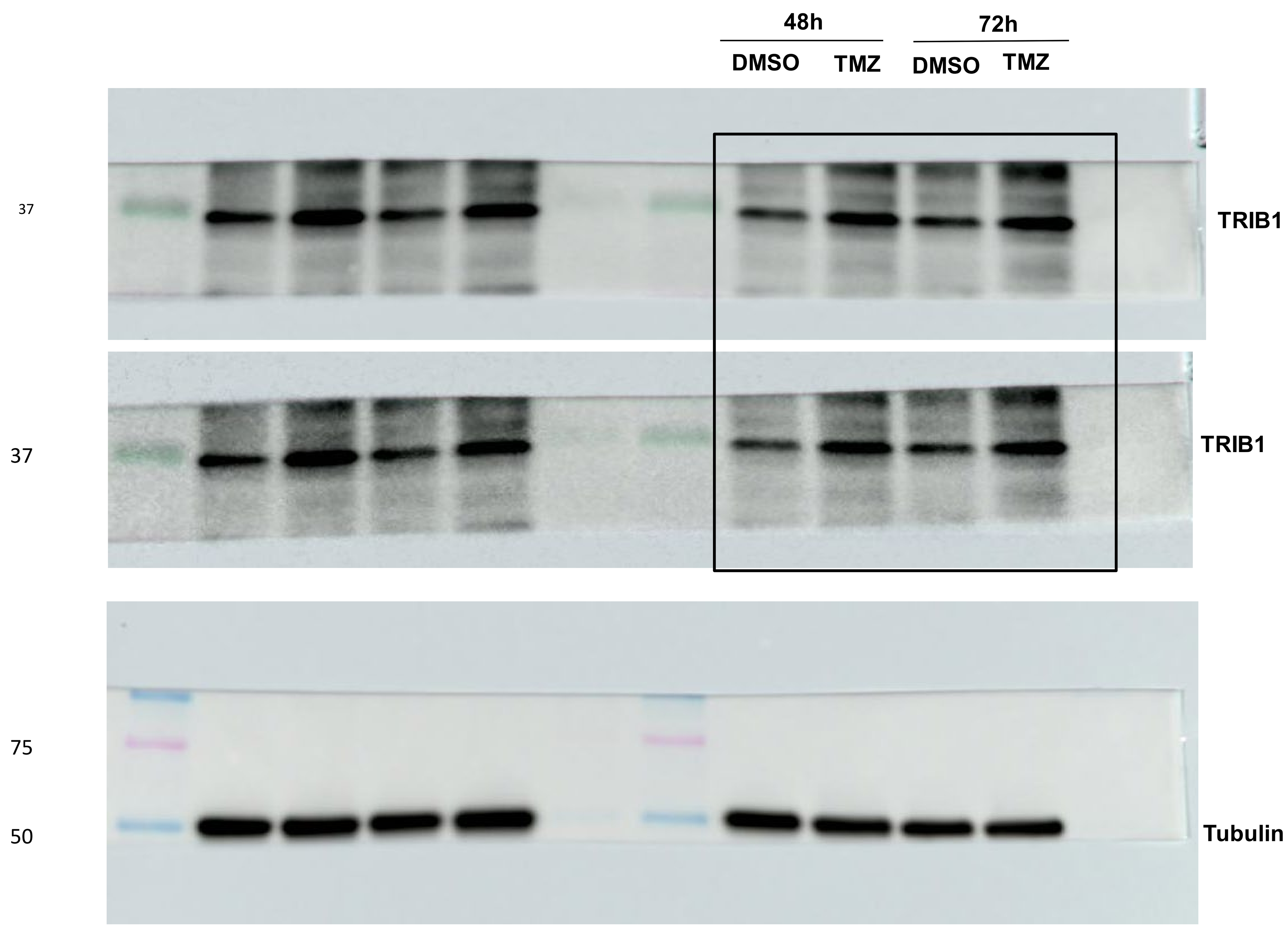

Figure 3a GBM30-luc c-PARP, c-CASPASE3, TRIB1 IR

Representative

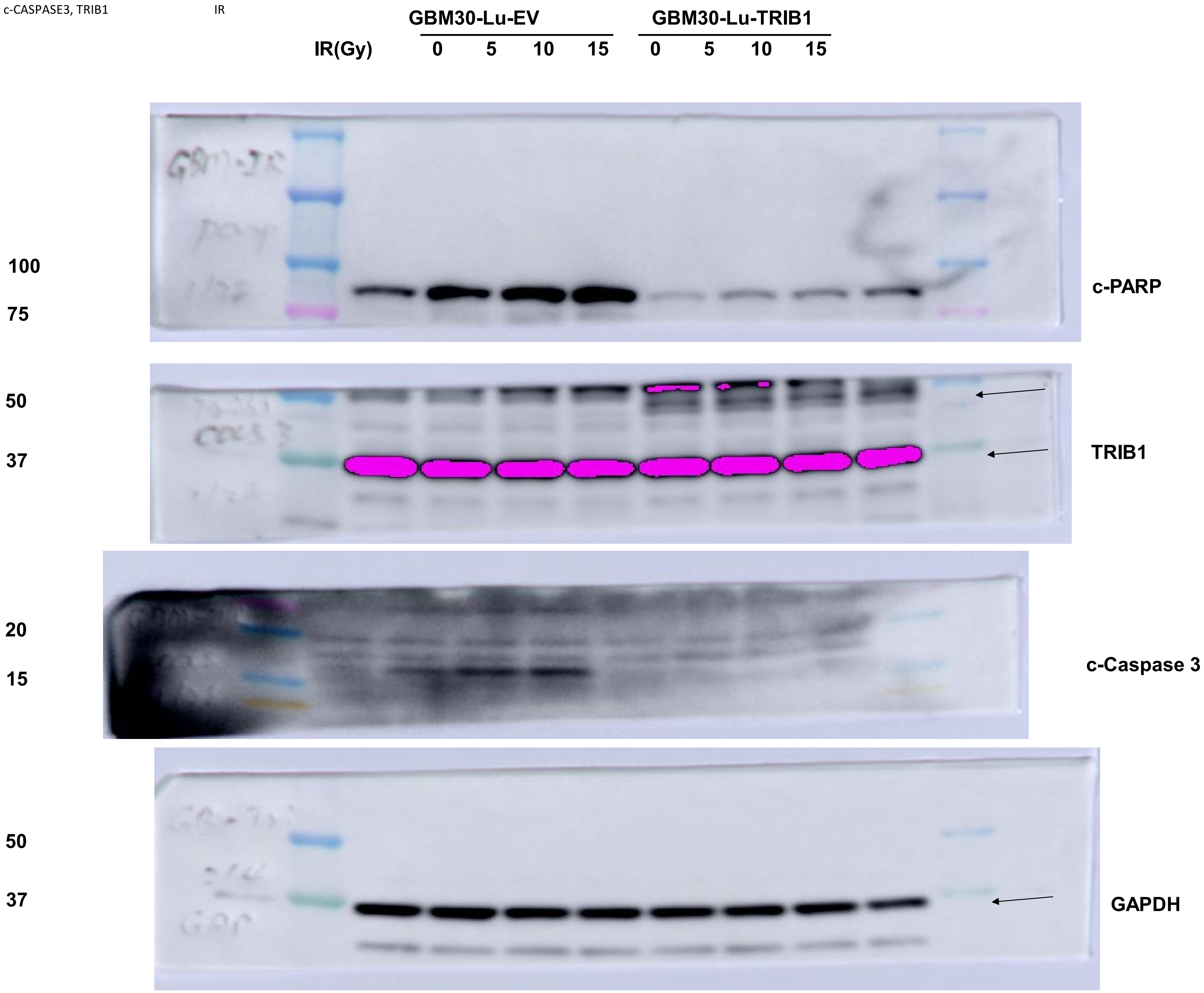

Replicate 1

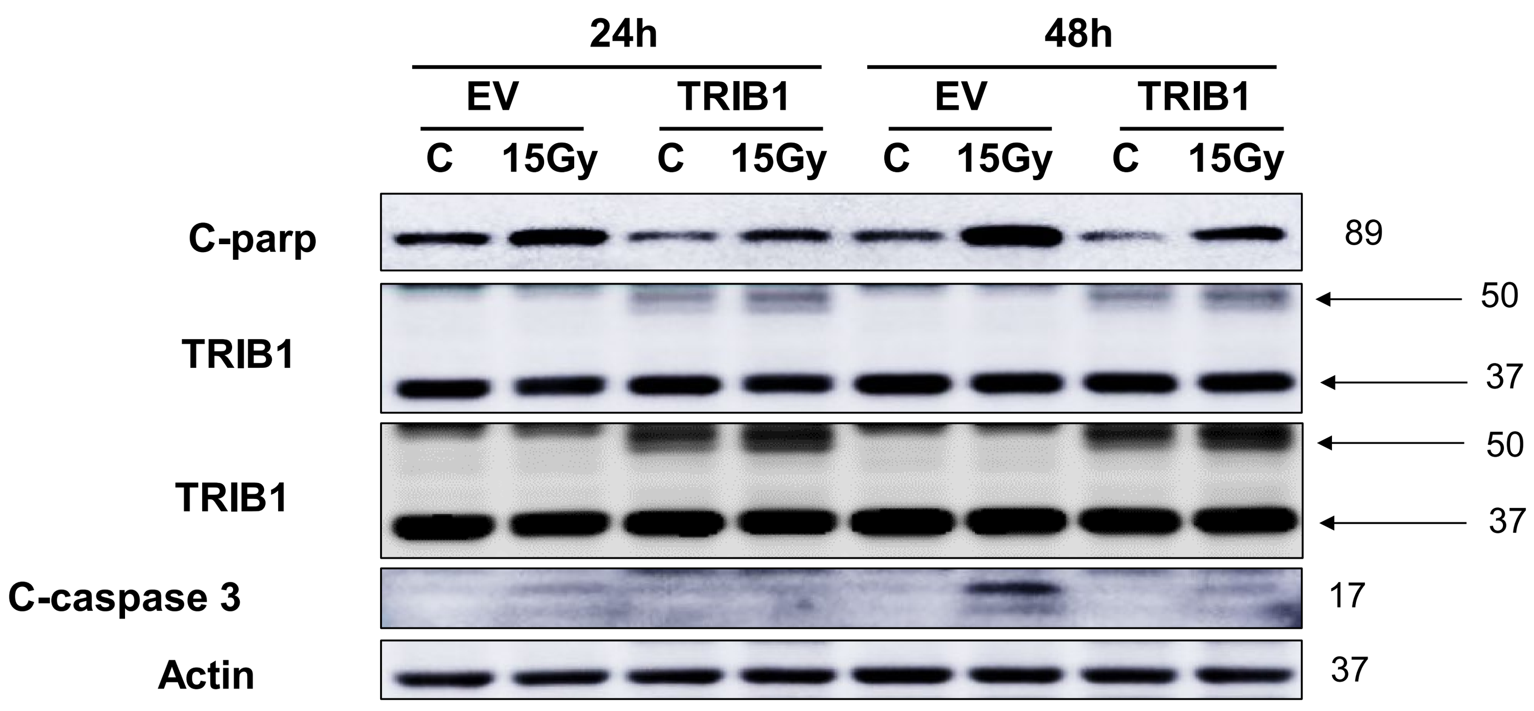

Figure 3b T08-387 c-PARP, c-CASPASE3, TRIB1 IR

Representative

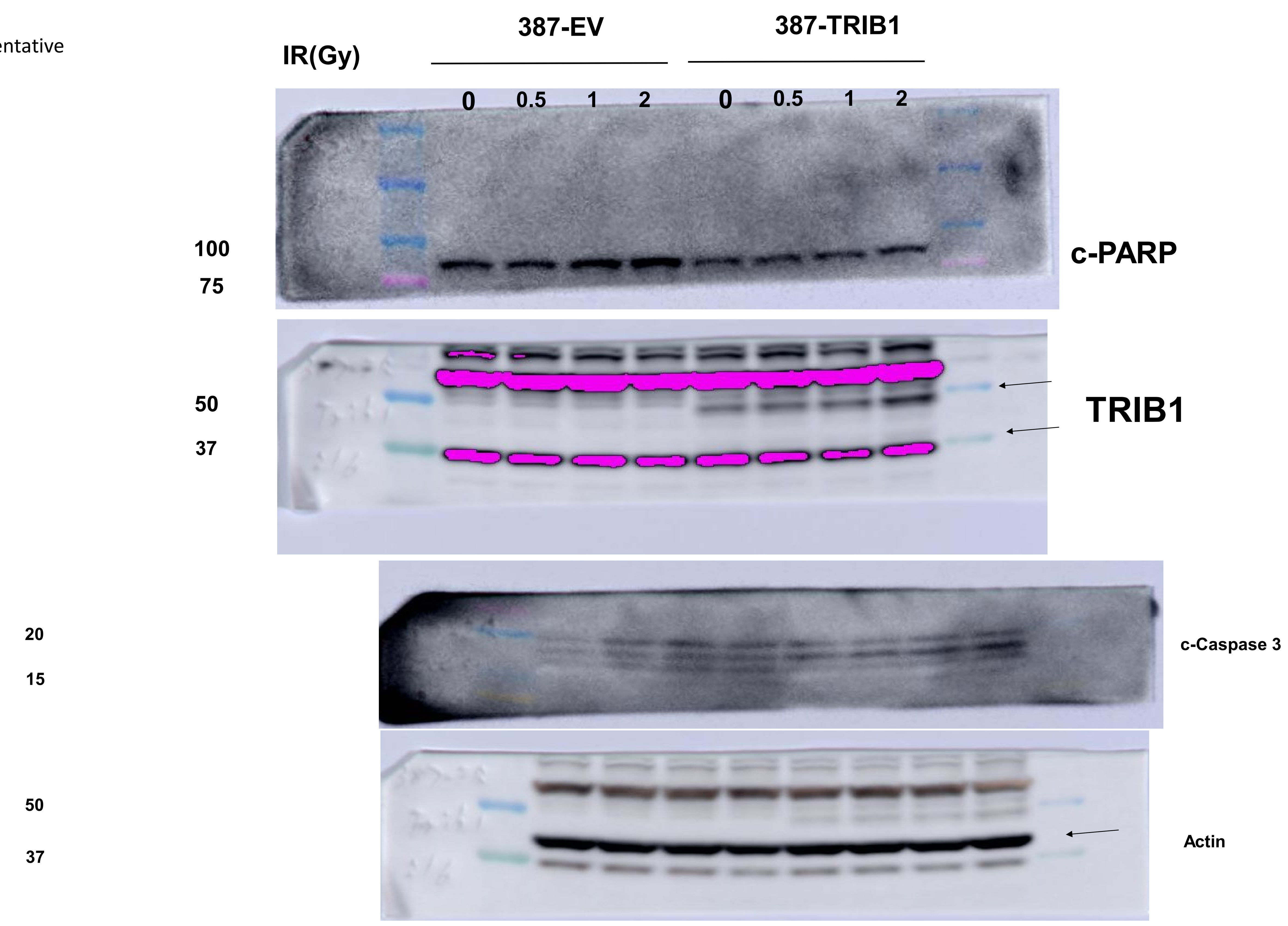

Replicate 1

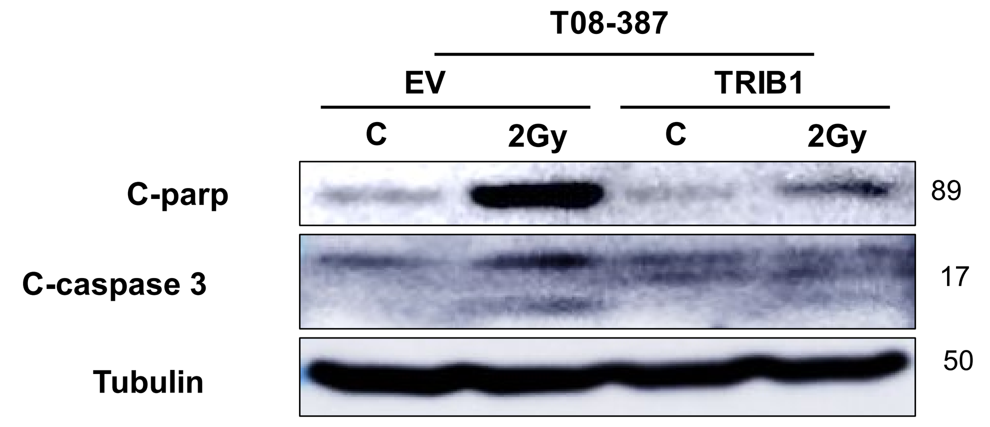

Figure 3c GBM30-luc c-PARP, c-CASPASE3, TRIB1 IR

Representative

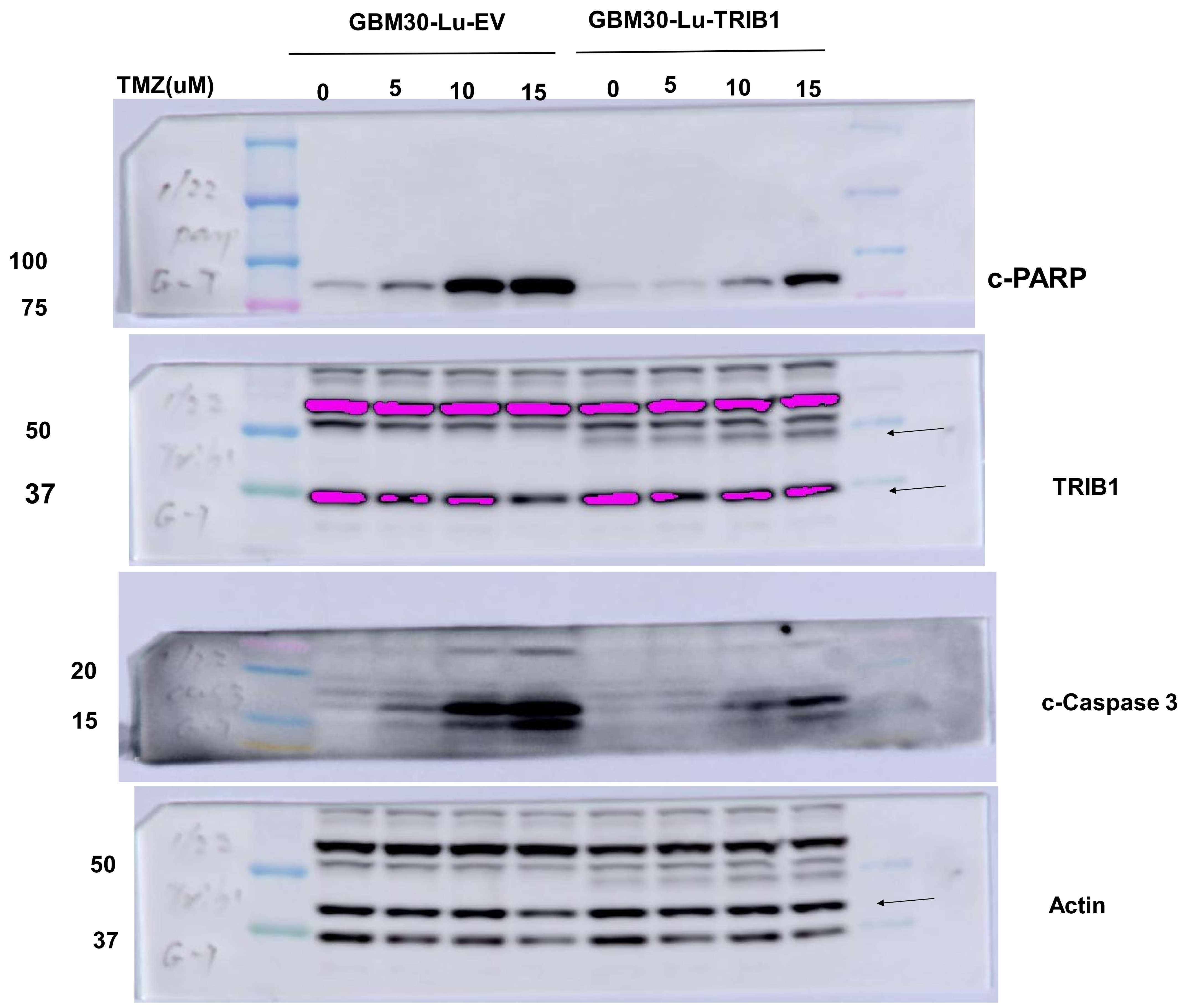

Replicate 1

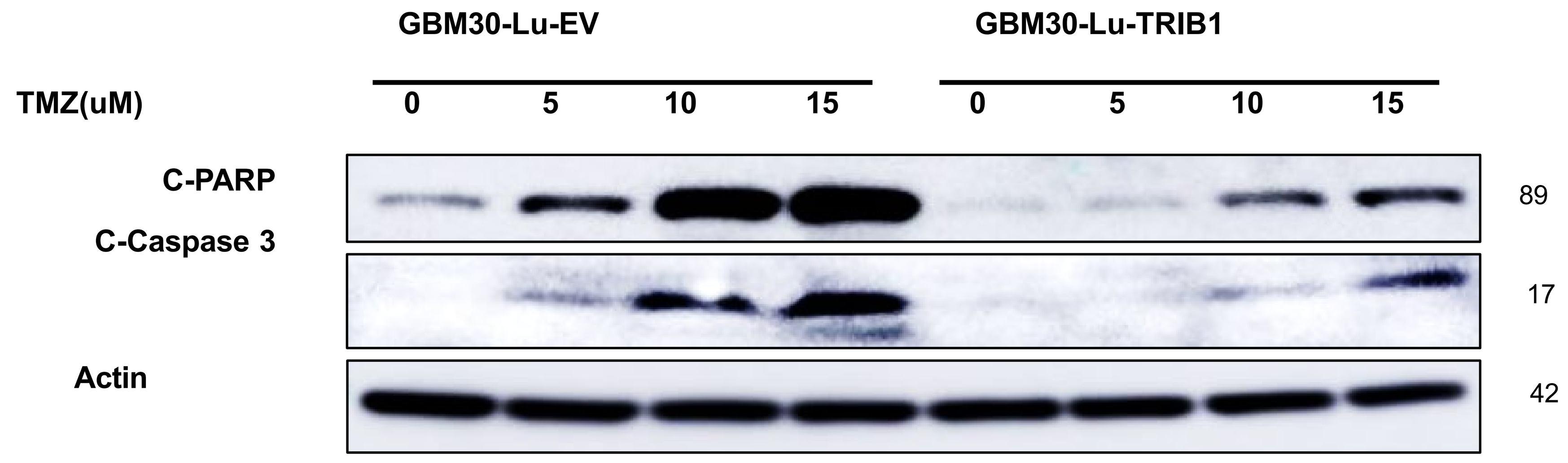

Figrue 3d 387-TRIB-FLAG RT TRIB1

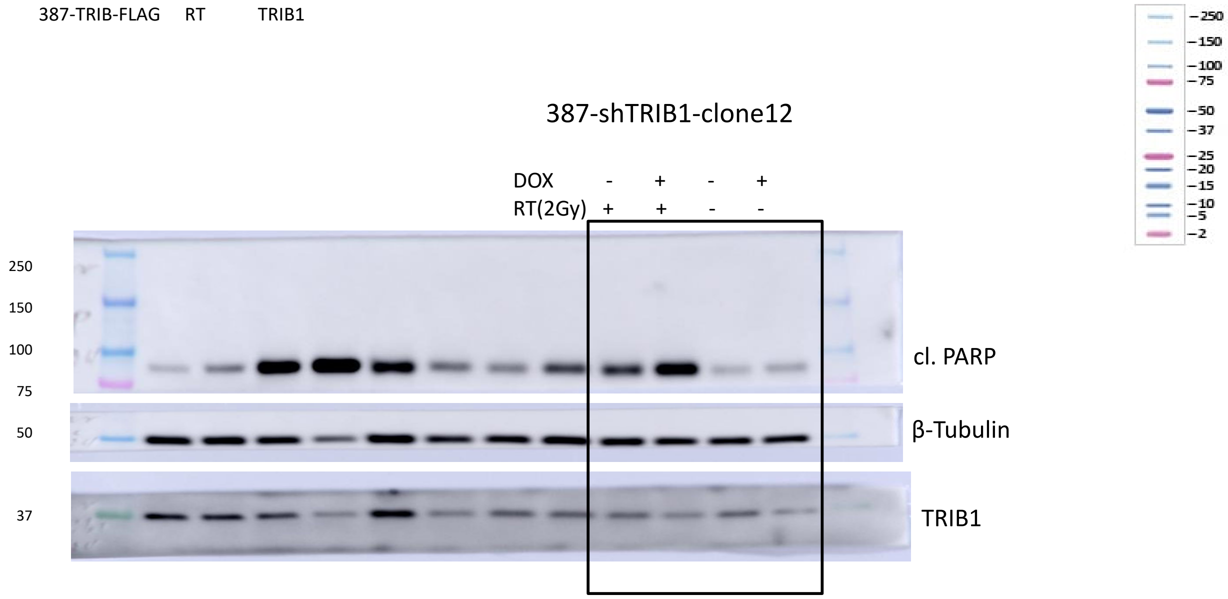

Replicate 1

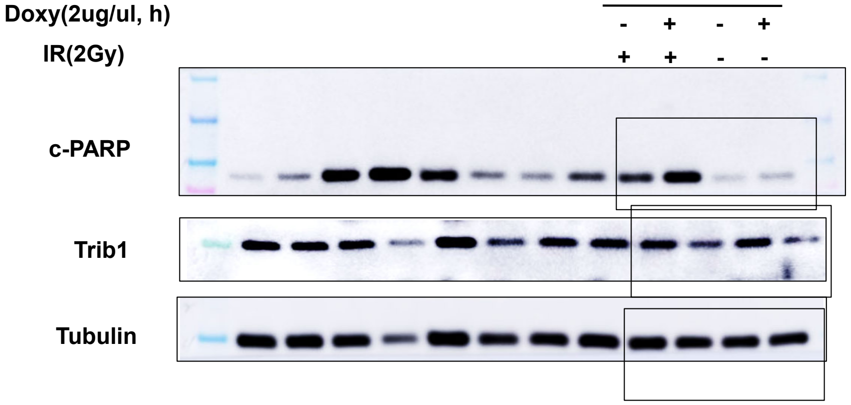

Figure 4a T08-387 GBM30-luc P-ERK and P-AKT

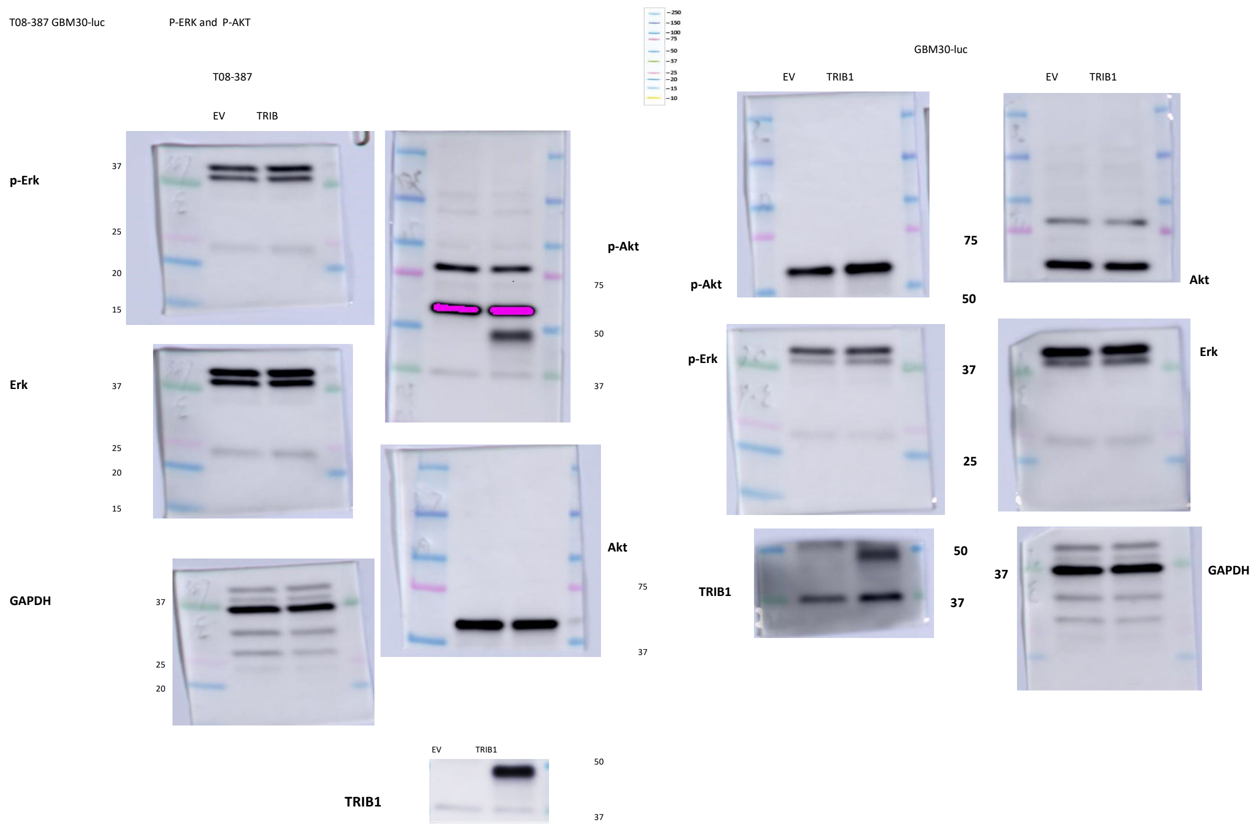

Replicate 1

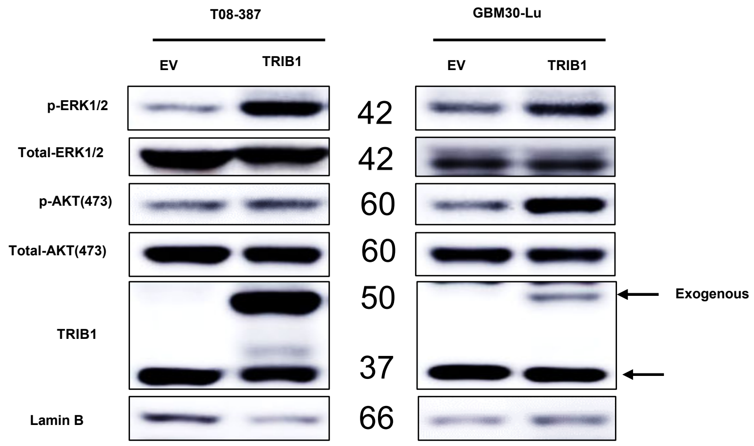

Figure 4b T08-387 TRIB1 knockdown P-ERK and P-AKT

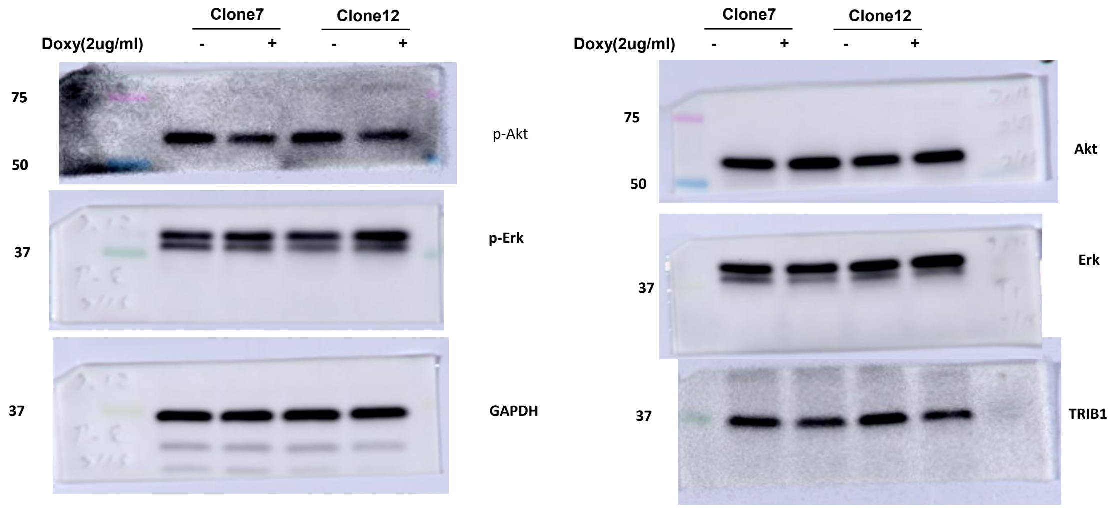

Replicate 1

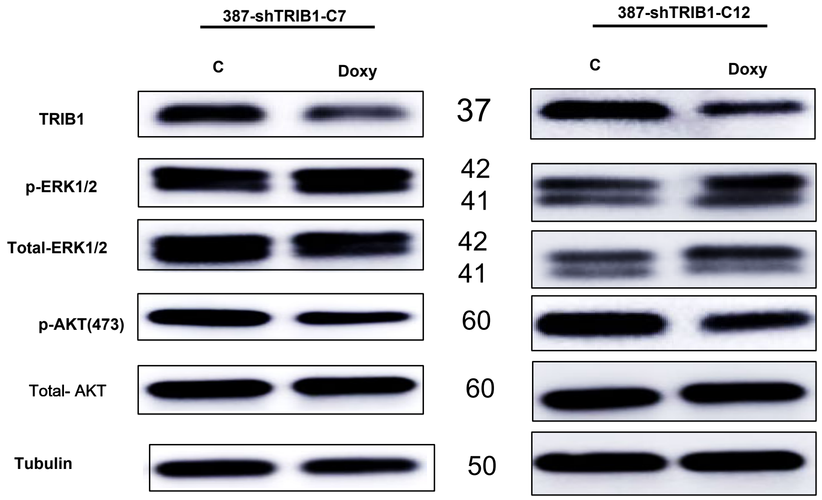

Figure 5a 387-TRIB1-FLAG MEK1-HA IP:FLAG

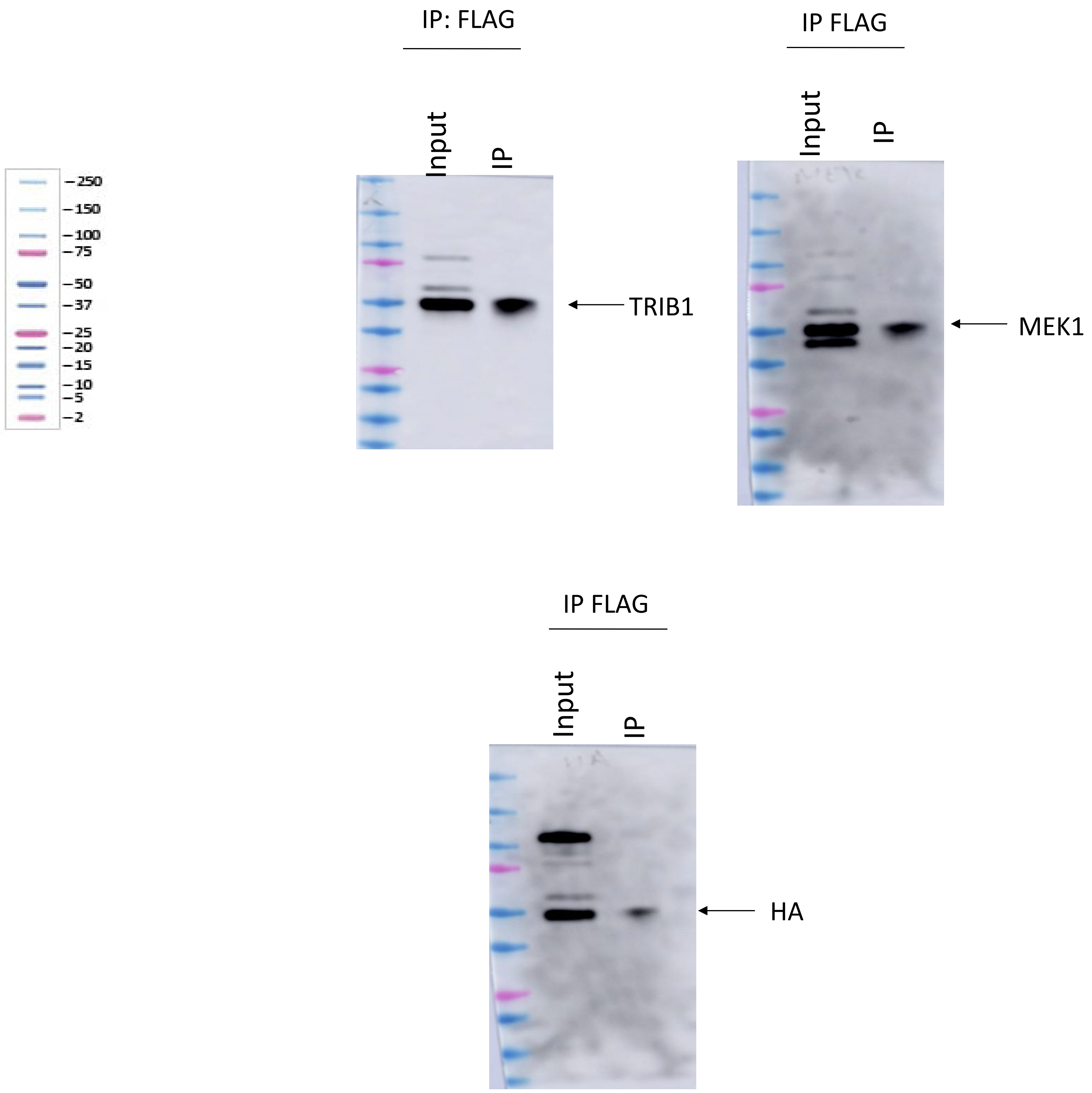

Figure 5b 387-TRIB1-MYC IP:MYC

IP MYC

Input IP

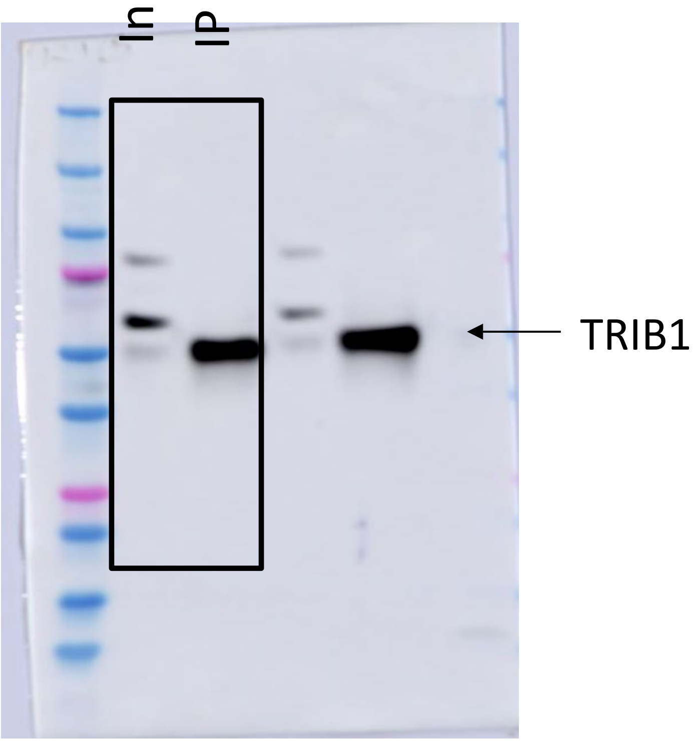

TRIB1

IP MYC

Input IP

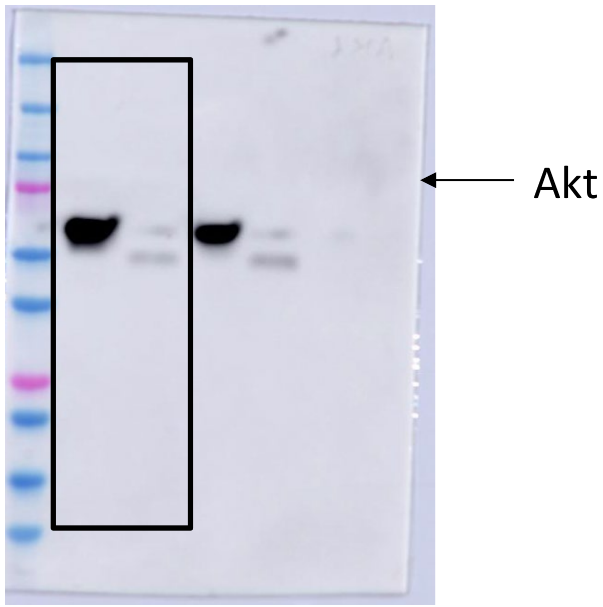

Akt

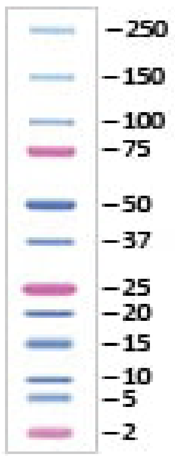

Figure 5c 387-TRIB1-FLAG and 387-TRIB1-W337A-FLAG

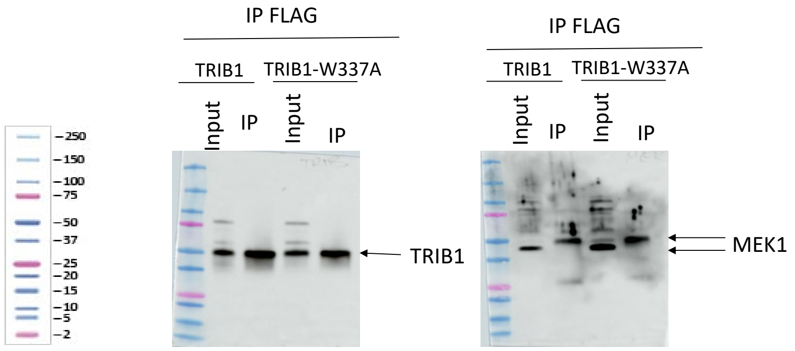

Replicate 1

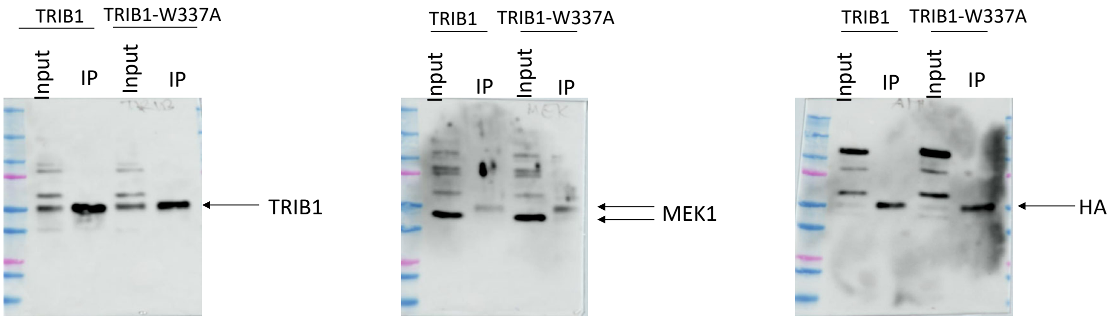

Figure 5d 387-TRIB-MYC and 387-TRIB1-W337A- MYC

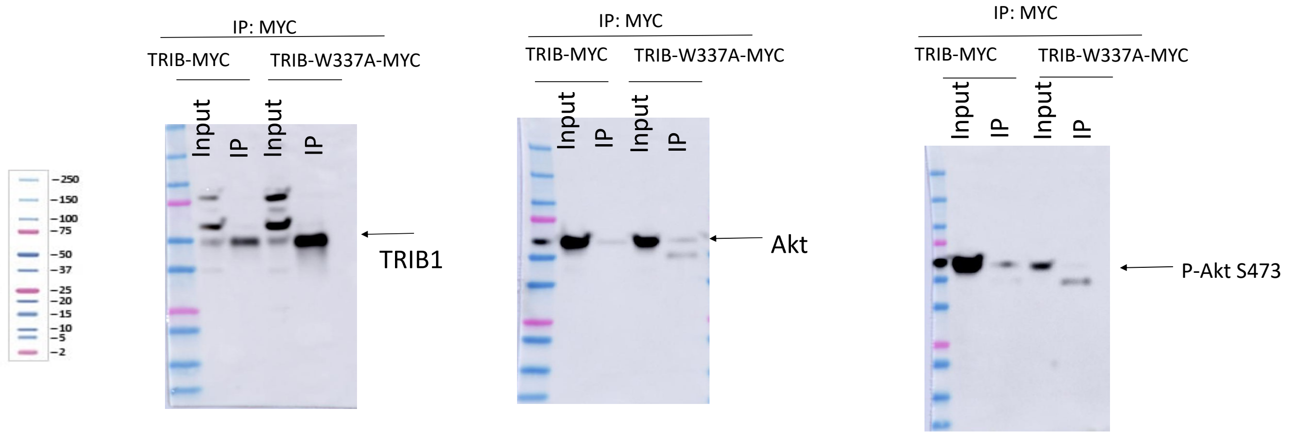

Replicate 1

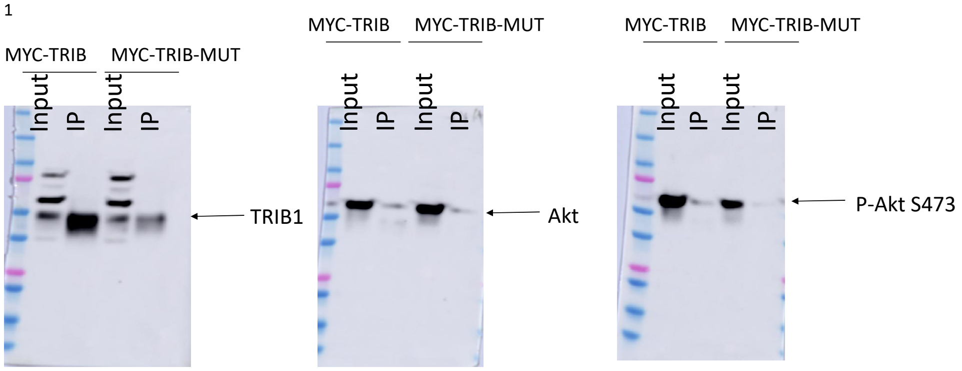

Figure 5e 387-TRIB-FLAG wortmanin

Replicate 1

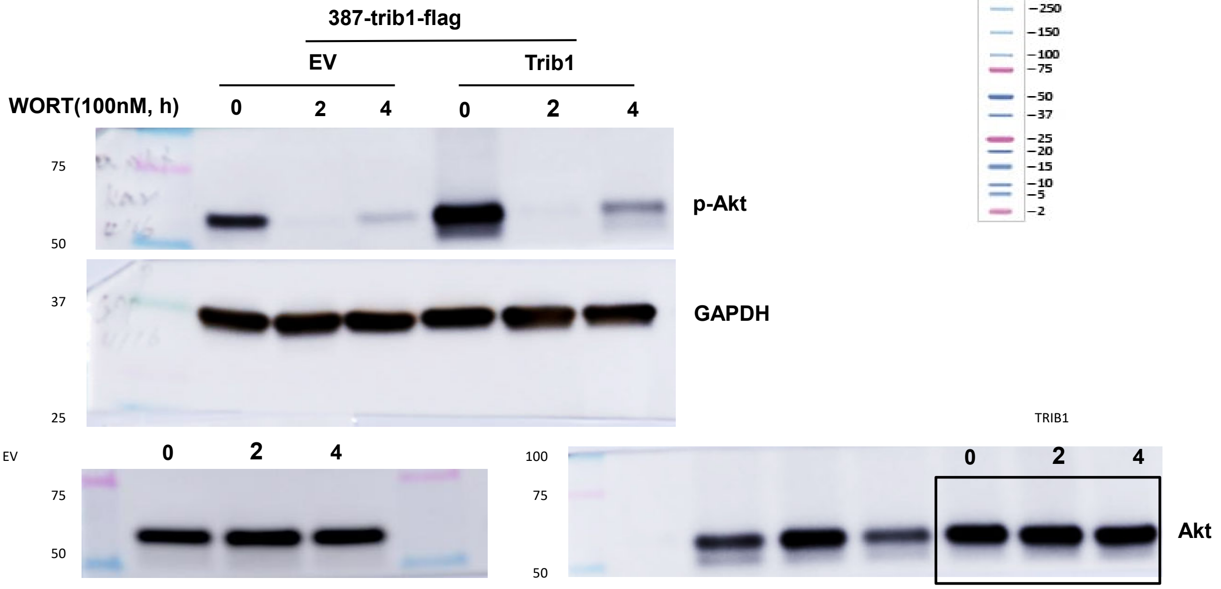

Representative

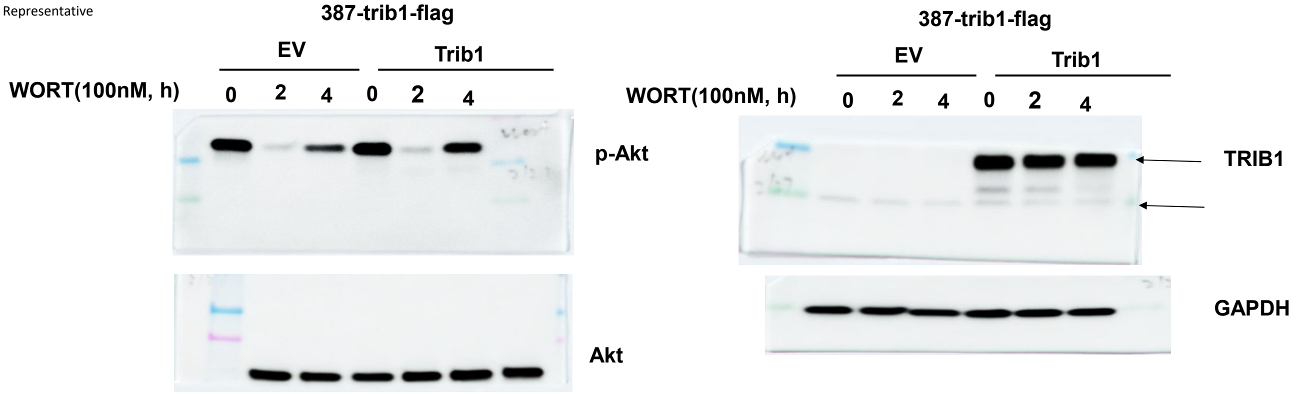

Figure 5g HEK 293 TRIB1 deletion mutants Representative

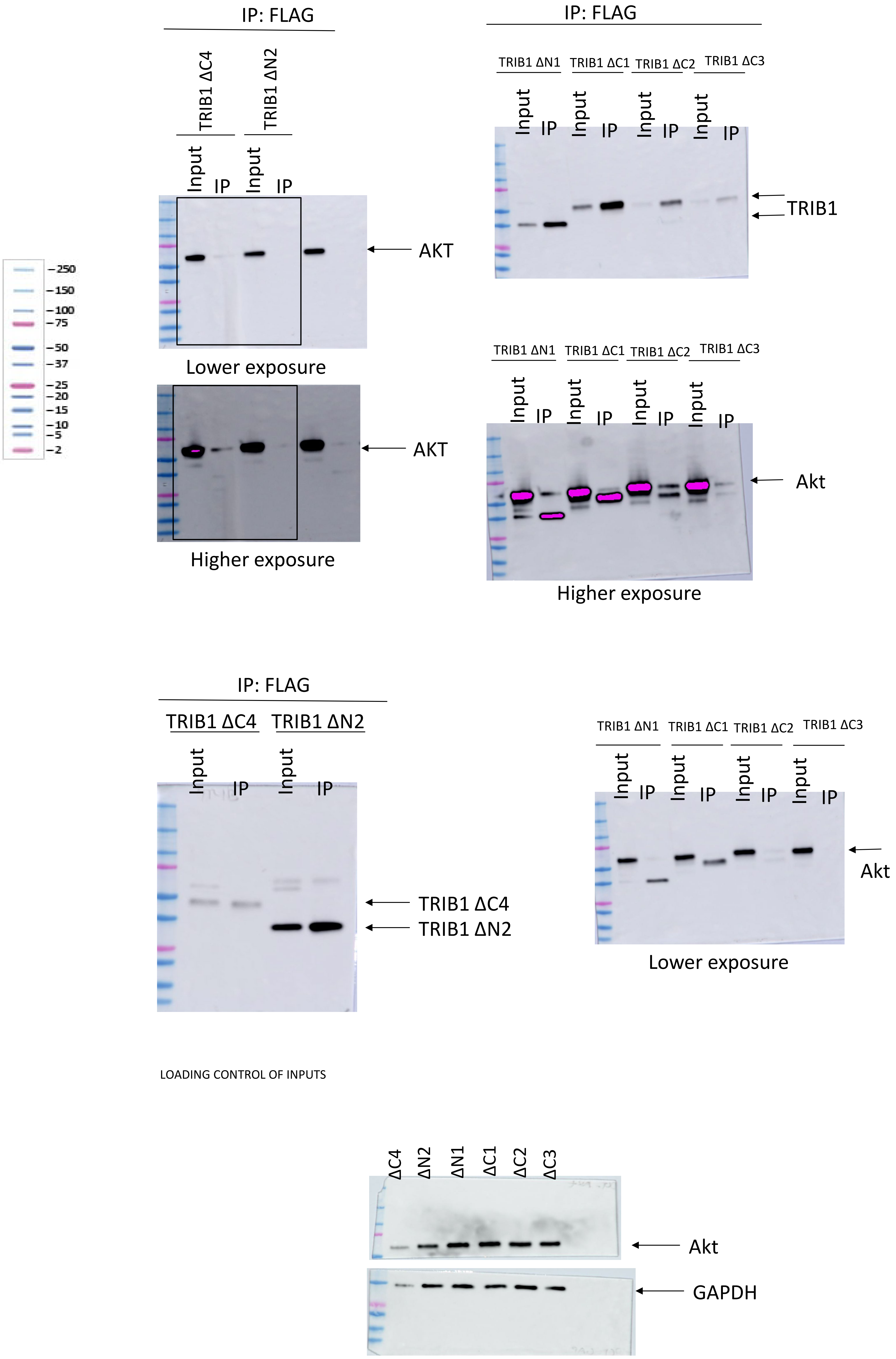

Figure 5g HEK 293 TRIB1 deletion mutants

Replicate 1

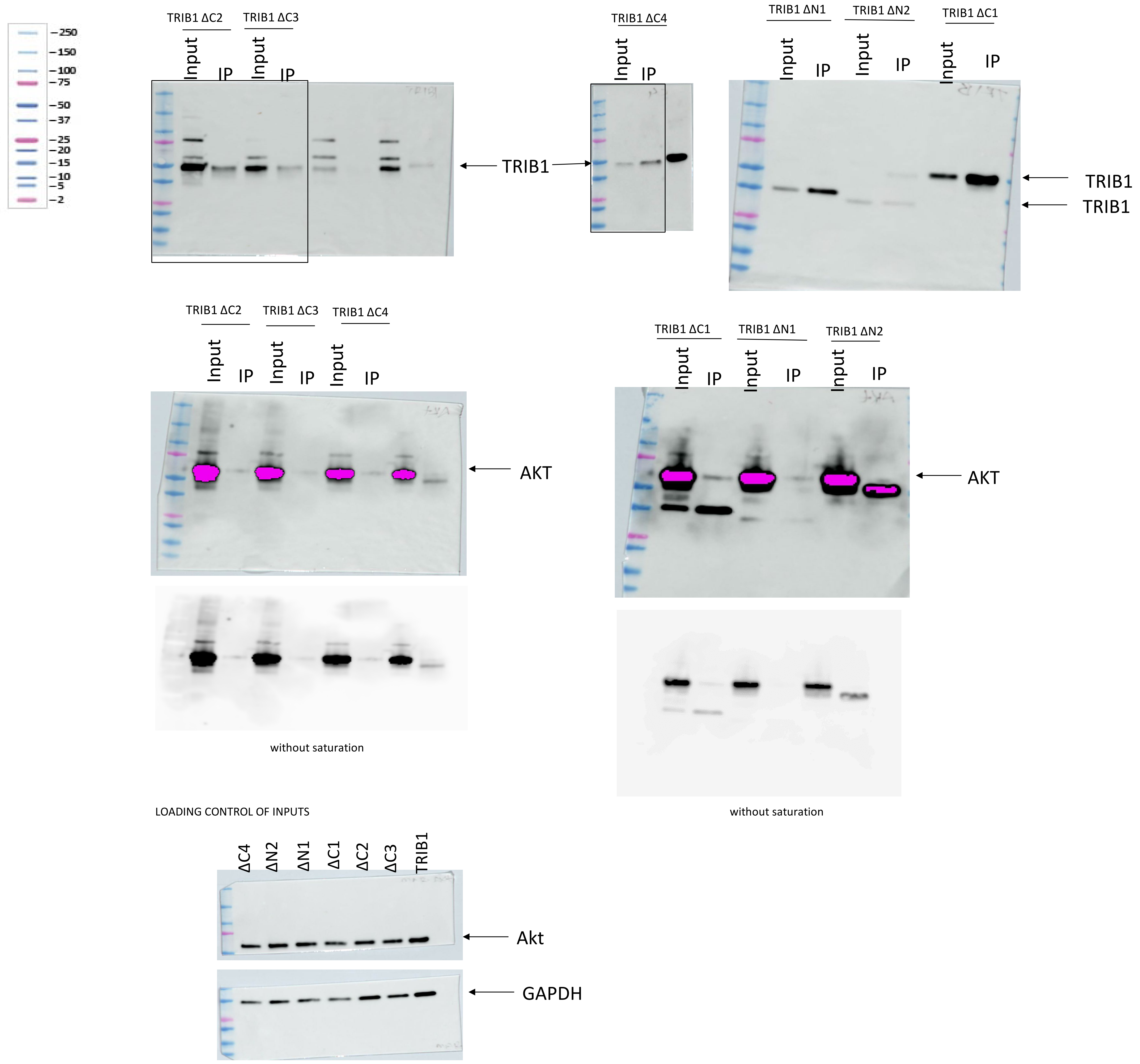

Figrue 6b U87 MG double thymidine block

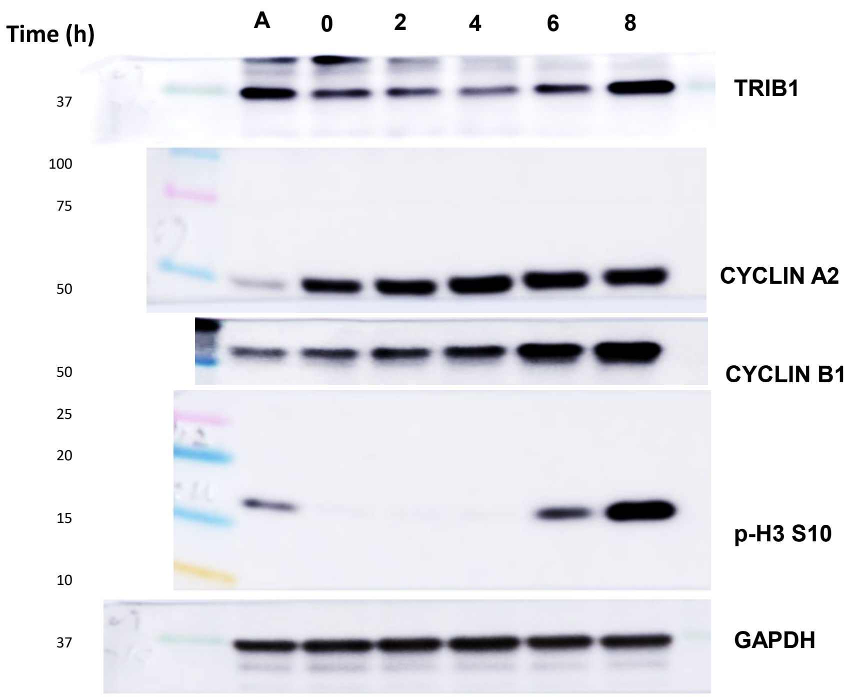

Replicate 1

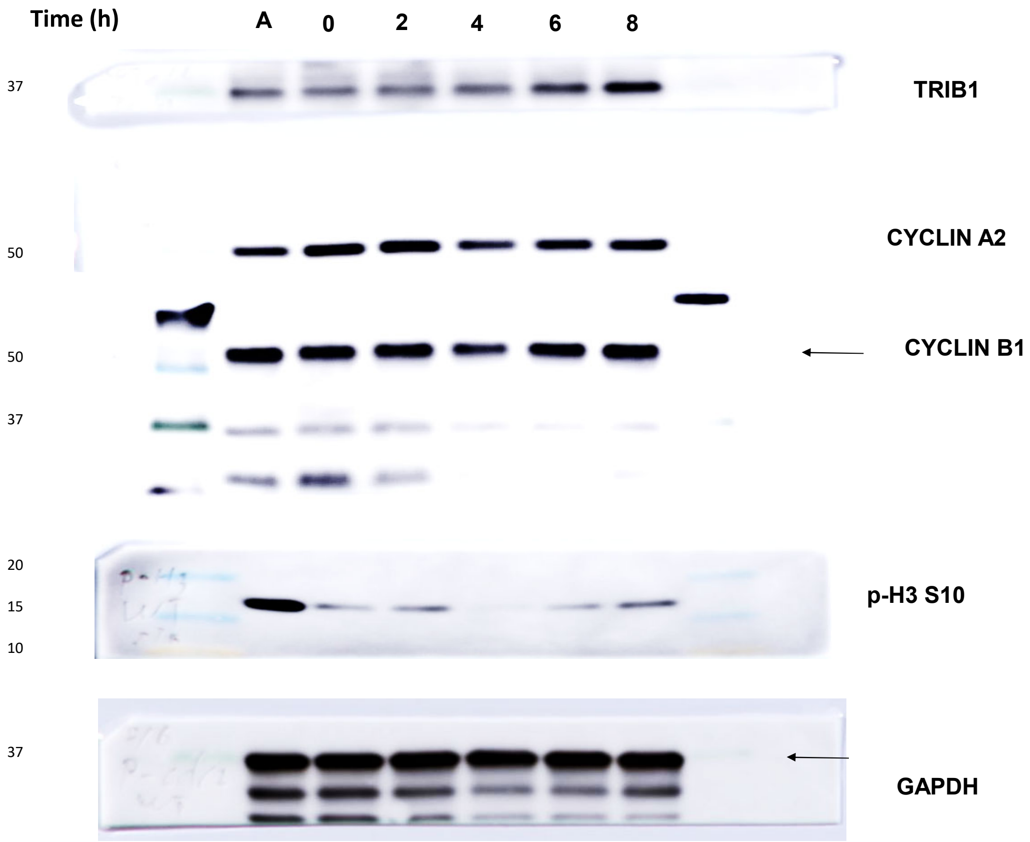

Figure 6d U87 MG RO-336

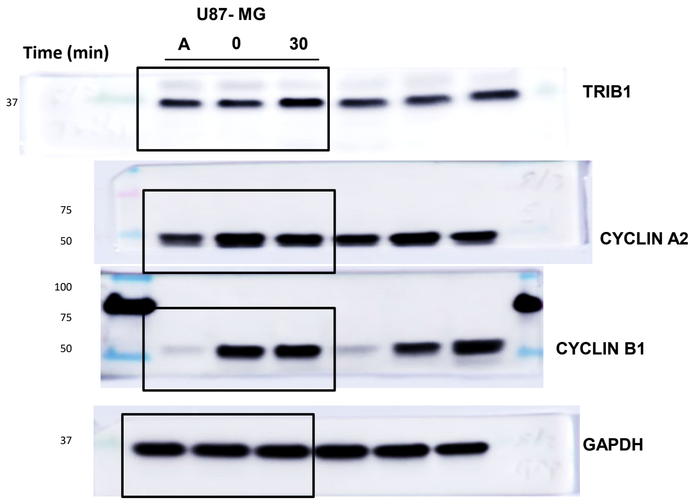

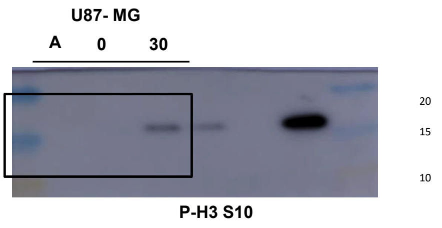

Replicate 1

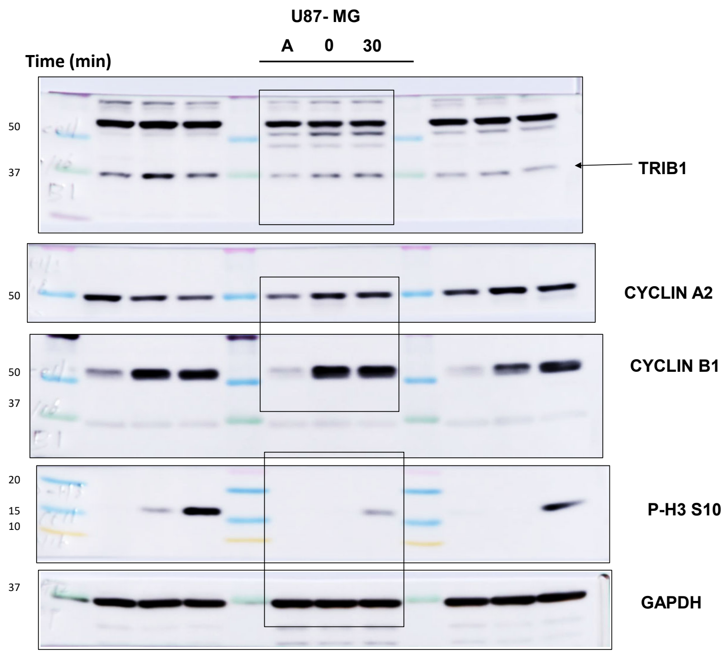

Figure 6e 387-TRIB1-FLAG IP:FLAG

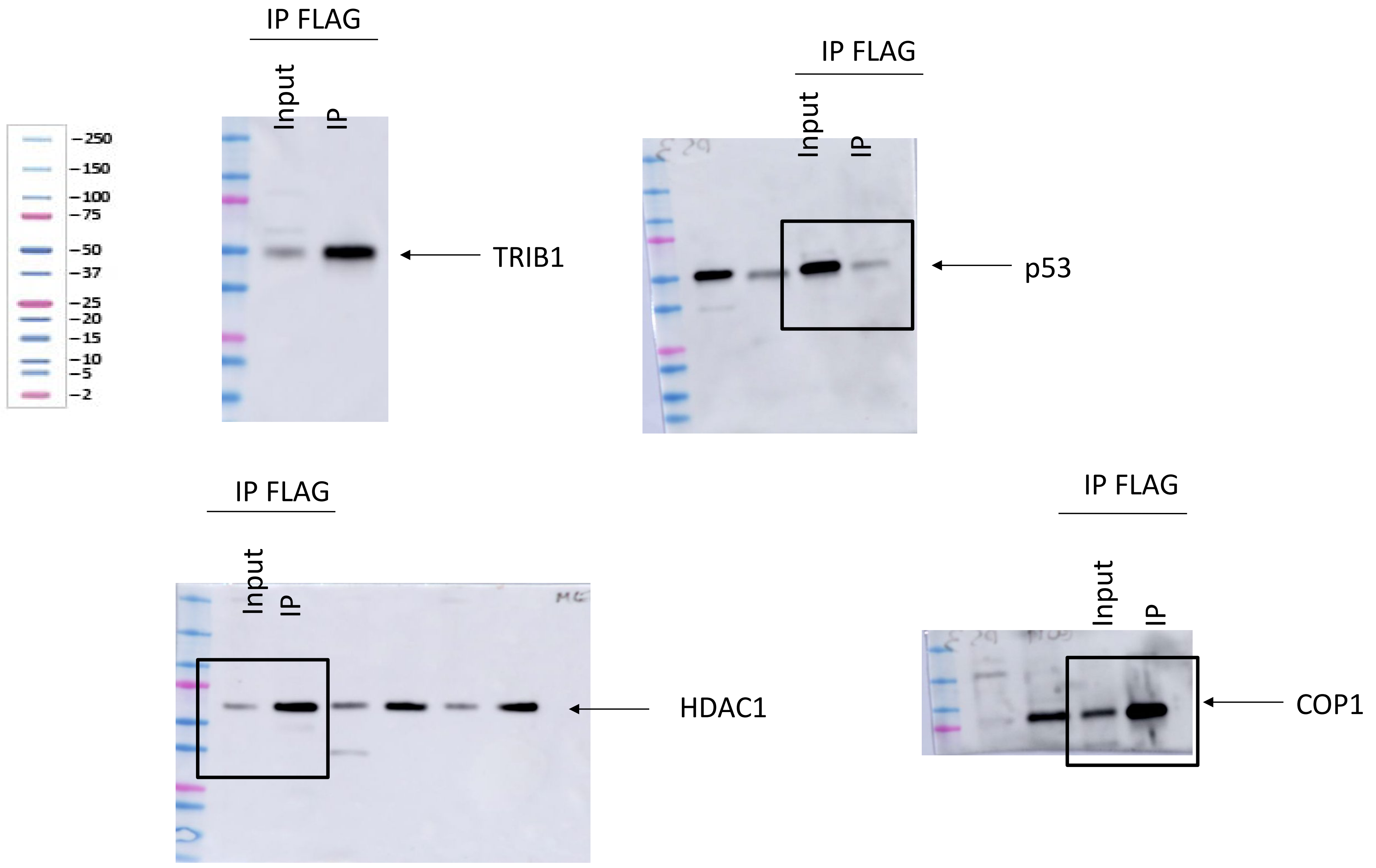

Figure 6f GBM30-TRIB1-FLAG IP:FLAG

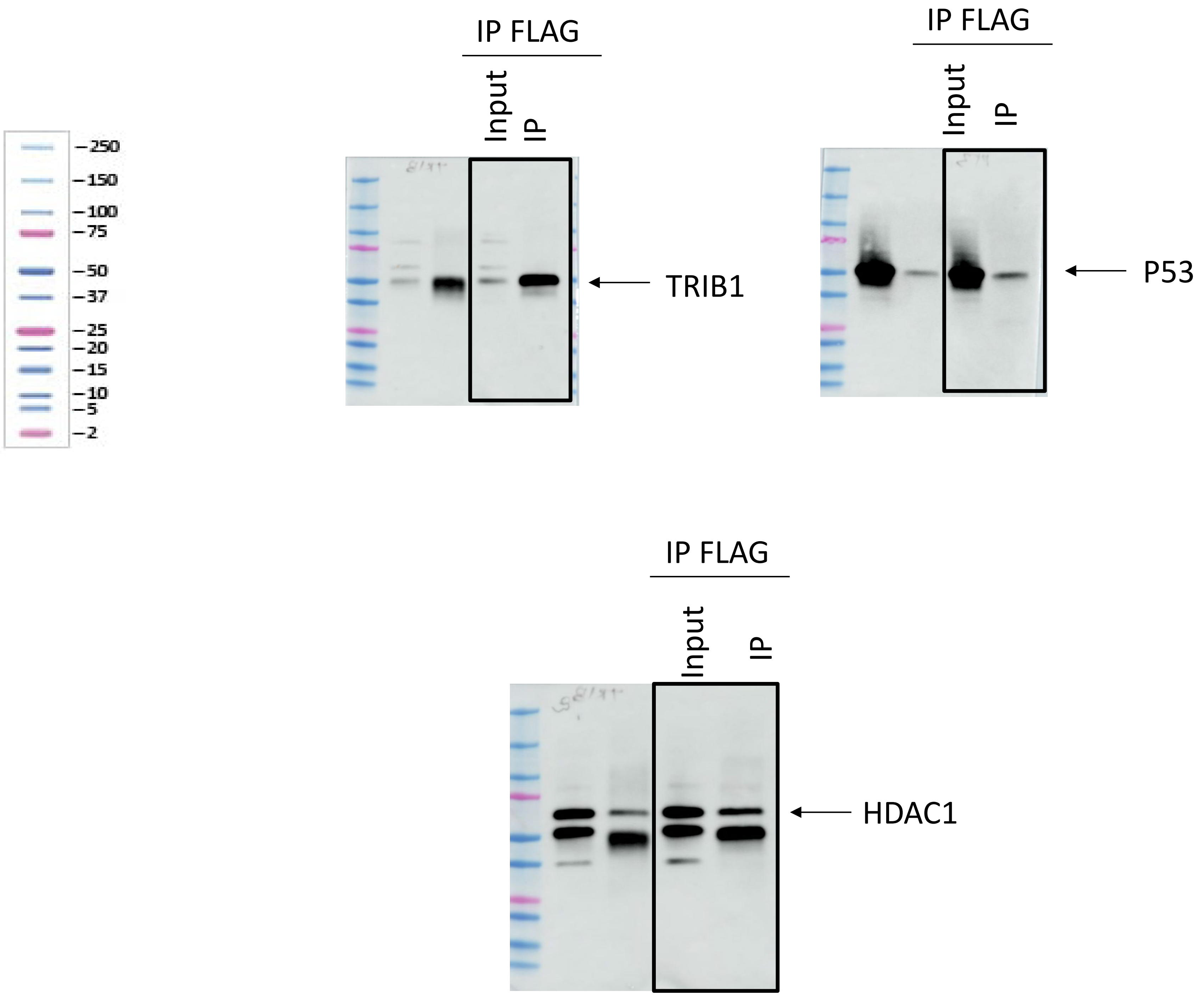

Figure 6g 3359-parental and 3359-p53 R248W

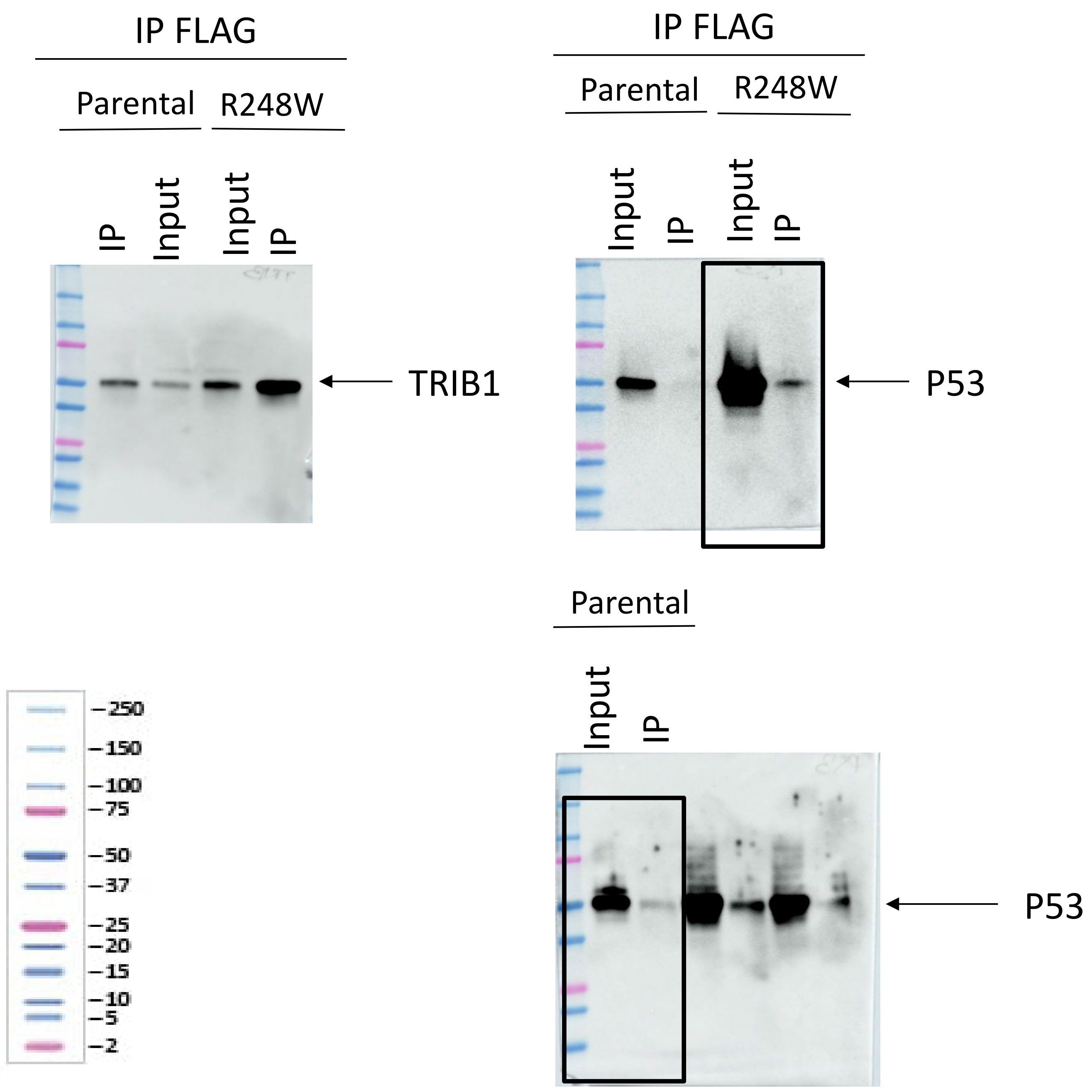

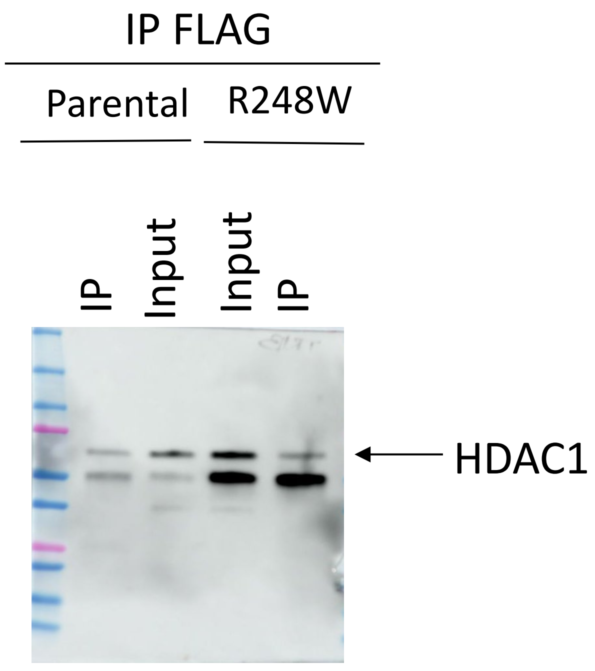

Re-blot from TRIB1

Figure 6h 387-TRIB1-FLAG shCOP1

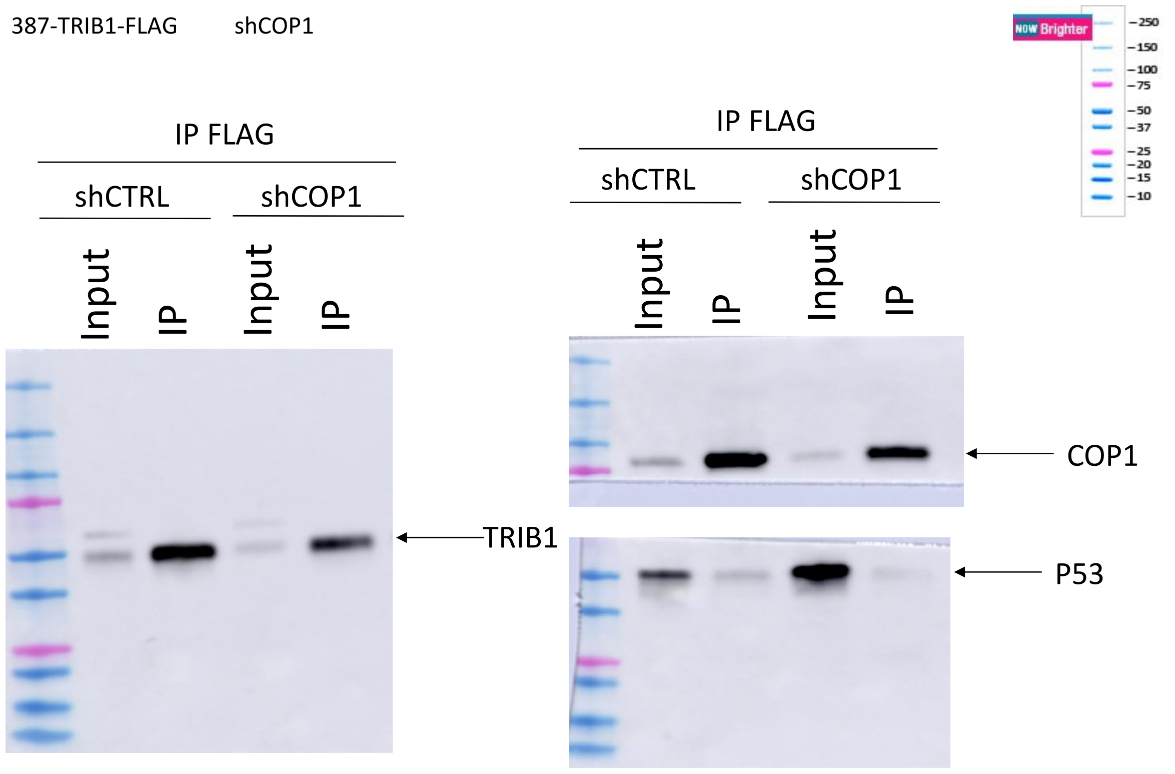

Replicate 1

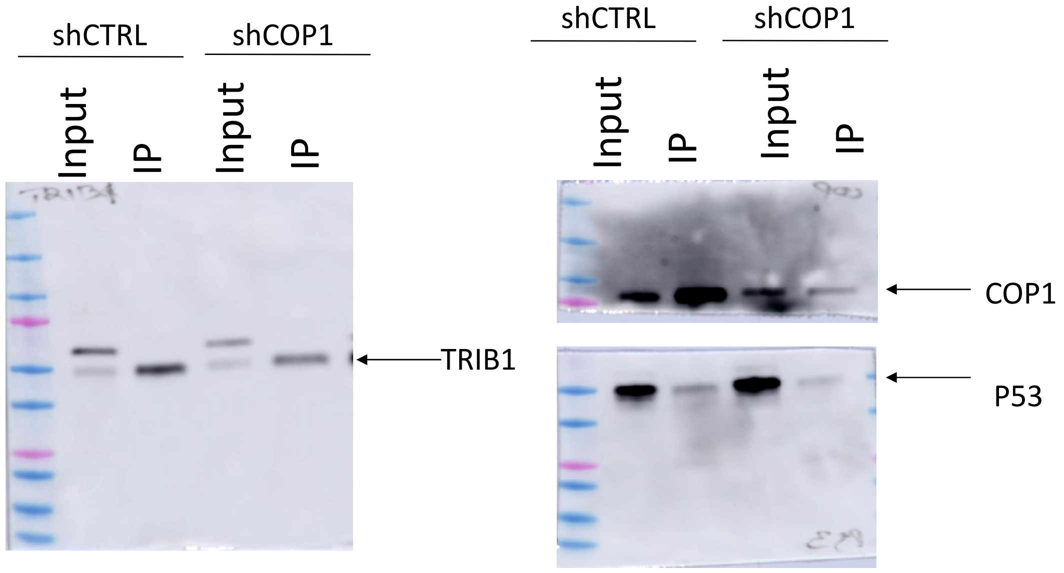

Supplementary fig. 3b

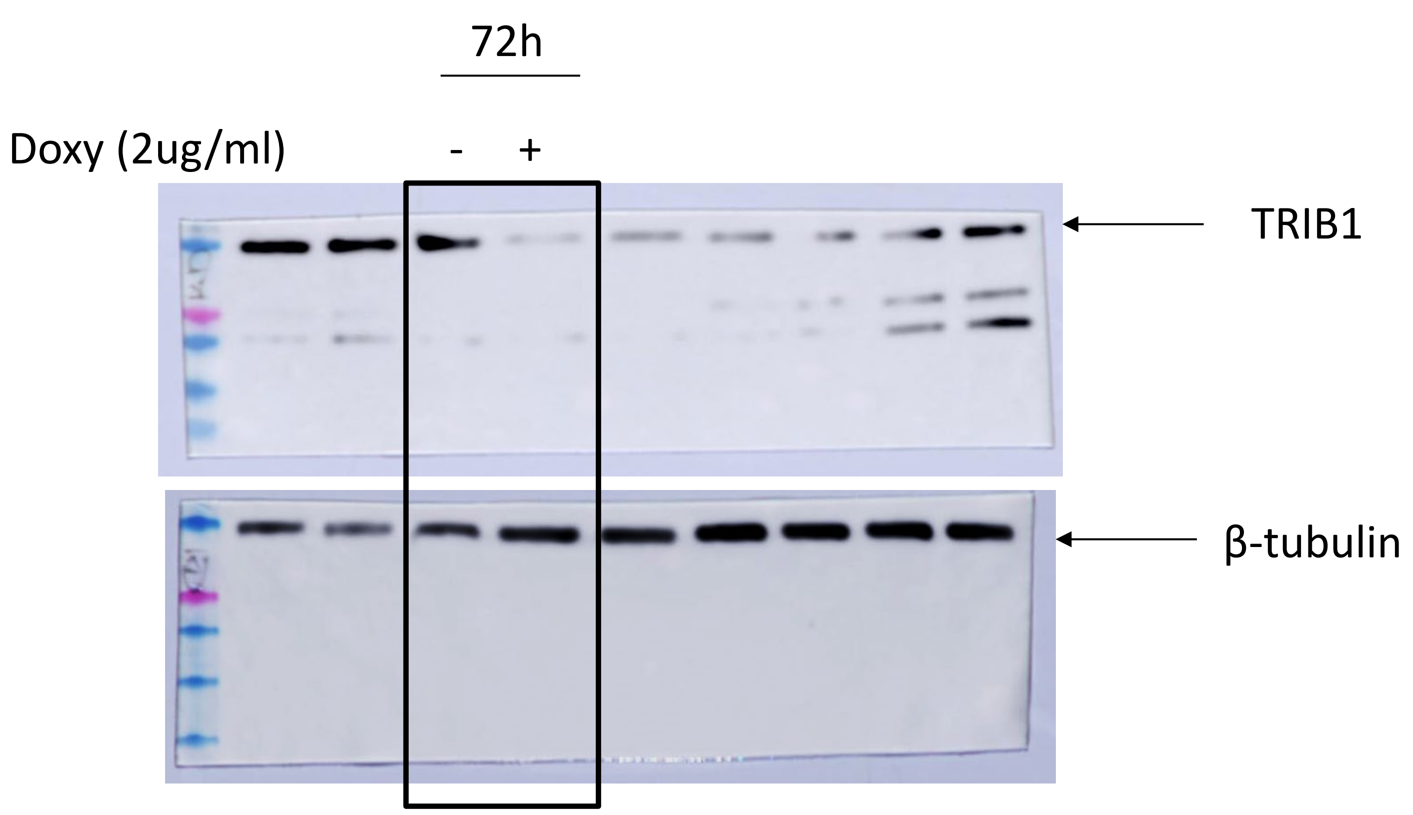

Supplementary fig. 4 LN18 IR c-PARP, c-CASPASE3

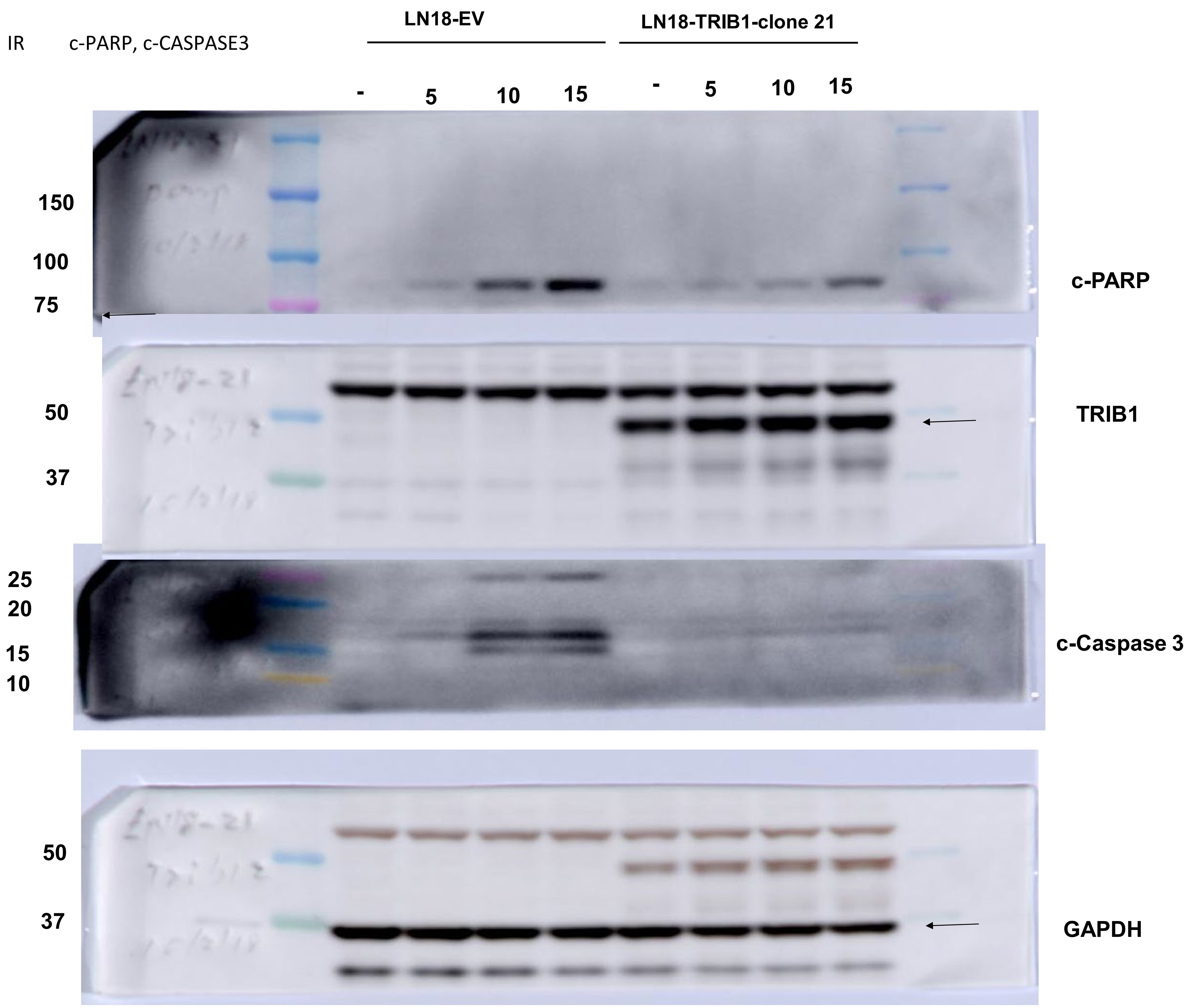

Supplementary fig. 5a TRIB1, P-AKT S473

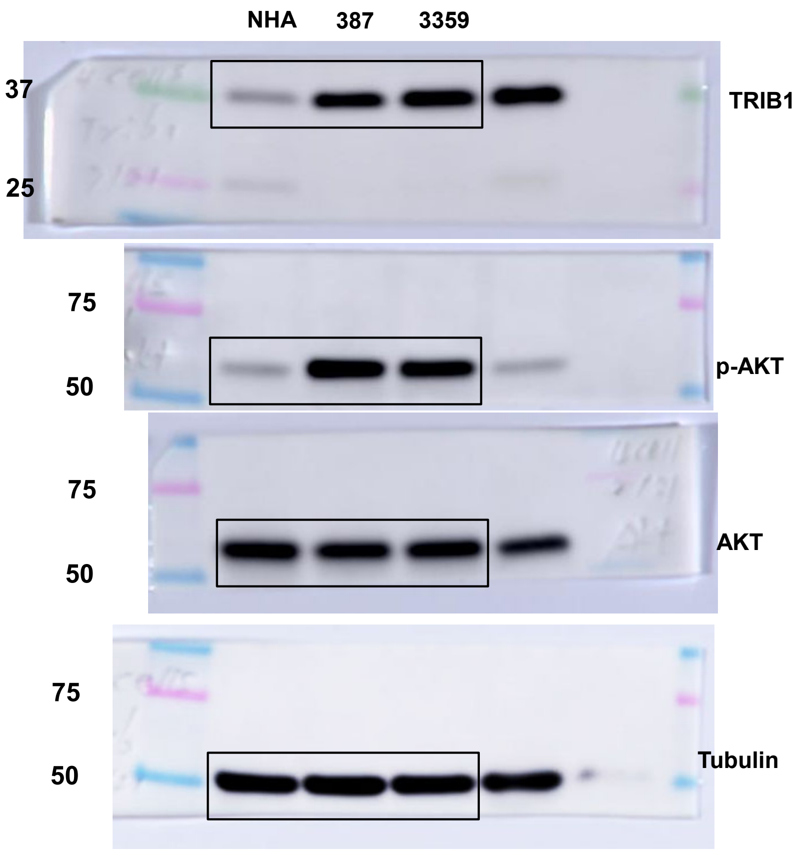

Replicate 1

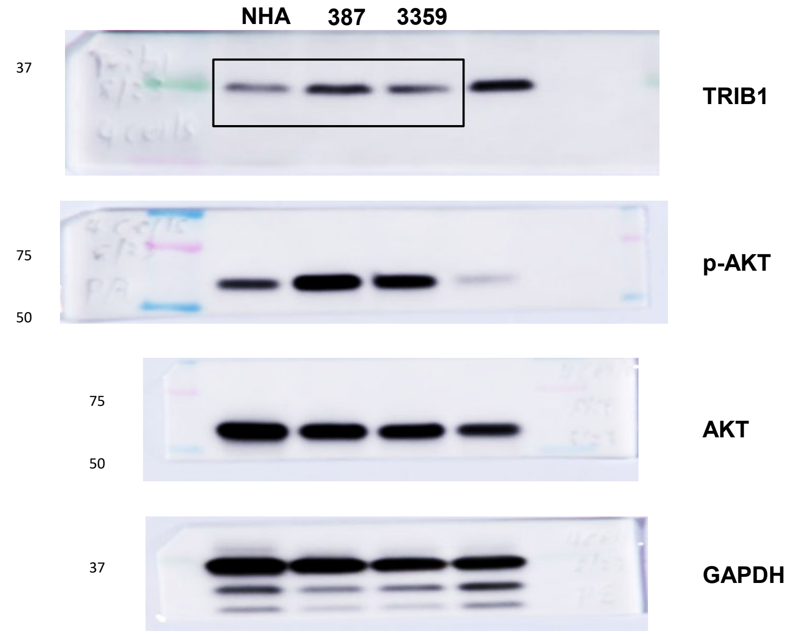

Supplementary fig. 5b T08-387-TRIB1-MYC p-erk p-akt S473

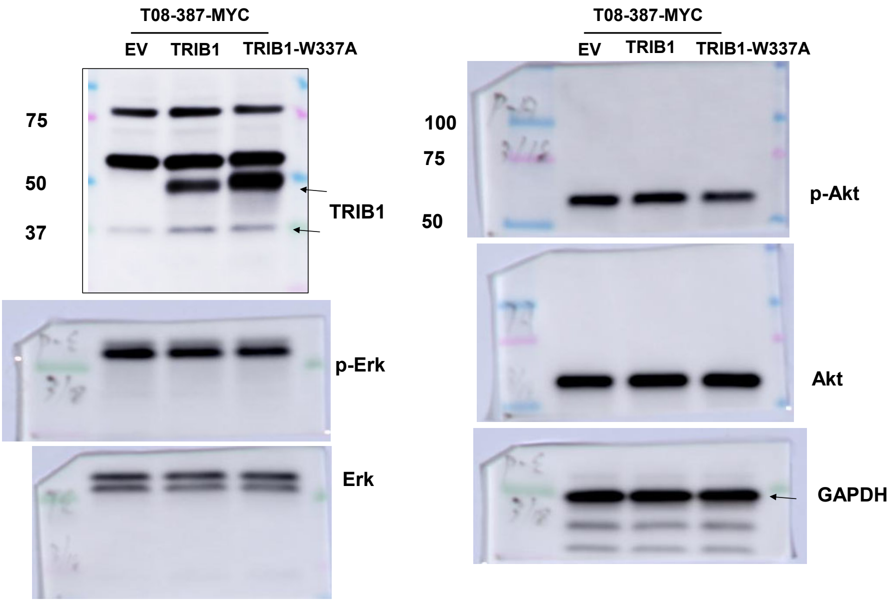

Replicate 1

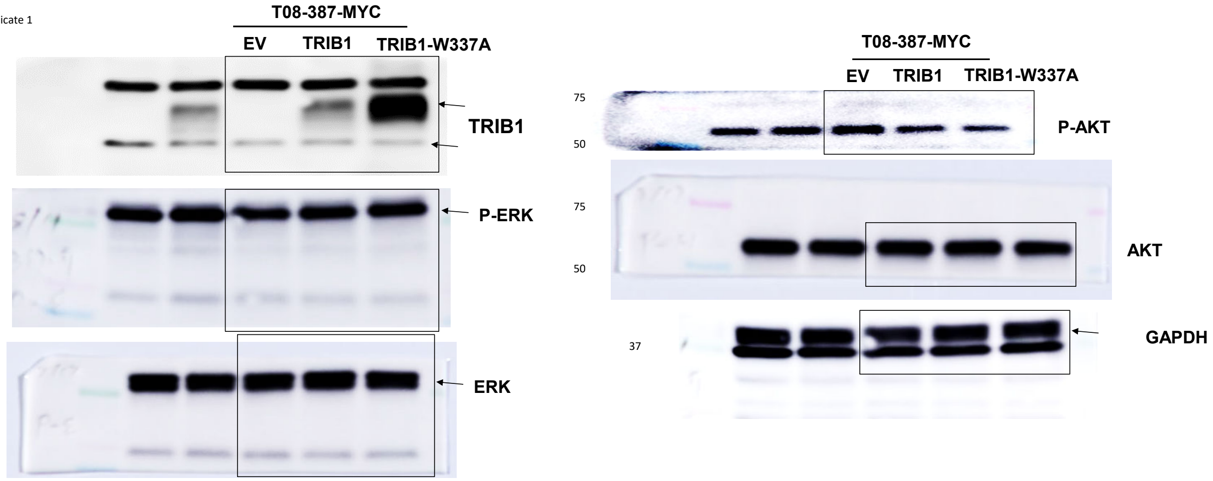

Supplementary fig. 6b

U87 MG cyclin E1, CDT1

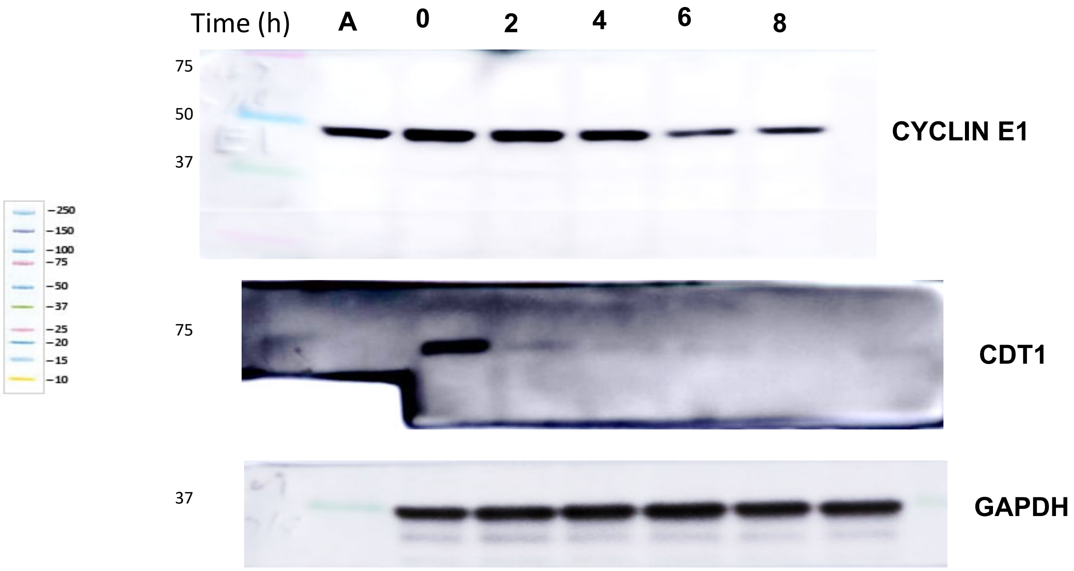

Replicate 1

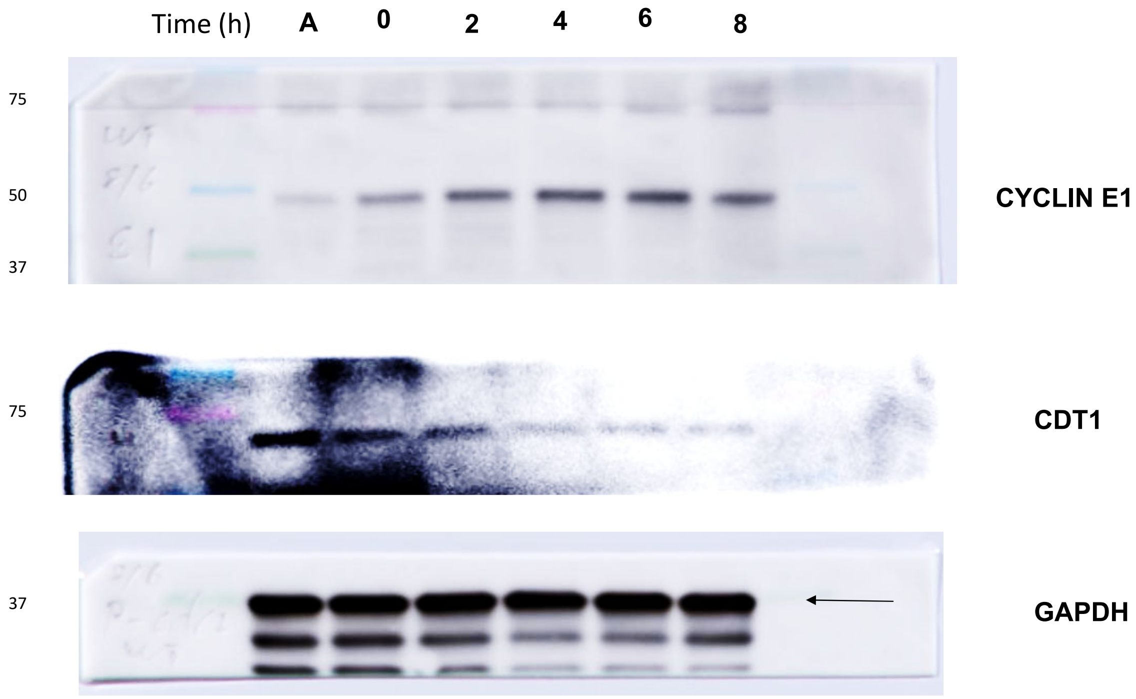

Supplementary fig. 6e T08-387 COP1, P53

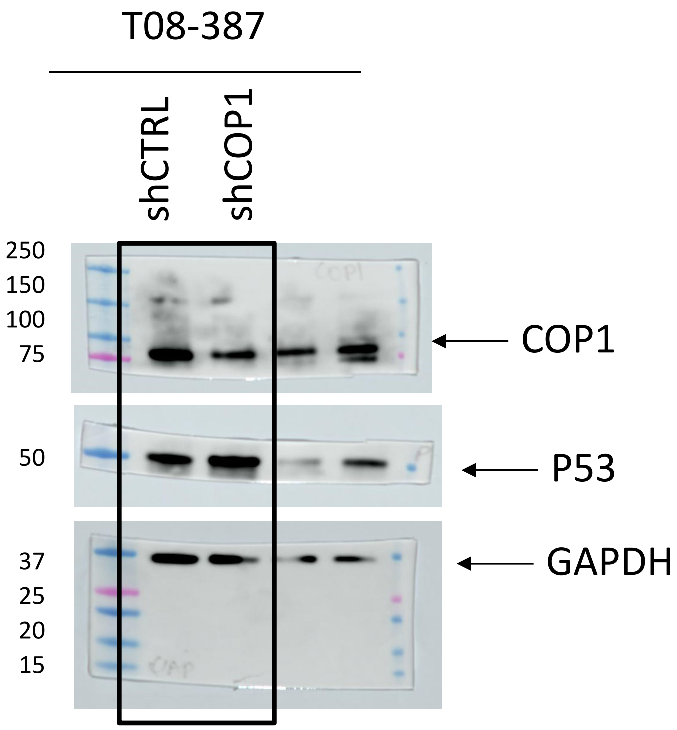

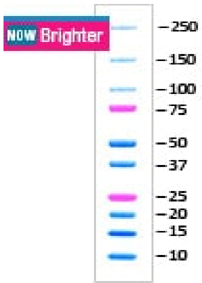

Replicate 1

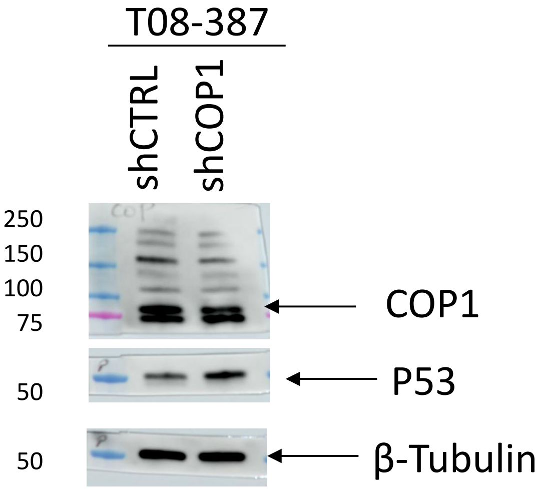

Supplementary fig. 6f LN18 TRIB1, P53, HDAC1

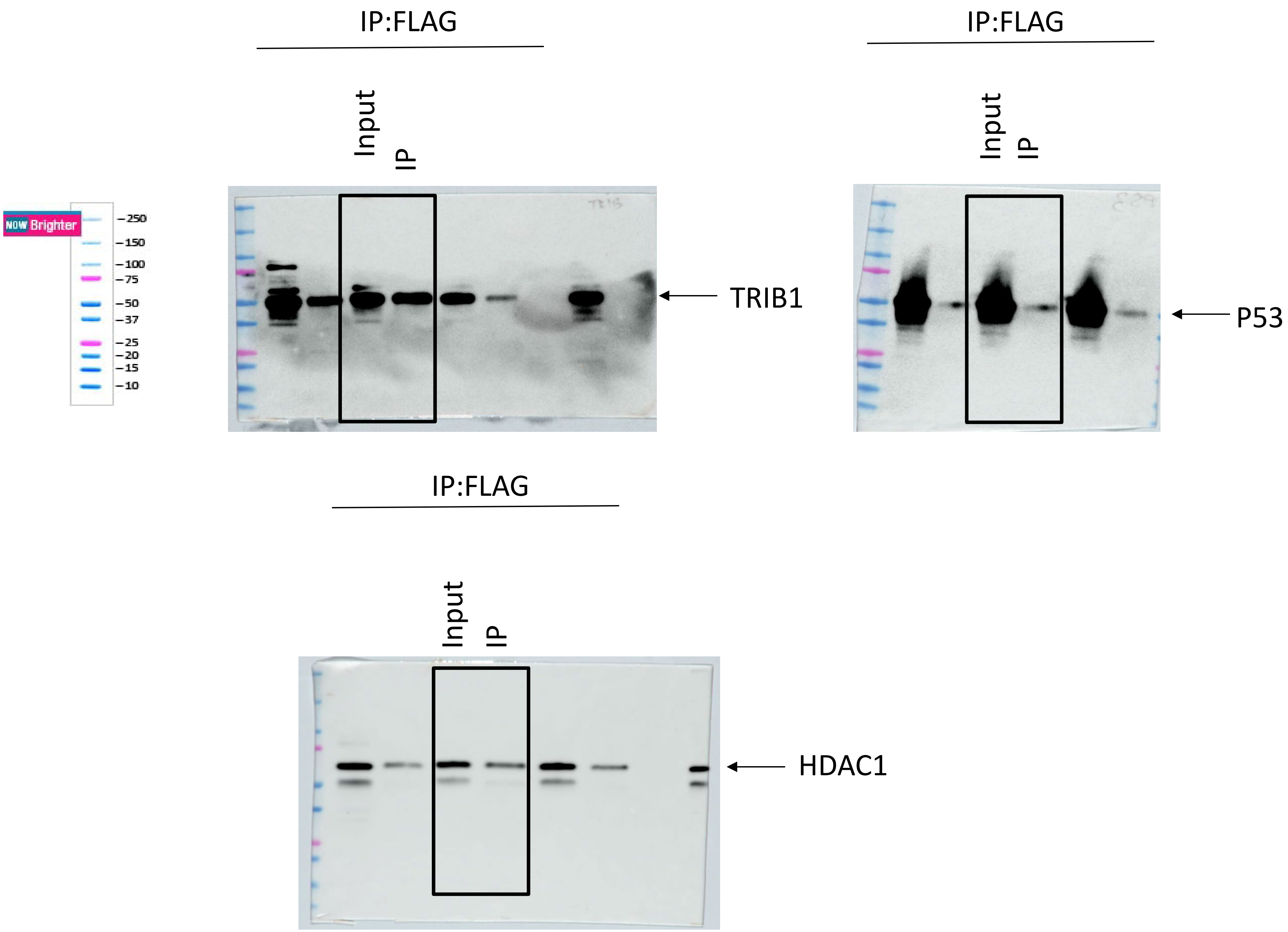

Freiburg dataset

Patient number	IDH_Status.CODE	OS_Event	OS_Months	TRIB1_Expression	TRIB_Med_dicho
1	mutant	0	46.44	4.878084	Below_Median
2	mutant	0	22.92	5.605165	Below_Median
3	mutant	0	25.92	4.723802	Below_Median
4	mutant	0	162.24	4.772838	Below_Median
5	mutant	0	14.28	5.67644	Below_Median
6	mutant	0	35.04	6.808716	Above_Median
7	wild-type	0	27.6	6.858755	Above_Median
8	mutant	0	45.36	4.933694	Below_Median
9	mutant	0	38.4	5.948219	Above_Median
10	mutant	0	33.84	6.077724	Above_Median
11	mutant	0	39	5.522792	Below_Median
12	mutant	0	46.8	5.188724	Below_Median
13	mutant	0	12.36	5.231255	Below_Median
14	wild-type	0	10.56	7.353078	Above_Median
15	mutant	0	273.48	4.285666	Below_Median
16	mutant	0	44.64	6.465479	Above_Median
17	wild-type	1	5.64	7.208908	Above_Median
18	mutant	0	1.8	6.437317	Above_Median
19	mutant	0	47.16	5.152324	Below_Median
20	mutant	1	27	7.038703	Above_Median
21	mutant	0	59.04	5.694125	Below_Median
22	mutant	0	225.84	4.842876	Below_Median
23	mutant	0	18.36	5.105207	Below_Median
24	wild-type	1	32.52	7.193826	Above_Median
25	mutant	0	24.12	5.092629	Below_Median
26	mutant	1	161.76	5.878605	Above_Median
27	mutant	0	31.08	7.204741	Above_Median
28	mutant	0	18.6	4.872146	Below_Median
29	wild-type	1	29.76	5.929092	Above_Median
30	mutant	0	34.08	6.361253	Above_Median
31	mutant	0	3	4.530057	Below_Median
32	mutant	0	4.2	6.088202	Above_Median
33	mutant	0	18.24	5.817215	Below_Median
34	wild-type	0	29.28	6.387474	Above_Median
35	mutant	0	39.6	6.038176	Above_Median

Utrecht dataset

Patient number	Suvival_Months	Event	cg00207280	cg00207280_Med_dicho
1	0.920547945	1	-3.421145062	Above_Median
2	11.17808219	1	-3.812838759	Above_Median
3	11.4739726	1	-3.78162123	Above_Median
4	31.06849315	1	-3.088462595	Above_Median
5	1.545205479	1	-3.996880954	Below_Median
6	7.561643836	1	-3.767813525	Above_Median
7	1.41369863	1	-3.226354216	Above_Median
8	25.41369863	1	-4.300305678	Below_Median
9	14.00547945	1	-4.584800893	Below_Median
10	44.35068493	1	-3.891664103	Below_Median
11	8.18630137	1	-3.856389168	Above_Median
12	6.21369863	1	-2.54072588	Above_Median
13	35.7369863	1	-4.472573589	Below_Median
14	1.150684932	1	-3.512669613	Above_Median
15	40.73424658	1	-4.263617631	Below_Median
16	8.449315068	1	-3.802787797	Above_Median
17	0.098630137	1	-4.273730629	Below_Median
18	18.14794521	1	-2.720301862	Above_Median
19	7.167123288	1	-4.498405993	Below_Median
20	4.734246575	1	-4.152530526	Below_Median
21	23.37534247	1	-4.501643577	Below_Median
22	0.065753425	1	-2.677738954	Above_Median
23	4.832876712	1	-4.9825688	Below_Median
24	6.805479452	1	-4.130187079	Below_Median
25	17.03013699	1	-4.146064877	Below_Median
26	12.03287671	1	-2.914924257	Above_Median
27	12.46027397	1	-4.390257497	Below_Median
28	4.964383562	1	-4.462048724	Below_Median
29	10.15890411	1	-4.095298504	Below_Median
30	23.14520548	1	-3.571221958	Above_Median
31	18.54246575	1	-4.228404077	Below_Median
32	11.90136986	1	-2.628694363	Above_Median
33	1.117808219	1	-3.838302439	Above_Median
34	12.36164384	1	-4.028641397	Below_Median
35	7.561643836	1	-3.513382439	Above_Median
36	0.854794521	1	-4.160910313	Below_Median
37	8.449315068	1	-4.244636452	Below_Median
38	21.27123288	1	-2.732139196	Above_Median
39	19.06849315	1	-4.513515068	Below_Median
40	5.78630137	1	-3.903585991	Below_Median
41	6.936986301	1	-3.071693729	Above_Median
42	26.1369863	1	-3.535140047	Above_Median
43	11.70410959	1	-3.957293256	Below_Median
44	10.42191781	1	-3.722734963	Above_Median
45	48.75616438	1	-4.680618838	Below_Median
46	2.071232877	1	-4.276418803	Below_Median
47	30.11506849	1	-3.549463733	Above_Median

48	22.98082192	1	-4.18057682	Below_Median
49	9.895890411	1	-4.380705242	Below_Median
50	9.468493151	1	-4.695543425	Below_Median
51	26.07123288	1	-4.330833893	Below_Median
52	62.33424658	0	-0.842753999	Above_Median
53	10.78356164	1	-4.635211461	Below_Median
54	13.97260274	0	-2.87631221	Above_Median
55	9.238356164	1	-4.622221227	Below_Median
56	17.3260274	1	-4.073616177	Below_Median
57	8.449315068	0	-3.62562924	Above_Median
58	4.471232877	1	-3.93060221	Below_Median
59	5.391780822	1	-4.030227646	Below_Median
60	14.1369863	1	-5.003488973	Below_Median
61	3.81369863	1	-4.25504016	Below_Median
62	36.85479452	1	-4.05306171	Below_Median
63	4.898630137	1	-4.372097179	Below_Median
64	17.03013699	1	-4.394088524	Below_Median
65	9.073972603	1	-3.440799606	Above_Median
66	14.33424658	1	-3.784699383	Above_Median
67	13.70958904	1	-2.949231364	Above_Median
68	2.728767123	1	-3.876554644	Above_Median
69	5.424657534	1	-4.495843572	Below_Median
70	66.80547945	1	-1.477032023	Above_Median
71	6.410958904	1	-3.857244824	Above_Median
72	4.010958904	1	-4.966670767	Below_Median
73	12.06575342	1	-3.739491417	Above_Median
74	10.52054795	1	-3.948692754	Below_Median
75	33.66575342	1	-4.162947263	Below_Median
76	57.3369863	0	-3.421252352	Above_Median
77	3.484931507	1	-4.053634256	Below_Median
78	3.81369863	0	-4.713057014	Below_Median
79	18.4109589	1	-3.672813691	Above_Median
80	20.15342466	1	-3.979611695	Below_Median
81	9.698630137	1	-2.860397093	Above_Median
82	10.91506849	1	-4.422520678	Below_Median
83	16.24109589	1	-4.431567338	Below_Median
84	1.578082192	1	-3.957004849	Below_Median
85	48.82191781	0	-0.268802547	Above_Median
86	5.391780822	1	-3.630317232	Above_Median
87	7.693150685	1	-2.870675022	Above_Median
88	24.23013699	1	-1.637961238	Above_Median
89	18.44383562	1	-3.772977551	Above_Median
90	3.879452055	1	-3.212833925	Above_Median
91	16.56986301	0	-4.343329153	Below_Median
92	28.10958904	1	-4.123849046	Below_Median
93	16.14246575	1	-4.1490665	Below_Median
94	5.621917808	1	-4.35303696	Below_Median
95	9.567123288	1	-4.19771916	Below_Median

96	4.734246575	1	-4.17251951	Below_Median
97	7.298630137	1	-2.991934652	Above_Median
98	17.95068493	1	-4.338258912	Below_Median
99	30.9369863	1	-3.477744201	Above_Median
100	14.26849315	0	-4.238078489	Below_Median
101	14.20273973	1	-3.344133544	Above_Median
102	6.378082192	1	-4.2665208	Below_Median
103	8.317808219	1	-4.337604225	Below_Median
104	6.77260274	1	-3.501951279	Above_Median
105	9.567123288	1	-4.176933194	Below_Median
106	54.50958904	0	-0.456637223	Above_Median
107	2.169863014	1	-3.928152019	Below_Median
108	11.01369863	1	-3.059007889	Above_Median
109	9.961643836	1	-4.911106106	Below_Median
110	3.517808219	0	-4.324709899	Below_Median
111	2.728767123	0	-3.857292729	Above_Median
112	39.25479452	0	-3.183864617	Above_Median
113	13.31506849	1	-3.622207715	Above_Median
114	6.443835616	1	-3.837724553	Above_Median
115	16.99726027	1	-3.525529734	Above_Median
116	36.13150685	0	-1.764235648	Above_Median
117	8.252054795	0	-3.756302989	Above_Median
118	2.761643836	1	-4.532700637	Below_Median
119	22.78356164	1	-5.283138778	Below_Median
120	13.38082192	1	-4.282447418	Below_Median
121	7.561643836	0	-3.496030182	Above_Median
122	32.02191781	1	-3.001727324	Above_Median
123	10.71780822	1	-3.086916334	Above_Median
124	3.484931507	1	-4.063714764	Below_Median
125	16.17534247	1	-4.189084179	Below_Median
126	6.378082192	0	-1.627391183	Above_Median
127	29.91780822	1	-4.087468303	Below_Median
128	5.095890411	0	-3.69484771	Above_Median
129	12.5260274	1	-4.353602875	Below_Median
130	0.361643836	0	-3.384009983	Above_Median
131	4.865753425	0	-3.290532587	Above_Median
132	6.64109589	1	-3.374994433	Above_Median
133	6.64109589	1	-3.591816879	Above_Median
134	13.08493151	1	-2.869651477	Above_Median
135	13.18356164	1	-3.548164346	Above_Median
136	0.756164384	1	-3.64483716	Above_Median
137	4.701369863	1	-3.801721132	Above_Median
138	34.45479452	0	-1.878948417	Above_Median
139	14.53150685	1	-3.536752796	Above_Median
140	25.77534247	0	-3.913444715	Below_Median
141	13.01917808	1	-4.108859745	Below_Median
142	22.78356164	0	-4.147373214	Below_Median
143	26.4	0	-4.181753223	Below_Median

144	0.493150685	1	-4.284700356	Below_Median
145	33.56712329	0	-2.698858015	Above_Median
146	2.465753425	1	-2.810742479	Above_Median
147	12.19726027	0	-4.138806534	Below_Median
148	28.5369863	0	-4.522765931	Below_Median
149	16.47123288	0	-4.565661788	Below_Median
150	21.04109589	1	-2.830459886	Above_Median
151	10.52054795	1	-3.576986186	Above_Median
152	13.11780822	1	-3.601778798	Above_Median
153	15.41917808	0	-2.984417137	Above_Median
154	17.0630137	0	-4.048496917	Below_Median
155	16.63561644	0	-3.702293075	Above_Median
156	14.46575342	0	-2.975534491	Above_Median
157	8.580821918	1	-3.870252566	Above_Median
158	12.59178082	1	-4.175776	Below_Median
159	16.10958904	0	-3.690535241	Above_Median
160	15.71506849	0	-3.889759454	Above_Median
161	16.50410959	0	-3.452951802	Above_Median
162	16.56986301	0	-3.677041823	Above_Median
163	15.41917808	0	-4.648053616	Below_Median
164	0.55890411	0	-4.300591265	Below_Median
165	12.85479452	1	-3.754428131	Above_Median
166	15.1890411	0	-2.987056447	Above_Median
167	14.16986301	0	-3.752007474	Above_Median
168	12.98630137	1	-3.231362684	Above_Median
169	10.81643836	1	-3.490163152	Above_Median
170	9.961643836	1	-3.679175246	Above_Median
171	11.34246575	0	-3.391902323	Above_Median
172	15.12328767	1	-4.640551953	Below_Median
173	12.13150685	1	-4.209479034	Below_Median
174	11.90136986	0	-4.487398011	Below_Median
175	12.75616438	0	-3.319495612	Above_Median
176	3.945205479	1	-3.627022736	Above_Median
177	9.665753425	1	-4.474316758	Below_Median
178	21.76438356	0	-4.408187255	Below_Median
179	12.85479452	1	-4.176442527	Below_Median
180	3.452054795	1	-4.370889131	Below_Median
181	21.30410959	0	-1.17589755	Above_Median
182	19.59452055	0	-2.99749717	Above_Median
183	5.621917808	1	-4.359207189	Below_Median
184	16.83287671	0	-3.274516951	Above_Median
185	11.44109589	0	-4.640698271	Below_Median
186	23.11232877	1	-4.661247802	Below_Median
187	9.534246575	1	-4.403476271	Below_Median
188	1.117808219	1	0.138305298	Above_Median
189	1.052054795	1	-4.029186262	Below_Median
190	10.84931507	0	-3.733023404	Above_Median
191	14.89315068	0	-3.649170026	Above_Median

192	14.16986301	0	-3.448576843	Above_Median
193	5.326027397	1	-3.504649018	Above_Median
194	15.15616438	1	-3.901714269	Below_Median
195	8.284931507	1	-4.458859719	Below_Median
196	11.11232877	1	-4.478405909	Below_Median
197	5.983561644	1	-4.107286371	Below_Median
198	1.380821918	0	-4.907528958	Below_Median
199	22.09315068	1	-4.069275381	Below_Median
200	0.263013699	0	-4.282923241	Below_Median
201	10.06027397	0	-3.671406833	Above_Median
202	0.131506849	0	-3.968421817	Below_Median
203	5.063013699	1	-3.833340968	Above_Median
